# Global disparities in association between leisure-time physical activity and chronic musculoskeletal pain: A systematic review and meta-analysis

**DOI:** 10.1016/j.ghrp.2026.05.002

**Published:** 2026-05-20

**Authors:** Elmo Wing-Yiu Lee, Chi Wai Cheung, Lin Wang, Fengfeng Wang, Hung Chak Ho

**Affiliations:** a Department of Anaesthesiology, School of Clinical Medicine, LKS Faculty of Medicine, The University of Hong Kong, Hong Kong Special Administrative Region of China; b Department of Public and International Affairs, City University of Hong Kong, Hong Kong Special Administrative Region of China; c Institute of Global Governance and Innovation for a Shared Future, City University of Hong Kong, Hong Kong Special Administrative Region of China

**Keywords:** Exercise, Chronic pain, Musculoskeletal pain, Meta-analysis, Geographic disparity, Demographic characteristics

## Abstract

**Background:**

Previous studies have extensively investigated the association between leisure-time physical activity (LTPA) and chronic musculoskeletal pain (CMSP). However, the results have been inconsistent. It is not known whether the differences in the association between LTPA and CMSP are due to underlying factors such as demographic patterns, geographical characteristics, and location(s) of pain.

**Methods:**

The systematic review and meta-analysis was conducted to assess global disparities in association between LTPA and CMSP**.** PubMed, EMBASE, MEDLINE, and Web of Science were searched for observational studies examining LTPA and the prevalence or incidence of CMSP in the general population. The systematic review with quality assessment using the JBI checklist and the Newcastle-Ottawa Scale was followed by a random-effects meta-analysis, including subgroup analysis using the following variables: sex, age group, geographical regions and location(s) of pain. Clinical significance of the pooled odds ratio (OR) was assessed using Cohen’s d and risk of publication bias was assessed using Egger’s test, funnel plot and trim-and-fill analysis.

**Results:**

Across 119 studies, the overall OR for the association between LTPA and CMSP averaged 0.78 (95% CI: 0.70–0.87, I2 = 99.5%) with no clinical significance (d = −0.137). Differences between genders were insignificant. Among age subgroups, adults and middle-aged/older adults showed a negative association between LTPA and CMSP (OR = 0.62, 95% CI: 0.47–0.82). Pain locations with reduced odds included unspecified pain locations (OR = 0.73, 95% CI: 0.62–0.86) and lower back (OR = 0.82, 95% CI: 0.69–0.98). Geographic subgroups with negative LTPA-CMSP associations included high-income countries (OR = 0.78, 95% CI: 0.70–0.88), the continents of North and South America and Europe, and the regions of Northern and Western Europe. The differences between continents and between subcontinental regions were both significant (*p* = 0.0211 and *p* = 0.0260, respectively), suggesting an influence of sociocultural factors in addition to income level.

**Conclusions:**

LTPA was inversely associated with CMSP, especially in selected populations and pain locations. This association could be due to underlying factors, such as local factors that characterize LTPA in different countries. These findings suggest that the benefits of LTPA cannot be considered universally applicable. Locally and regionally tailored interventions should be developed, taking into account place-specific structural, economic and social characteristics.

## Introduction

Chronic pain is one of the greatest global health burdens,^1^, ^2^ reaching a prevalence of 20% worldwide and accounting for 15%-20% of physician visits.^3^ Chronic musculoskeletal pain (CMSP) is an important chronic pain problem, particularly in low- and middle-income countries. A prevalence of 21% (15–27%) for low back pain and 25% (19–33%) for CMSP was found among the general population in low- and middle-income countries.^4^ The prevalence may be even higher among older adults and females.^5^, ^6^ CMSP can last longer than 3 months or recur and lead to significant disability.^7^

Leisure time physical activity (LTPA) is recognized as a non-pharmacological intervention that could reduce CMSP.^8^, ^9^ Formally defined as “physical activity performed during exercise, leisure time, or at a time other than that associated with regular work, housework, or transportation”,^10^ LTPA interventions (e.g., planned and repetitive exercise) have been shown to reduce CMSP. These LTPA interventions can improve or maintain physical fitness or other health benefits,^11^ ultimately leading to a reduction in CMSP. A review of 12 studies found that LTPA interventions can reduce chronic pain in people with spinal cord injury.^12^ This is different from occupational physical activity in the workplace, which can be associated with excessive repetition, awkward postures, and heavy lifting, leading to painful work-related musculoskeletal disorders.^13^ As a lifestyle modification, LTPA also minimizes the cost of monitoring by healthcare professionals.

Although LTPA is a common intervention, the relationship between LTPA and CMSP and the differences between populations has been poorly studied. The results in published systematic reviews and meta-analyses on LTPA and CMSP have been inconsistent.^14^, ^15^, ^16^ This is important to understand because certain subgroups (e.g., older adults) often suffer from comorbidities and frailty, which limits the scope of long-term care. Living in different locations (high-income countries versus low- and middle-income countries) could also influence LTPA due to differences in cultural practices, lifestyle, socioeconomic status, and environmental conditions. These differences have not been explored in previous studies, including published systematic reviews and meta-analyses on LTPA and CMSP.^14^, ^15^, ^16^ More importantly, the association between LTPA and CMSP at sites other than the low back has been poorly investigated.^17^, ^18^

In this study, a systematic review and meta-analysis based on the existing literature was conducted to comprehensively investigate the association between LTPA and CMSP. In addition to clarifying the overall association between LTPA and CMSP in the general population, our main objective is to evaluate whether the association between LTPA and CMSP could be due to differences in gender, age, geographic characteristics, and pain location(s). The results could be useful to demonstrate the importance of the association between LTPA and CMSP among subgroups for public health surveillance in a global context.

## Methods

### Search strategy

This study was conducted according to the guidelines of the Preferred Reporting Items for Systematic Reviews and Meta-Analyses (PRISMA). The study was registered with PROSPERO (ID: CRD42023400652). Screening of the obtained records, data extraction and risk of bias assessment for each study were performed by 2 reviewers working independently, with disagreements resolved by discussion.

A literature search was conducted in the PubMed, EMBASE, MEDLINE and Web of Science databases on April 1, 2023. It included all human observational studies that examined LTPA levels and the prevalence of CMSP in the general population or a presumably healthy subgroup of the general population. All papers with English abstracts were scanned. To increase sensitivity, a relatively broad search strategy was used (Table 1) in which chronicity of pain and aspect of physical activity (PA) were not specified (e.g. transportation, occupational, recreational, domestic, competitive).

**Table 1:** Standard subgroup for meta-analysis.

**Group**	**Variable**	**Ideal default value**
Study design	Study design	Cohort
Population	Gender	Mixed (male + female)
	Age group	Adult (≥18 years-old)
	Occupation	General public
Geographics	Region	Worldwide
LTPA	Assessment	Metabolic Equivalent of Task (MET) based (with LTPA intensity assessed)
	Level	3–4 days/week, totaling 150 min/week moderate-to-vigorous physical activity
	Intensity	Moderate-to-vigorous (causing increased heart rate, increased breathing, and mild sweating)
	Nature	Leisure: domestic, transportation, or sports
CMSP	Assessment	Any (including substantially modified Nordic Musculoskeletal Questionnaire [NMQ])
	Location	Any body region
	Duration	≥ 3 months, or 1 month for Health-Related Quality of Life (HRQoL) questionnaires
	Frequency	At least once/week
Statistics	Confounders	Maximally adjusted
	Format	Adjusted Odds Ratio (aOR) with 95% CI

### Selection criteria

Chronicity of CMSP was defined as persistence of pain beyond the normal healing period.^19^ Inclusion criteria included: (a) observational studies of subjects or patients with CMSP and healthy controls; (b) assessment of LTPA or other assessments of PA in which LTPA is a dominant component; (c) Assessment of the prevalence of CMSP at sites such as “unspecified”, “back”, “lower back”, “neck/shoulder” or “multiple sites” as in chronic widespread pain and fibromyalgia; (d) Specification of a mathematically valid association between LTPA performance and CMSP as odds ratio (OR) and 95% confidence interval (CI) or convertible to these.

To increase the specificity of observational studies on CMSP, exclusion criteria included: (a) English full texts with an adequate description of the methodology were not accessible; and (b) the LTPA assessment included a very limited list of physical exercises, such as only focusing on yoga or running, but not all other examples of physical exercises. On the other hand, studies were not excluded if transportation and/or domestic activities were included in the assessment of the level of LTPA, as the boundaries between LTPA and other non-occupational physical activities are often blurred.^20^

### Data extraction

The extracted data include: (a) study details, including epidemiological design, sample size, and country); (b) demographic factors, including age and sex; (c) weekly LTPA level (in terms of frequency, total duration, or metabolic equivalent of task) used for dichotomization; (d) location of CMSP studied; (e) methodology of assessment of LTPA and CMSP; (f) health consequences of CMSP by LTPA estimated by OR with 95% CI; and (g) list of confounders adjusted for in the statistical analysis. Risk ratios were converted to ORs with corresponding 95% CI using the assumed comparative risk (ACR),^21^ preferably from the same study or from another included study with a similar sample population composition. Other measures of effect size were converted by calculating the log-odds and their standard error. For studies that reported the median and interquartile range (IQR) of two groups for comparison, median and IQR were converted to mean and standard deviation (SD) to derive log-odds.^22^

For studies that reported only the effect sizes and standard errors of mutually exclusive subgroups, the overall effect size and standard error were calculated from averaging the subgroup data using the random-effects model as the standard dataset for the meta-analysis. If the subgroup data are based on non-exclusive population samples, a pair of effect size and standard error is selected as default. The standard selection was noted in Table 1.

Age groups were categorized as follows: “adults” (≥18 years), “children” (≤13 years), “adolescents” (10–18 years), “young adults” (18–28 years), “older adults” (≥45 years), and “all” (across all age groups). Countries were categorized into geographic “regions”, including “Worldwide”, “Central Asia”, “Central Europe”, “East Asia”, “Eastern Europe”, “Middle East”, “North Africa”, “North America”, “Northern Europe”, “Oceania”, “South America”, “South Asia”, “Southeast Asia”, “Southern Europe”, “Sub-Saharan Africa” and “Western Europe”. The weekly frequency of LTPA, was recorded as a whole number between 1 and 7, indicating the number of days with at least 45 min of LTPA of at least moderate intensity. Extremely high thresholds, such as 2 h of vigorous LTPA per day, were also counted as 7 days/week. For the “location” of pain, the “neck/shoulder” group included neck pain, shoulder pain, and neck/shoulder pain.

### Assessment of the quality of evidence

The Newcastle-Ottawa Scale (NOS) 23, 24 for included case-control and cohort studies. A score was assigned to each of the assessed domains of selection, comparability and outcome (Maximum total score = 9). Higher scores of 4, 2 and 3, respectively, indicate a lower risk of bias in each domain. The JBI checklist^25^ was used for the included cross-sectional studies. The checklist consists of 8 questions to assess the research methodology and the adequacy of statistical reporting, with negative responses indicating a risk of bias.

### Data analysis

Included studies were used for the meta-analysis, which was performed with the “R package "metafor".^26^ The pooled OR of LTPA-CMSP, 95% CI and p-value describing the association of the included studies were aggregated as the primary outcome using the random effects model modified by Knapp and Hartung.^27^ The restricted maximum likelihood (REML) heterogeneity variance estimator was used.^28^ The secondary outcome is obtained from the subgroup analysis subsequently conducted, in which demographic characteristics, location(s) of pain and geographical characteristics were used as proxies for the general socioeconomic status of the surveyed population. 3 levels of geographical classification were used, including country's income level, geographical continent and finally, for Africa, Asia and Europe, subcontinental regions. Such planning was supported by the presence of significant variations in the Human Development Index (HDI) between continents.^29^

Heterogeneity was assessed using the I-squared statistic.^30^ Clinical significance was assessed using Cohen’s d, where 0.2, 0.5 and 0.8 were considered lower bounds for a small, moderate and large effect size, respectively, and d< 0.2 implied clinical insignificance.^31^ d= -0.2 corresponds approximately to OR = 0.70. Differences in effect size (based on OR) describing the relationship between LTPA and CMSP in the different subgroups were tested using a Wald test.^32^

A funnel plot^33^ and Egger’s test^34^ were applied to assess the risk of publication bias, with *p* < 0.1 indicating the presence of publication bias.^35^ If publication bias was detected, a trim-and-fill analysis was performed using the R_0_ estimator^36^ to estimate the number of possible missing studies and recalculate the pooled effect size taking into account the missing studies.

## Results

### Search results, study characteristics and overall meta-analysis

**Fig. 1:**
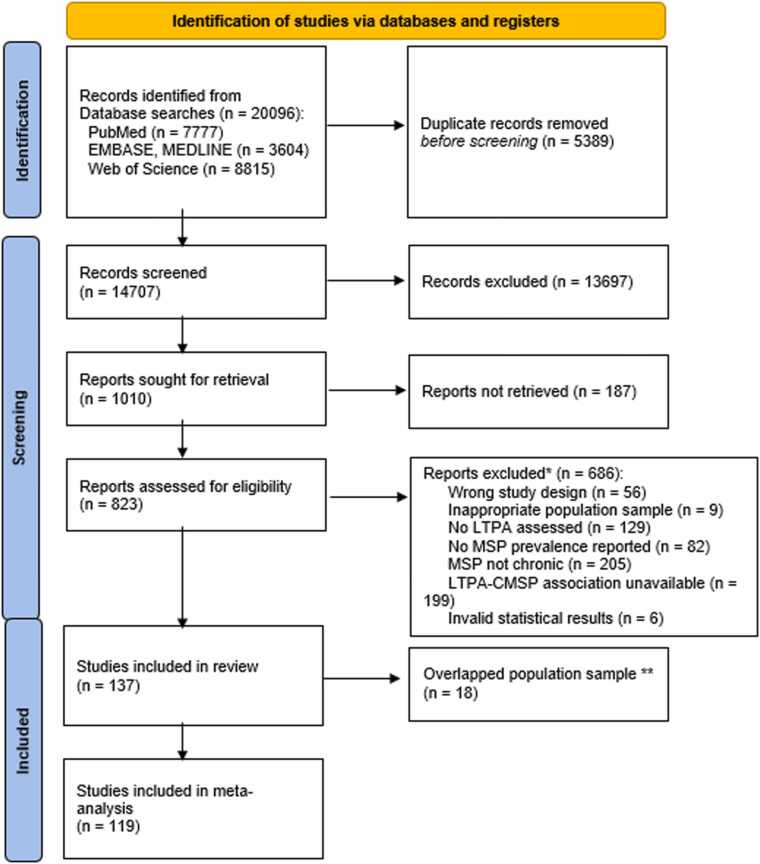
PRISMA flowchart. * **Wrong study design:** animal or human interventional studies; **Inappropriate population sample:** only studying patients with a specific disease which could cause pain, e.g. rheumatoid arthritis, or not enrolling any pain-free participants; **No LTPA assessed:** the level physical activity assessed, if any, does not include LTPA; **No MSP prevalence reported:** the study did not report the prevalence of musculoskeletal pain (MSP) separately in physically active and inactive groups; **MSP not chronic:** studied the prevalence of MSP over a period less than 1 month, or 4 weeks; **No appropriate LTPA-CMSP association analysis:** the statistical reporting regarding of the LTPA-CMSP association identified could not be extracted as, or converted to, odds ratio with 95% confidence intervals, e.g. linear regression coefficients without the standard deviation of either variable analysed; **Invalid statistical results:** mathematically invalid statistical results which cannot be fully accounted by rounding errors. ** Multiple studies published based on similar secondary analysis of the same data set, such as the HUNT study.

The search in the database yielded 20,096 records, including 5388 duplicates. After a title/abstract screening and a full-text review, 137 studies were finally included (Supp. Table 2). Details of the above process were presented in PRISMA flowchart (Fig. 1).^37^ The data extracted from the included studies and the results of the risk of bias assessments were tabulated (Supp. Table 2). 18 included studies were excluded from the meta-analysis because their samples overlapped heavily with other studies. For example, Mork et al. and Nilsen et al. both examined the association between physical activity and chronic low back pain using data from the HUNT1 and HUNT2 studies.^38^, ^39^ Of the 137 studies included in the systematic review, sample sizes ranged from 28 to 242,103 subjects, and 27.7% of included studies (n = 38) were without adjustment for potential confounders on the effect size of interest.

**Fig. 2:**
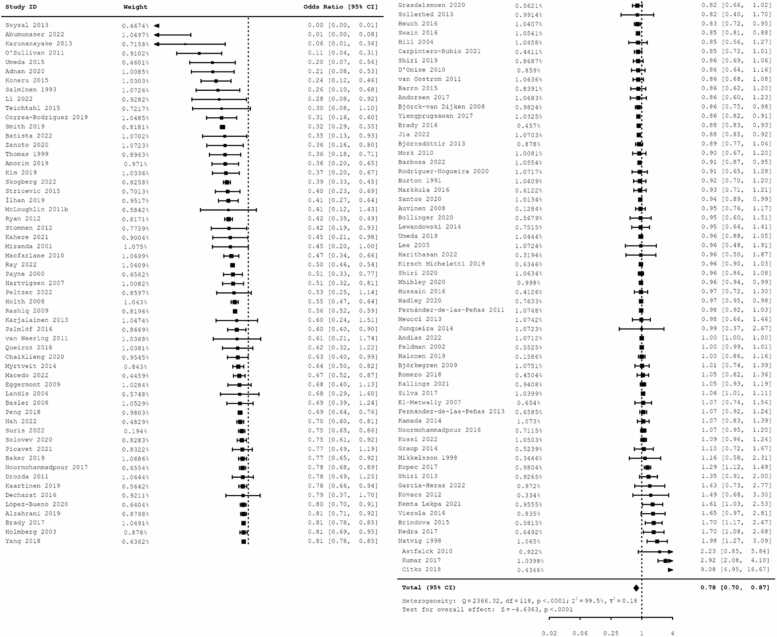
Forest plot – main analysis. A pooled odds ratio with 95% confidence interval was calculated by the random-effects model modified by Knapp and Hartung. CI, confidence interval.

Fig. 2 presents the forest plots for the overall meta-analysis. Overall, the meta-analysis of 119 studies (Fig. 2) found that higher participation in LTPA was associated with a reduction in the likelihood of CMSP. However, the overall association (Table 2) was highly heterogeneous and not of clinical significance (OR = 0.78, 95% CI = 0.70–0.87, *p* < 0.0001, I^2 = 99.5%, d = −0.137).

**Table 2:** Summary of meta-analysis results. For the overall meta-analysis and for each subgroup under each variable used in the subgroup analysis, the pooled odds ratio with 95% confidence interval as well as the I^2 heterogeneity statistic were reported. The Q-statistic and degree(s) of freedom were reported for each inter-subgroup comparison performed using the Wald-type test.

Variable / subgroup	N^#^	OR (95% CI), I^2^	Q, df	p-value^
**Overall**	119	0.78 (0.70–0.87), 99.5%		**< 0.0001**
**Sex**			2.0061, 2	0.3668
Male	23	0.85 (0.79–0.91), 66.4%		**< 0.0001**
Female	26	0.85 (0.80–0.91), 41.5%		**< 0.0001**
Mixed-sex samples	107	0.78 (0.70–0.87), 99.5%		**< 0.0001**
**Age group**			12.6345, 5	**0.0271**
All ages	2	0.80 (0.23–2.73), 41.5%		0.2601
Children	7	1.31 (0.82–2.08), 78.6%		0.2083
Adolescents	17	0.83 (0.64–1.09), 99.8%		0.1683
Adults	70	0.80 (0.70–0.91), 98.3%		**0.0008**
Younger adults	5	0.97 (0.70–1.35), 68.7%		0.8025
Middle-aged/older adults	23	0.62 (0.47–0.82), 97.9%		**0.0017**
**Location of pain**			4.5327, 4	0.3387
Not specified	38	0.73 (0.62–0.86), 99.7%		**0.0006**
Back*	15	0.90 (0.69–1.16), 95.7%		0.3747
Neck and shoulder	17	0.51 (0.26–1.00), 99.7%		0.0501
Lower back	51	0.82 (0.69–0.98), 97.3%		**0.0262**
Multisite	11	0.68 (0.44–1.07), 87.1%		0.0882
**Country income level**			0.1216, 1	0.7273
High-income	94	0.78 (0.70–0.88), 99.6%		**< 0.0001**
Low-/Middle-income	25	0.74 (0.54–1.01), 98.8%		0.0594
**Geographical continents**			14.8959, 6	**0.0211**
Africa	5	0.93 (0.53–1.66), 94.1%		0.7568
Asia	16	0.41 (0.17–1.02), 99.8%		0.0546
America (North)	17	0.70 (0.57–0.86), 99.2%		**0.0024**
America (South)	10	0.91 (0.87–0.96), 0.0%		**0.0042**
Europe	62	0.81 (0.73–0.94), 98.0%		**0.0042**
Oceania	8	0.74 (0.38–1.43), 98.9%		0.3109
Europe and North America	1	0.85 (0.81–0.88), 0.0%		**< 0.0001**
**Sub-continental regions**			23.2112, 12	**0.0260**
North America	17	0.70 (0.57–0.86), 99.2%		**0.0024**
Northern Europe	30	0.84 (0.74–0.96), 89.4%		**0.0110**
Western Europe	15	0.65 (0.51–0.83), 95.6%		**0.0019**
Southern Europe	11	0.86 (0.66–1.10), 94.1%		0.2040
Central Europe	6	1.40 (0.47–4.20), 94.9%		0.4687
Middle East	5	0.11 (0.003–3.76), 99.8%		0.1560
East Asia	5	0.81 (0.61–1.09), 76.7%		0.1219
Southeast Asia	3	0.82 (0.58–1.16), 21.4%		0.1310
South Asia	3	0.39 (0.0031–50.10), 96.5%		0.4934
Sub-Saharan Africa	5	0.93 (0.53–1.66), 94.1%		0.7568
South America	10	0.91 (0.87–0.96), 0.0%		**0.0042**
Oceania	8	0.74 (0.38–1.43), 98.9%		0.3109
Europe and North America	1	0.85 (0.81–0.88), 0.0%		< 0.0001

N: number of studies, OR: odds ratio, CI: confidence interval, df: degree(s) of freedom

^ Statistically significant results (p-value < 0.05) were bolded.

* The area “back” spans from the posterior neck to the lumbar back.

# Numbers in each variable may not add up as some studies reported data in multiple subgroups

### Subgroup analyses for assessing disparities in age, gender, and location(s) of pain

Our forest plot's results show that no significant difference was found in the LTPA-CMSP association between male, female and mixed-gender individuals (Supp. Figure 1). Despite the inclusion of some studies that focused on male-dominated occupations, including long-distance truck drivers and firefighters,^40^, ^41^ gender-specific data were relatively limited compared with mixed-gender samples. LTPA was favored in all groups, but no clinically significant figure was identified (males: d = −0.090; females: d = −0.090; mixed-gender: d= −0.137).

The difference in the LTPA-CMSP association between the age groups was significant (Supp. Figure 2). Data are relatively abundant in the adult age group (age≥18) compared to the adolescents (age: 11–18) and children (age≤13). The middle-aged/ older adults (age ≥ 45) showed a negative association (OR = 0.62, 95% CI: 0.47–0.82) with low clinical significance (d = −0.264). LTPA was similarly associated with lower odds of CMSP in adults (d = −0.123). Interestingly, 2 of 7 pediatric studies showed a positive association between LTPA and CMSP. This proportion was much higher than in other age groups, resulting in a deviation of the aggregated effect size in favor of physical inactivity. However, two studies included in the meta-analysis that examined both adults and minors reported contradictory results. Specifically, Surís et al.^42^ found a negative LTPA- CMSP association in individuals aged ≥ 15 years (OR = 0.75, 95% CI: 0.65–0.86, *p* < 0.0001, d = –0.160), while Burton & Tillotson found the association to be non-significant (OR = 0.92, 95% CI: 0.70–1.20, *p* = 0.2662) based on 958 individuals aged 10–84 years, with children aged 10–11 years accounting for 28%.^43^ Therefore, the pooled result from these studies was also not significant (OR = 0.80, 95% CI: 0.23–2.73, I^2 = 41.5%, *p* = 0.2601).

Regarding the location(s) of CMSP, most of the included studies either did not report a specific pain site or focused on low back pain. Several included studies were on neck and shoulder pain (n = 17), “anywhere in the back” ranging from the posterior neck to the lumbar back (n = 15), and pain at multiple sites, such as fibromyalgia (n = 11). Pain locations with reduced odds included unspecified pain locations (OR = 0.73, 95% CI: 0.62–0.86) and lower back (OR = 0.82, 95% CI: 0.69–0.98). However, the odds reduction for CMSP at an unspecified pain site and in the lower back was not clinically significant (unspecified: d = −0.174; lower back: d = −0.109). No odds reduction was found “anywhere in the back”, even for pain at multiple sites, while the odds ratio at the neck/shoulder sites was close to statistical significance (OR = 0.51, 95% CI: 0.26–1.00, *p* = 0.0501). The difference between the body sites was not significant (Supp. Figure 3).

### Subgroup analyses for assessing geographic disparities

In terms of income level, the meta-analyzed studies were mostly from high-income countries, for example from the USA (n = 13) and Finland (n = 12), and only 25 from low/middle-income countries. In contrast to high-income countries (OR = 0.78, 95% CI: 0.70–0.88), no significant association between LTPA and CMSP was found in low/middle-income countries (high-income countries: d = −0.137). Overall, the difference between high-income and low/middle-income countries was not significant (Supp. Figure 4). However, there was a significant intercontinental difference. The negative LTPA-CMSP associations in North America, South America and Europe did not reach clinical significance (d = −0.197, d = −0.052 and d = −0.116, respectively) (Supp. Figure 5). Remarkably, a multinational study covering Europe and North America was also included in the meta-analysis (OR= 0.85, 95% CI: 0.81–0.88, d = −0.090).^44^

Subgrouping by subcontinental regions, there are few included studies in Southeast Asia (n = 3), South Asia (n = 3) and sub-Saharan Africa (n = 5), despite the large populations in these regions. More importantly, no data were collected specifically from Central America, Eastern Europe, North Africa or Central Asia. There was a significant difference between the studies in the different subcontinental regions. Negative LTPA-CMSP associations were found in North and South America and in Northern and Western Europe, with only Western European studies reaching clinical significance (Western Europe: d=-0.238, Northern Europe: d=-0.096) (Supp. Figure 6).

### Risk of biases

The 55 included cohort and case-control studies generally had a low to moderate risk of bias. Among them, 31 studies scored 8 or above and 19 studies scored 5–7 in NOS. 5 studies had a moderate risk (score = 4). The 82 included cross-sectional studies generally had a low to moderate risk of bias in the JBI checklist. Of the 8 items assessed in the checklist, the 4th item “Were objective, standard criteria used for measurement of the condition?” was generally not applicable as all studies assessed were from a presumably healthy general population as defined in the inclusion criteria. 15 studies met at least 6 of the remaining 7 items, 49 studies met 4–5 items; however, 12 studies met only 2–3 items. Lack of comparison of demographic data between the sample population and the total target population of interest was the main source of risk of bias in cross-sectional studies.

In general, most included studies had a moderate risk of bias. A minority of included studies had a high risk of overall bias. Regardless of study design, the use of unvalidated assessment questionnaires for LTPA and CMSP was a source of outcome bias in the results. The lack of adjustment for confounding factors led to a risk of comparability bias in 12 cohort and case-control studies and in 27 cross-sectional studies.

**Fig. 3:**
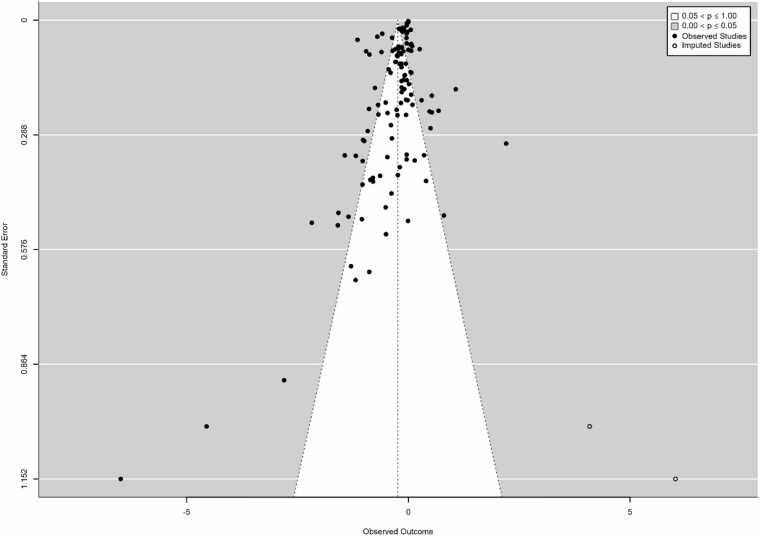
Funnel plot of main analysis after trim-and-fill. Odds ratios (Observed Outcome) with 95% confidence intervals (converted to Standard Errors) plotted in Forest plot 1, as well as missing data points (“Filled studies”) calculated by trim-and-fill analysis using R_0_ estimator were included in this plot.

Regarding publication bias, the funnel plot of the main analysis showed that most studies had low standard errors (Fig. 3). Despite the few data points favoring physical activity with high standard errors, there was an obvious asymmetry skewed to the right, possibly causing the risk of publication bias in the Egger’s test (t = -5.4297, df = 117, *p* < 0.0001). Trim-and-fill analysis identified 2 potentially missing data favoring physical activity. The adjusted overall OR for the association between LTPA and CMSP was 0.79 (95% CI: 0.72–0.86, *p* < 0.0001). It remained clinically non-significant (d = −0.131) and highly heterogeneous (I^2 = 99.6%).

## Discussion

This is the first study to systematically examine the disparities in the association between LTPA and CMSP specifically for different pain sites and geographic regions. Our results showed a statistically significant overall OR of 0.78, which is consistent with previous systematic reviews that reported pooled ORs ranging from 0.77 to 0.87.^17^, ^18^ It is important to note that the use of the REML estimator in this study reduced statistical bias in the calculation of between-study heterogeneity^45^, ^46^ and that the Knapp-Hartung adjustment reduced the type I error rate in the random-effects model and incorporated heterogeneity into the derivation of the 95% confidence intervals of the pooled ORs.^27^ Thus, despite the large heterogeneity we found, our results of pooled ORs with 95% confidence intervals can be considered a relatively accurate reflection of the magnitude and certainty of the samplewise association between LTPA and CMSP.

Compared to previously published meta-analyses, our data showed a higher degree of heterogeneity. This is consistent with our hypothesis that the association between LTPA and CMSP reported in previous publications may be influenced by several underlying factors at play in the included studies, including demographic characteristics, socioeconomic status, and cultural differences.

Age and gender are the two demographic factors examined in the current study. Traditionally, it has been emphasized that females are more prone to CMSP,^6^, ^47^ however, in our study, no significant difference in the association between LTPA and CMSP was found between both genders. Therefore, the higher prevalence of CMSP in females might be mainly due to their lower LTPA levels compared with males.^48^ Another possible explanation is that the gender differences in the association of LTPA and CMSP might be influenced by several factors (e.g., lifestyle, age, comorbidities). For example, Chou et al. reported that in addition to "females", blue-collar jobs and osteoporosis are risk factors for low back pain.^6^ Among the older ages, Ho et al. reported a greater positive association between LTPA and CMSP in females.^49^ Further research in this direction is beyond the scope of the present study.

Regarding the significant age differences in the LTPA-CMSP association, the statistical insignificance and large heterogeneity of the LTPA-CMSP association in children, adolescents and younger adults may be due to the limited number of included publications. In contrast to older groups, pain problems in children, adolescents and younger adults have been found to be associated with obesity (or body weight), which could modify the association between LTPA and CMSP.^50^ However, obesity in children, adolescents and younger adults are highly influenced by their genes, racial background, lifestyle and cultural practices,^51^, ^52^, ^53^, ^54^, ^55^, ^56^ although heterogeneity was not controlled for in this study. Only 2 included studies on children used body weight as a confounder, which further reduces the quality of the pooling effect.

The significant results found in middle-aged/older adults could possibly be expressed as some degree of causality. Previous studies have frequently emphasized that a sedentary lifestyle can lead to hyperalgesia in older adults,^57^, ^58^ while LTPA was a protective factor not only for pain but also for other related conditions, including dementia and bone fractures.^59^, ^60^ From the Norwegian HUNT2 and HUNT3 studies,^61^ 1–2 h of strenuous physical activity per week reduced the risk of chronic low back pain 11 years later in both males and females aged 50–69 years who were pain-free at baseline, even after adjustment for education, work status, physical activity at work, body mass index and smoking habits.^62^ The effect of LTPA in older adults has also been highlighted in global guidelines such as “The WHO Age-friendly Cities Framework” to promote healthy aging in place. Our findings support this global fact, although the underlying mechanisms between LTPA and CMSP may differ.

Regarding the location of CMSP, the magnitude of the association between LTPA and CMSP was greater in an unspecified pain site or “neck/shoulder” area" than in the lower back, suggesting that LTPA in both the lower back and extremities is associated with a lower likelihood of CMSP. Nauta et al. found that physical inactivity predisposed to upper extremity injuries.^63^ Toivanen et al. found a protective effect of regular LTPA against knee osteoarthritis.^64^ Insignificant LTPA-CMSP associations were found for the “somewhere in the back” area, which is consistent with the upper back regions causing a deviation of the LTPA-CMSP association in favor of physical inactivity or insignificant LTPA-CMSP associations. This was confirmed by another study using objective measures of physical activity and pain.^65^ The current study provided additional evidence of a difference in the LTPA-CMSP association between the upper and lower back regions.

The insignificant difference between high-income and low/middle-income countries suggests a mixed effect on the association between LTPA and CMSP. On the one hand, the data from high-income and low/middle-income countries showed a similar proportion of deviating data points in both directions of effects. Clinically insignificant results from the studies from low/middle-income countries could therefore be mainly due to the limited publications leading to high heterogeneity. On the other hand, these discrepant data points could be due to a mixture of countries with different settings in each group. In particular, the terms “high income” and “low/middle income” over-generalize the geographical characteristics of the different countries. The health/social system, cultural practices/lifestyles and urban forms/built environment can be completely different in the countries in this group. Thus, if there are underlying factors that influence the relationship between LTPA and CMSP in individual countries, the pooled results would be insignificant. For example, despite a global downward trend in opioid use, the United States has seen an increasing use of opioid analgesics, which has led to an "opioid epidemic" that is undermining pain treatment and the health care system.^66^ When grouping studies from the United States with studies from other nearby countries (e.g., Canada or South American countries), this underlying factor may influence the pooled results. The insignificant intercontinental variation further supported this assumption.

The significant heterogeneity of the association between LTPA and CMSP observed in different geographical regions of the subcontinent may be due to the clinical heterogeneity of the included studies. In particular, we were only able to draw significant conclusions from some of the regions studied. The social and built environment in these regions was more homogeneous, especially in Northern Europe, Western Europe and North America, so we could observe similar types of LTPA and lifestyles that could influence chronic pain in a similar way. In contrast, regions with a more complex social/built environment or with a limited number of studies would produce inconsistent results. For example, 3 of the 5 sub-Saharan African studies were from South Africa, while 2 others were from Cameroon and Benin respectively. The health/social system, cultural practices/lifestyles and urban forms/built environment in these countries were completely different. The demographic background of the participants was also different. The study from Cameroon was focused on children. Therefore, the positive association between LTPA and CMSP found in Cameroon could be due to participation in competitive sports in children, which could be associated with recurrent overuse injuries.^67^ It would be unreasonable to generalize the results of the Cameroonian study with the negative LTPA-CMSP association found in South African adults. The regions where within-region agreement was found could be a geographical cluster, e.g., all included studies involved the same country in the region. The studies from South America showed homogeneous results from 10 included studies. They all came from Brazil, 7 of them in particular from the south of Brazil. On the one hand, these 10 studies covered both genders and a wide range of ages and occupations, which suggests the existence of an influence of inherited genetic factors.^68^ On the other hand, the representativeness of these Brazilian studies for the whole of South America was questionable. Furthermore, in addition to South America, positive deviations from overall OR were also found in Central Europe and sub-Saharan Africa, while South Asia and the Middle East showed negative deviations. Central Europe, South Asia and the Middle East suffered from small sample sizes (n ≤ 6) and obvious outliers. Confirmation of their deviations would require additional sampling.

The underlying mechanism of the association between LTPA and CMSP needs to be further investigated by path analysis, as we found that the high degree of heterogeneity in the overall meta-analysis persisted in most subgroups of the subgroup analyses. For example, the only age groups with significant results and narrow confidence intervals were adults and middle-aged and older adults. From a geographical perspective, regions with a higher proportion of high-income countries, such as Northern Europe and North America, had narrower confidence intervals than regions with a higher proportion of low/middle-income countries, such as Southeast Asia and sub-Saharan Africa.

In addition, future studies should collect longitudinal data using validated questionnaires to assess LTPA^69^, ^70^ and CMSP^71^ to evaluate the local burden of disease to improve clarification of the association between LTPA and CMSP. In particular, more sociodemographic and lifestyle information as well as comorbidities (e.g. age, education, socioeconomic status, body mass index, smoking and alcohol consumption, anxiety and depression, fear avoidance) would be useful to investigate the underlying factors influencing the association between LTPA and CMSP.^17^, ^72^ In addition, most studies from under-sampled regions such as Central America, Eastern Europe, North Africa and North Asia are needed to identify the causes of significant geographic variation, including sample size bias and variability in health/social system, cultural practice/lifestyle and urban form/built environment.

Similar to previous reviews, this study excluded studies with non-English full texts. However, 62% of the included studies (*n* = 74) were conducted in non-English speaking countries from almost all geographical regions, so our review is still applicable to describe the global difference. Future studies may include studies in other languages if the publications are of high quality to improve the causal results. Despite strict inclusion/exclusion criteria, this study included some studies in high-income countries that objectively measured the level of LTPA of children and adolescents at school using continuous, day-long accelerometers. This would include occupational physical activity due to illegal child labor, but the risk of bias is low as the prevalence of child labor in these countries is only 0.9%.^73^ On the other hand, LTPA frequency was extracted but not used as a factor in the analysis. An investigation of a possible dose-response relationship would be very valuable and applicable in practice. However, the dose-response relationship, if any, could be significantly modified by the duration and intensity of individual training sessions. Such data were not reported in the included studies. The risk of bias assessment identified a significant proportion of included studies with a high risk of overall bias, which could be due to a lack of communication with the authors of the included studies during the review process.^74^ On general inspection, the statistical results from these studies were not significantly different from those from studies with a lower risk of bias. The presence of moderator variables leading to high heterogeneity could contribute to the residual risk of publication bias.^75^

## Conclusions

Our results suggest that the benefits of LTPA cannot be considered universally applicable. A negative overall association between LTPA and CMSP could potentially be influenced by several underlying factors. Apart from the significant findings in middle-aged and older adults, the negative association between LTPA and CMSP observed in all pain sites and the differences between geographic locations should be further investigated. This can help explore the underlying mechanisms influencing the association comprehensively and effectively between LTPA and CMSP in all genders and different age groups. Locally and regionally tailored interventions should be developed that take into account the structural, economic and social characteristics of each location.

## Ethics approval and consent to participate

Not Applicable. This study did not involve human participants and informed consent was therefore not required.

## Funding

This study was partially support by the funding from the Peter Hung Professorship in Pain Research, H H Hung Charitable Foundation, and 10.13039/501100005847Health and Medical Research Fund (Project Code: 21221091).

## CRediT authorship contribution statement

**Chi Wai Cheung:** Writing – review & editing, Validation, Supervision, Resources, Project administration, Methodology, Investigation, Funding acquisition, Conceptualization. **Fengfeng Wang:** Writing – review & editing, Validation, Formal analysis. **Lin Wang:** Writing – review & editing, Validation, Formal analysis. **Hung Chak Ho:** Writing – review & editing, Validation, Supervision, Project administration, Methodology, Conceptualization. **Elmo Wing-Yiu Lee:** Writing – review & editing, Writing – original draft, Visualization, Validation, Methodology, Investigation, Formal analysis, Data curation, Conceptualization.

## Declaration of Competing Interest

No completing interests

## Data Availability

Data that supports the findings of this study are available in the supplementary material of this article.

## Appendix A Supplementary material

Supplementary material

Supplementary material Table 1: Search keywords

Supplementary material Table 2: Extracted data for 137 studies included in systematic review
