# Supplementary material for: Global disparities in association between leisure-time physical activity and chronic musculoskeletal pain: A systematic review and meta-analysis
Source: Glob Health Res Policy. 2026 May 20;11(1):74–83. doi: 10.1016/j.ghrp.2026.05.002 (PMC13273654; doi:10.1016/j.ghrp.2026.05.002)

**Supp. Figure 1:** Forest plot – subgroup analysis by gender. A pooled odds ratio with 95% confidence interval was calculated by the random-effects model modified by Knapp and Hartung for each subgroup. The difference between the pooled statistics from each subgroup was evaluated by a Wald-type test. Mixed-sex subgroup referred to data calculated from population samples consisting of both men and women. CI, confidence interval.


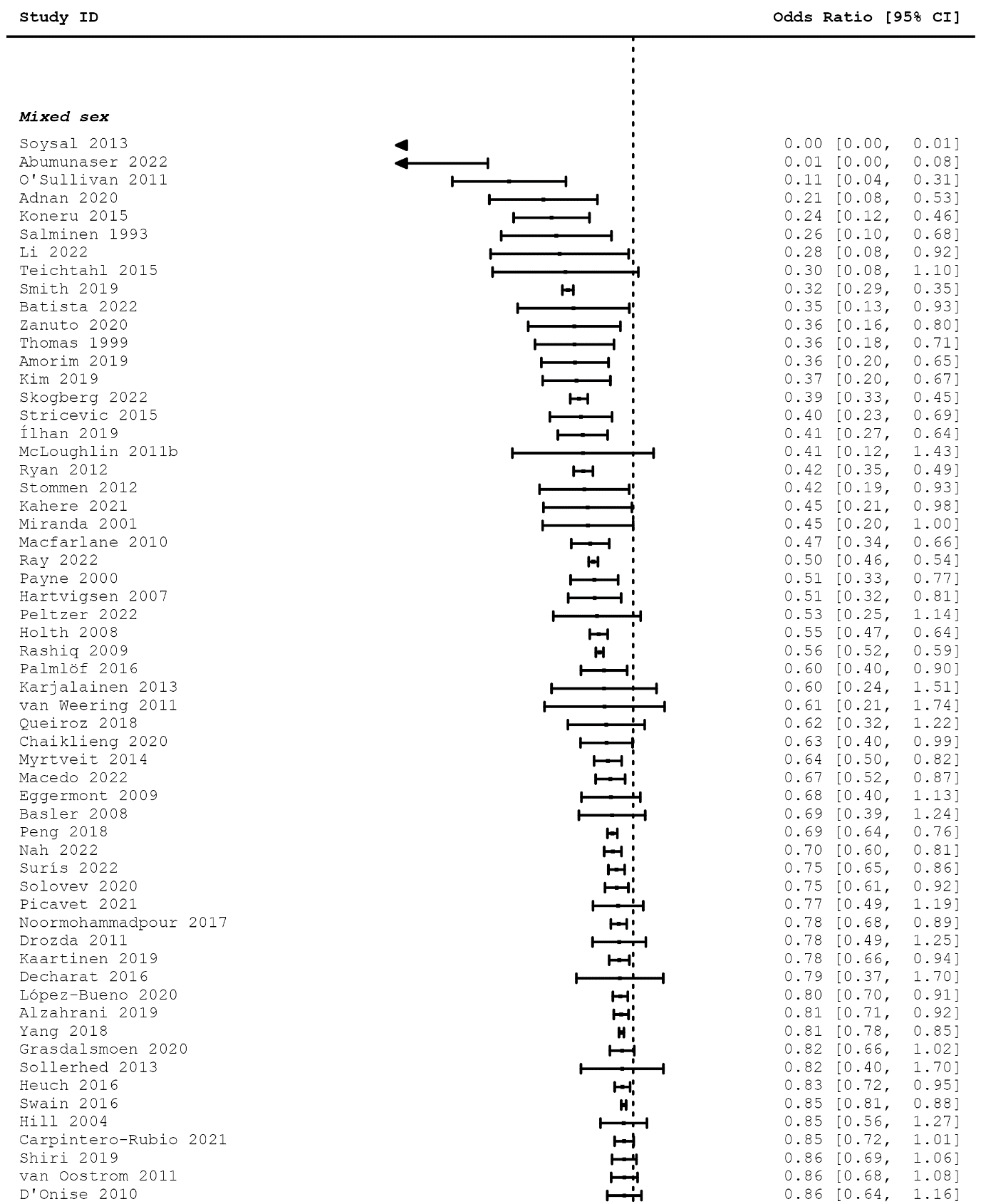


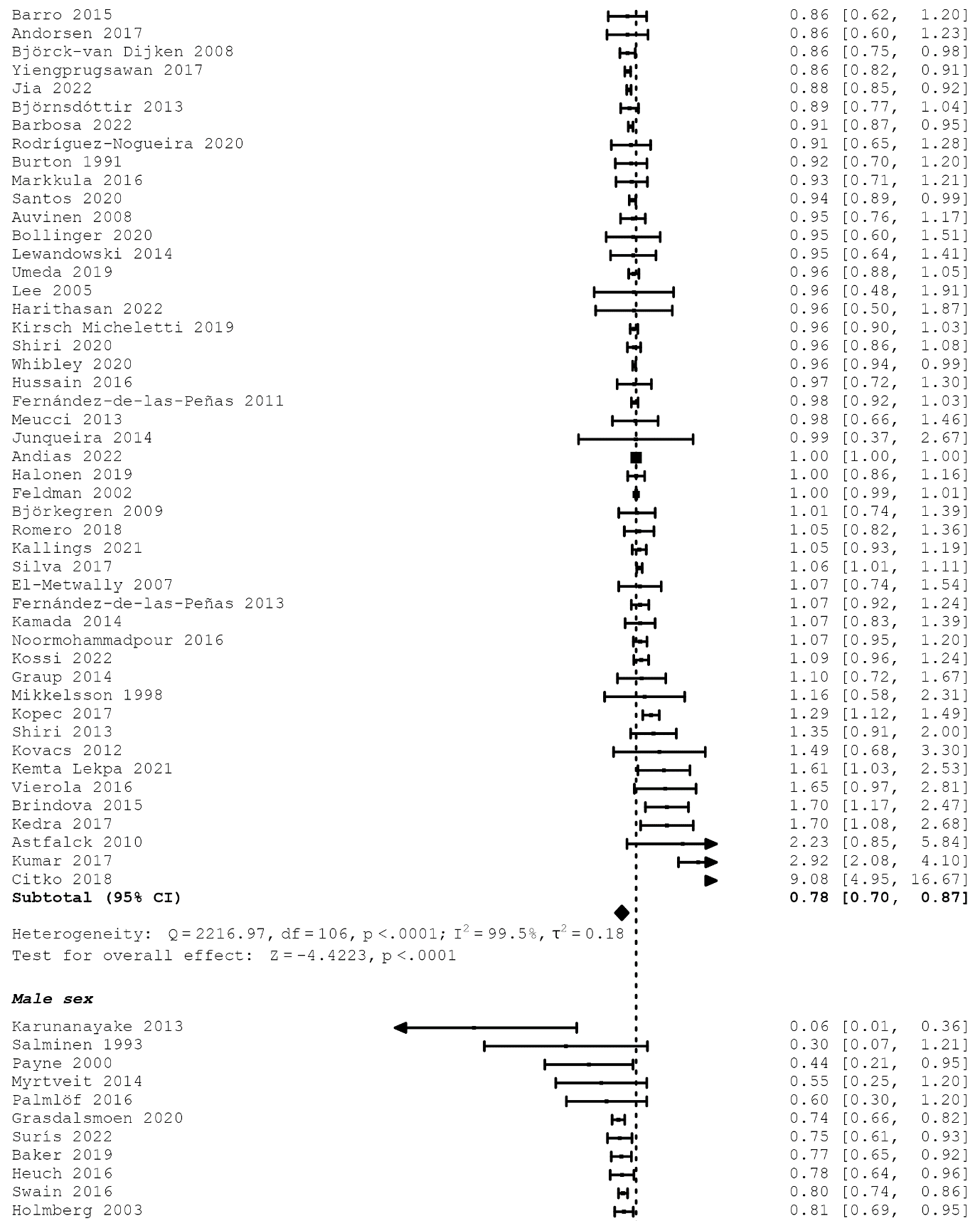


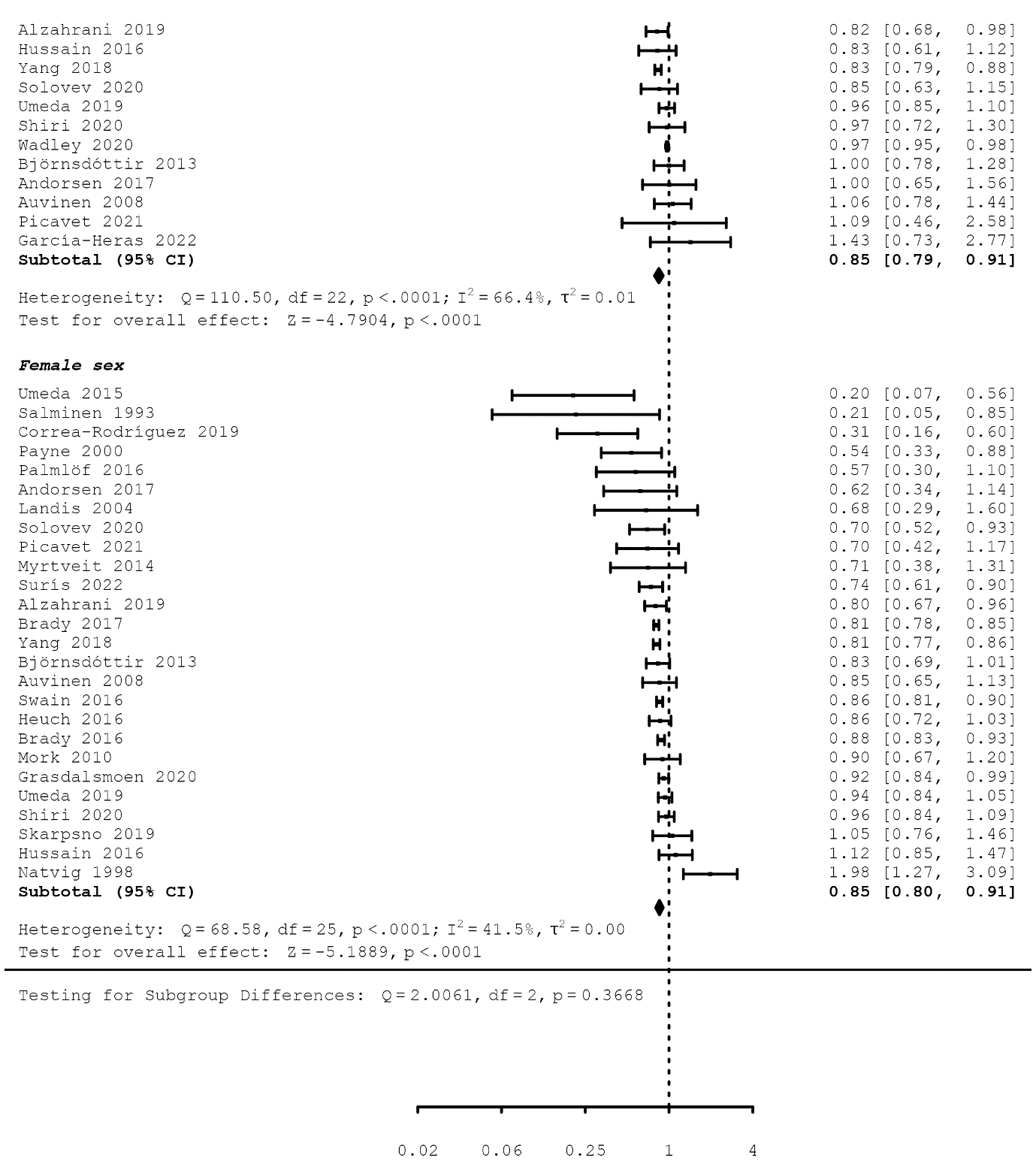


**Supp. Figure 2:** Forest plot – subgroup analysis by age groups. A pooled odds ratio with 95% confidence interval was calculated by the random-effects model modified by Knapp and Hartung for each subgroup. The difference between the pooled statistics from each subgroup was evaluated by a Wald-type test. Children, age ≤ 13 years; adolescents, 10 years ≤ age < 18 years; adults, age ≥ 18 years; younger adults, 18 years ≤ age ≤ 28 years; older adults, age ≥ 40 years. CI, confidence interval.


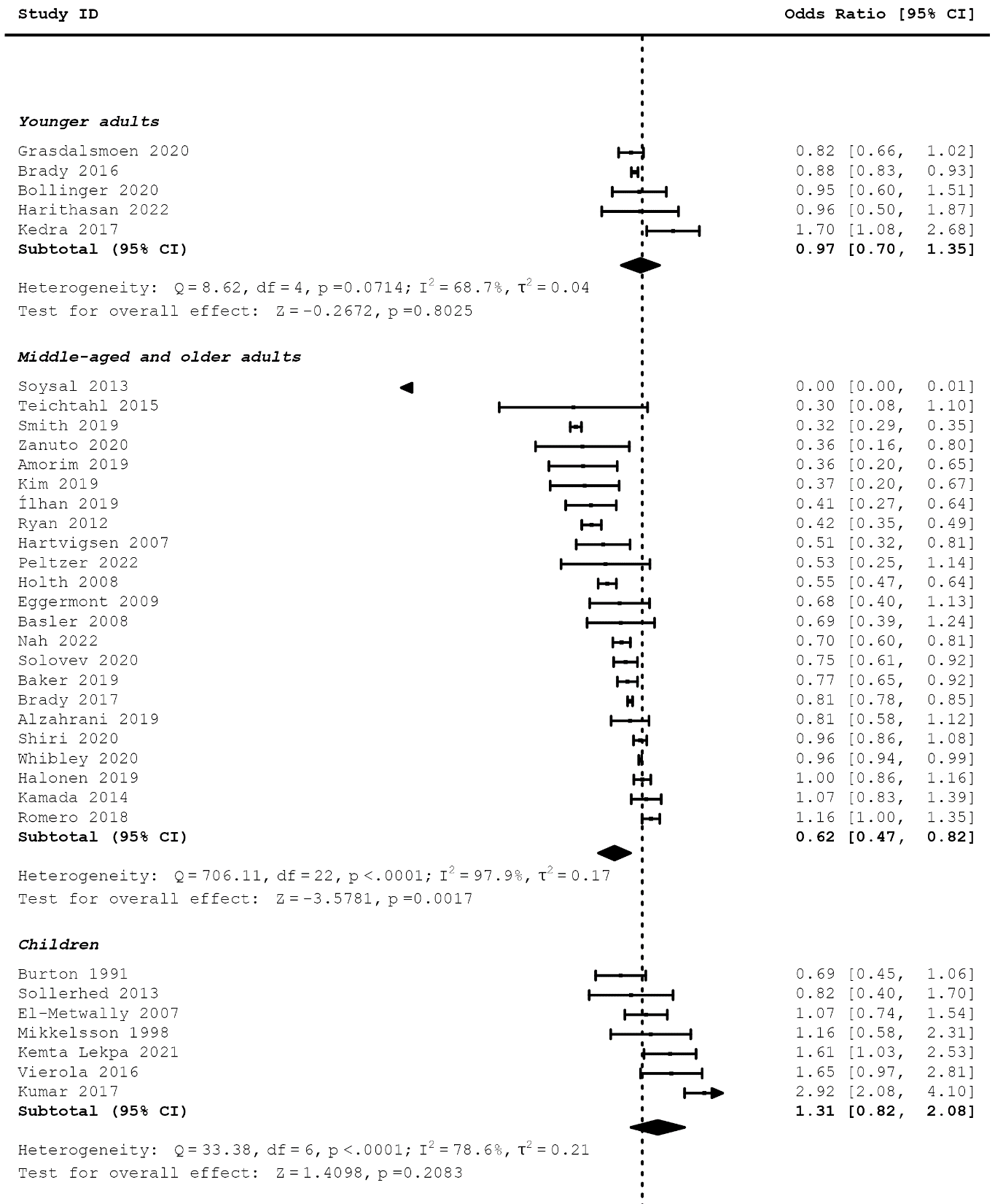


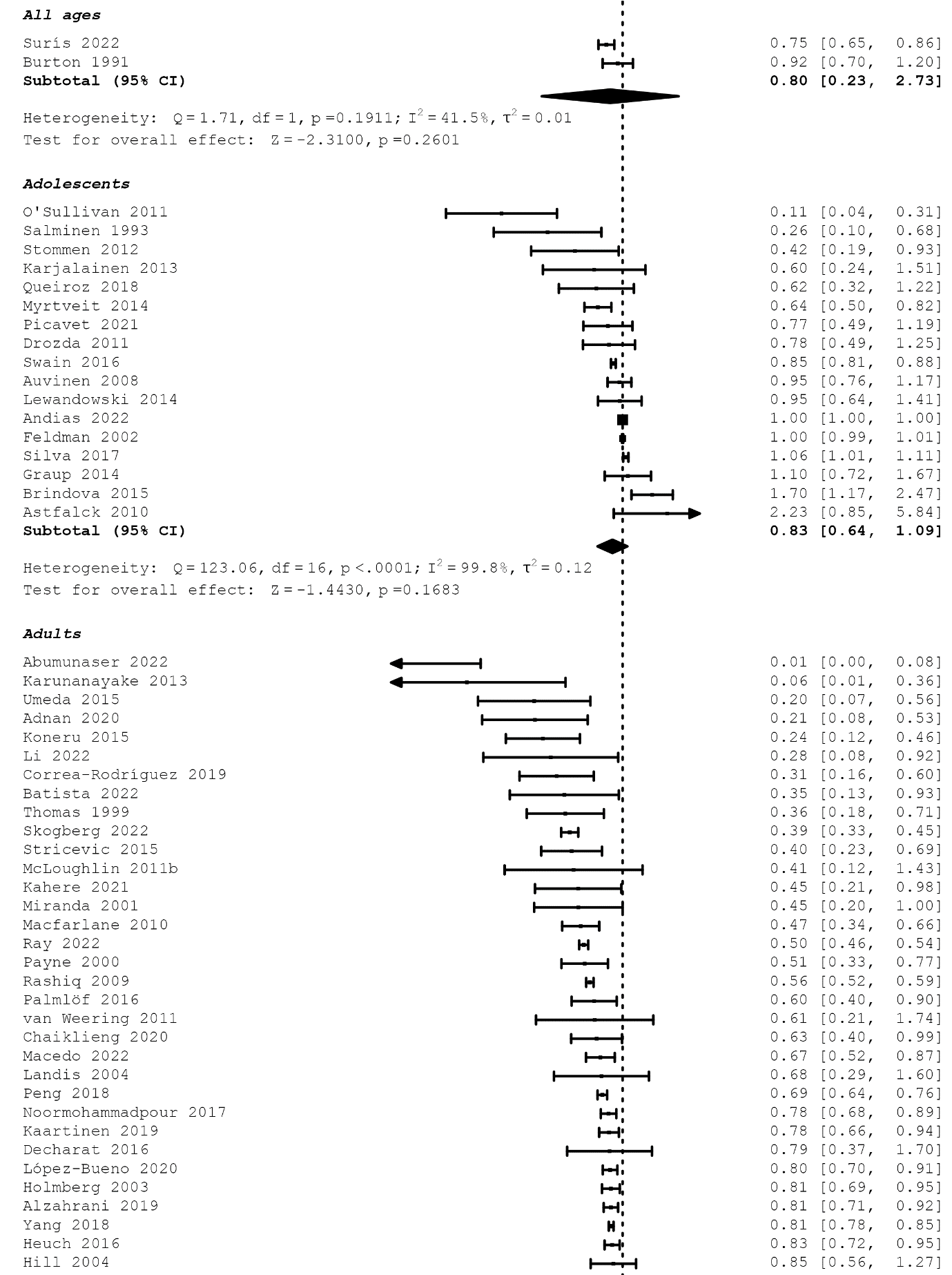


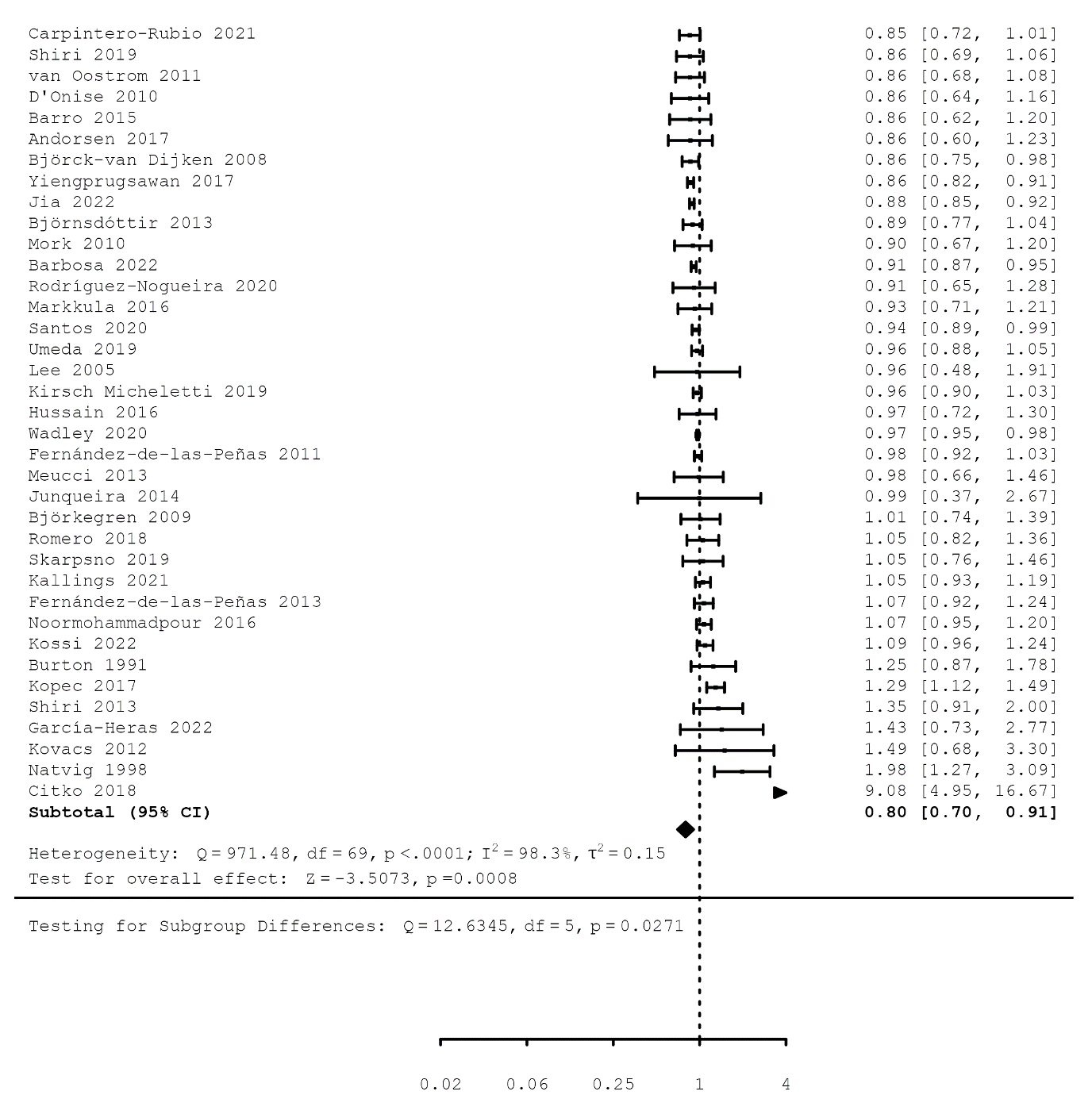


**Supp. Figure 3:** Forest plot – subgroup analysis by location of CMSP. A pooled odds ratio with 95% confidence interval was calculated by the random-effects model modified by Knapp and Hartung for each subgroup. The difference between the pooled statistics from each subgroup was evaluated by a Wald-type test. CI, confidence interval.

**
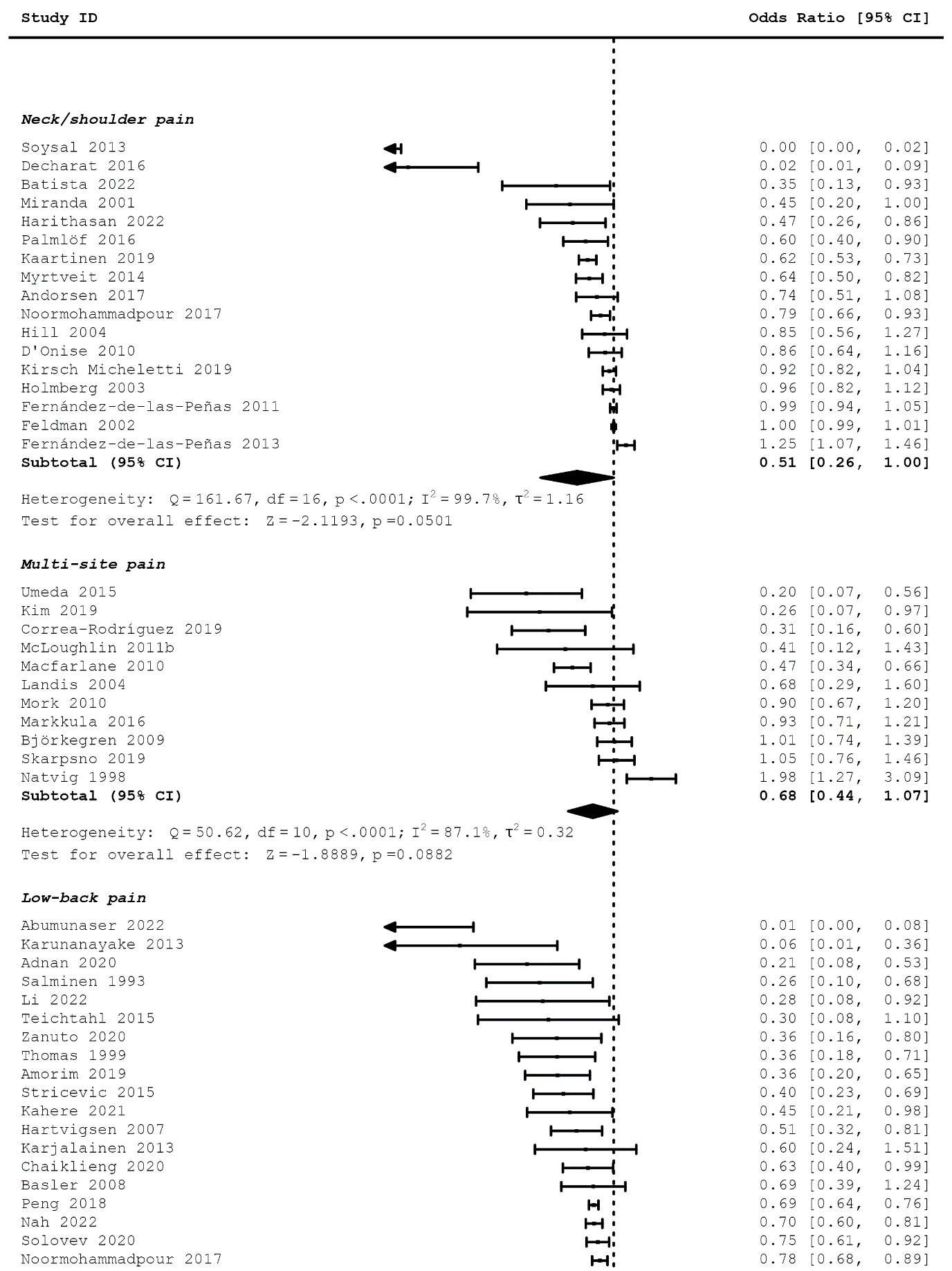
**

**
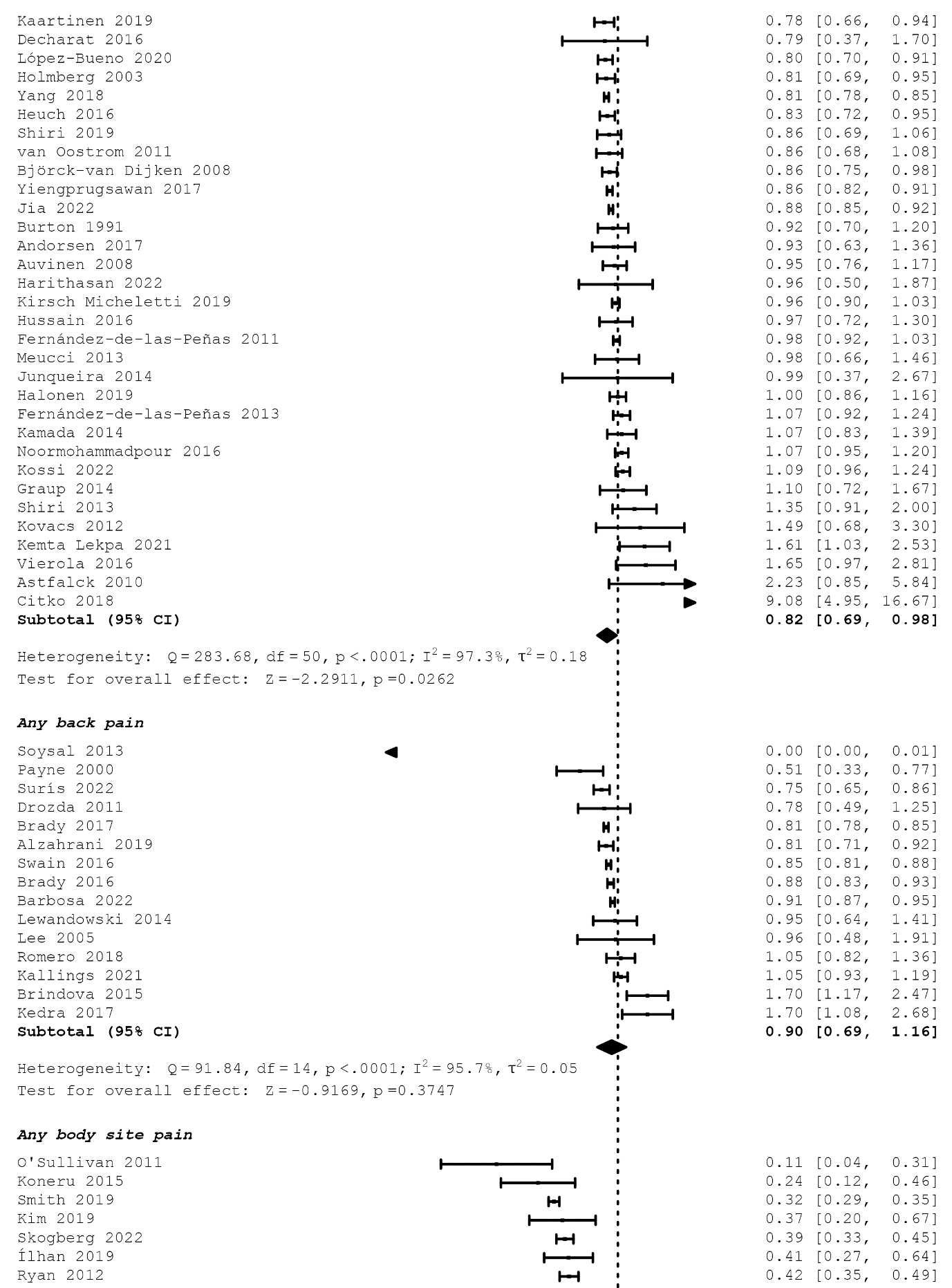
**

**
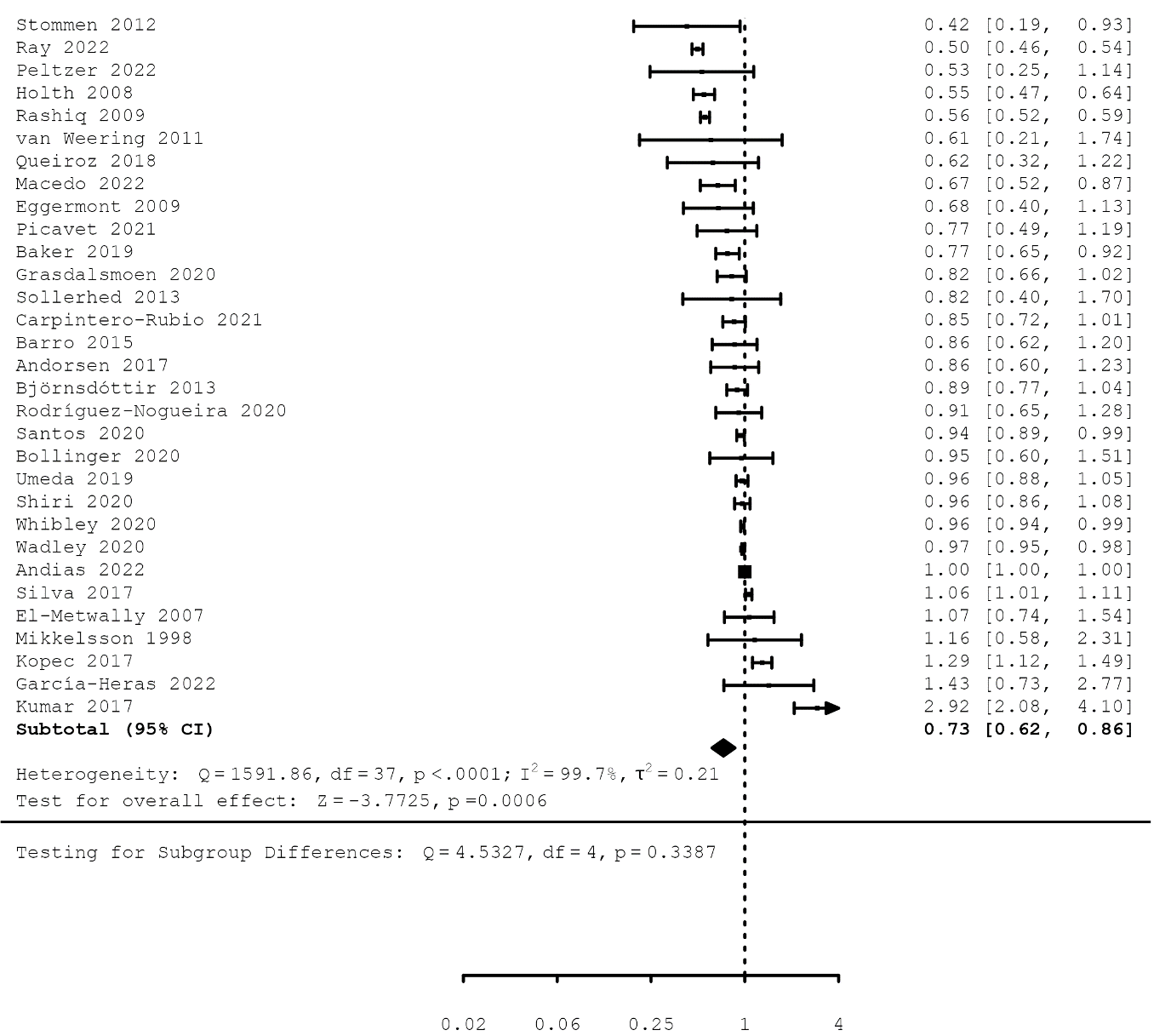
**

**Supp. Figure 4:** Forest plot – subgroup analysis by country income level. A pooled odds ratio with 95% confidence interval was calculated by the random-effects model modified by Knapp and Hartung for each subgroup. The difference between the pooled statistics from each subgroup was evaluated by a Wald-type test. CI, confidence interval.


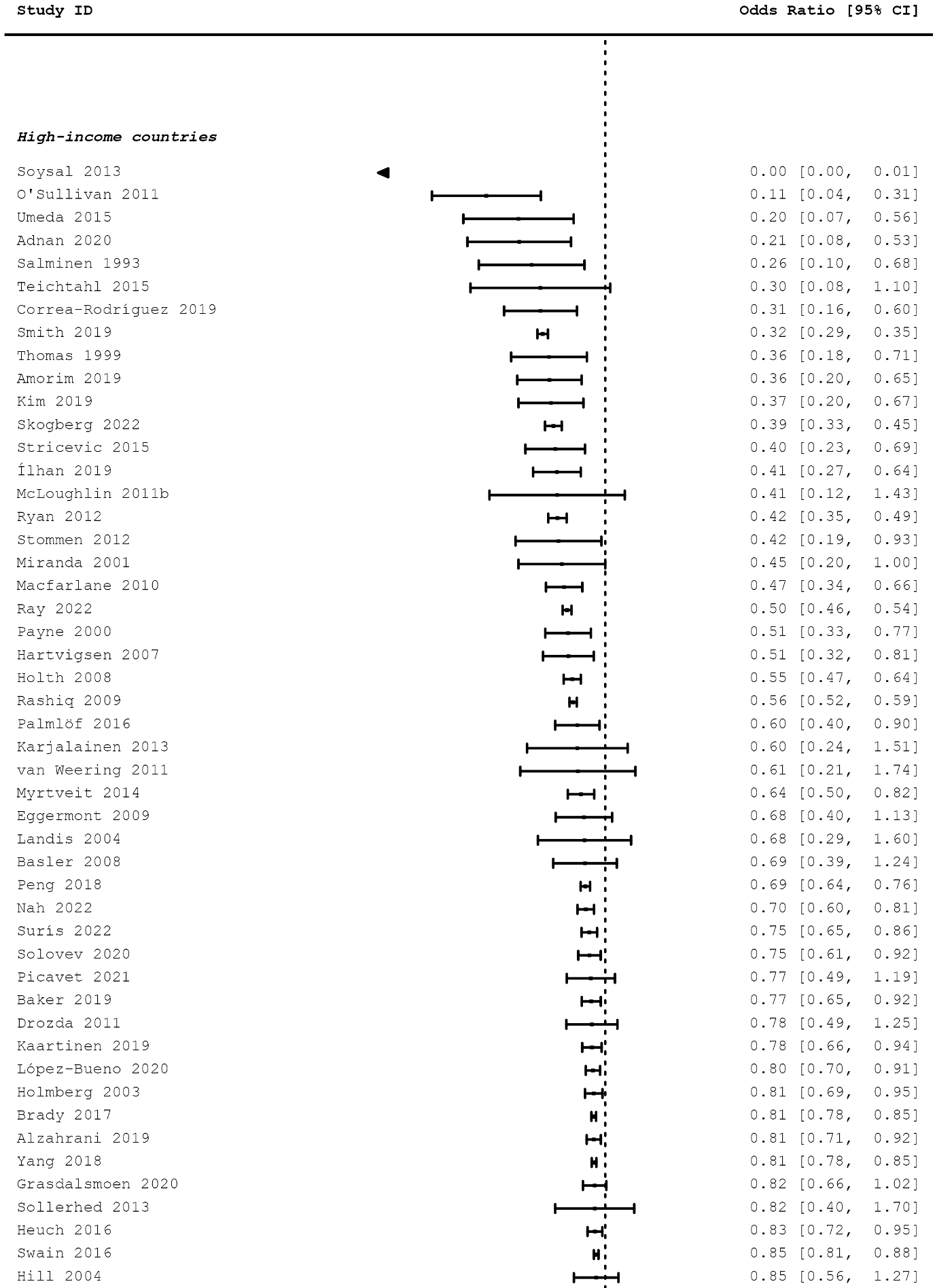


***
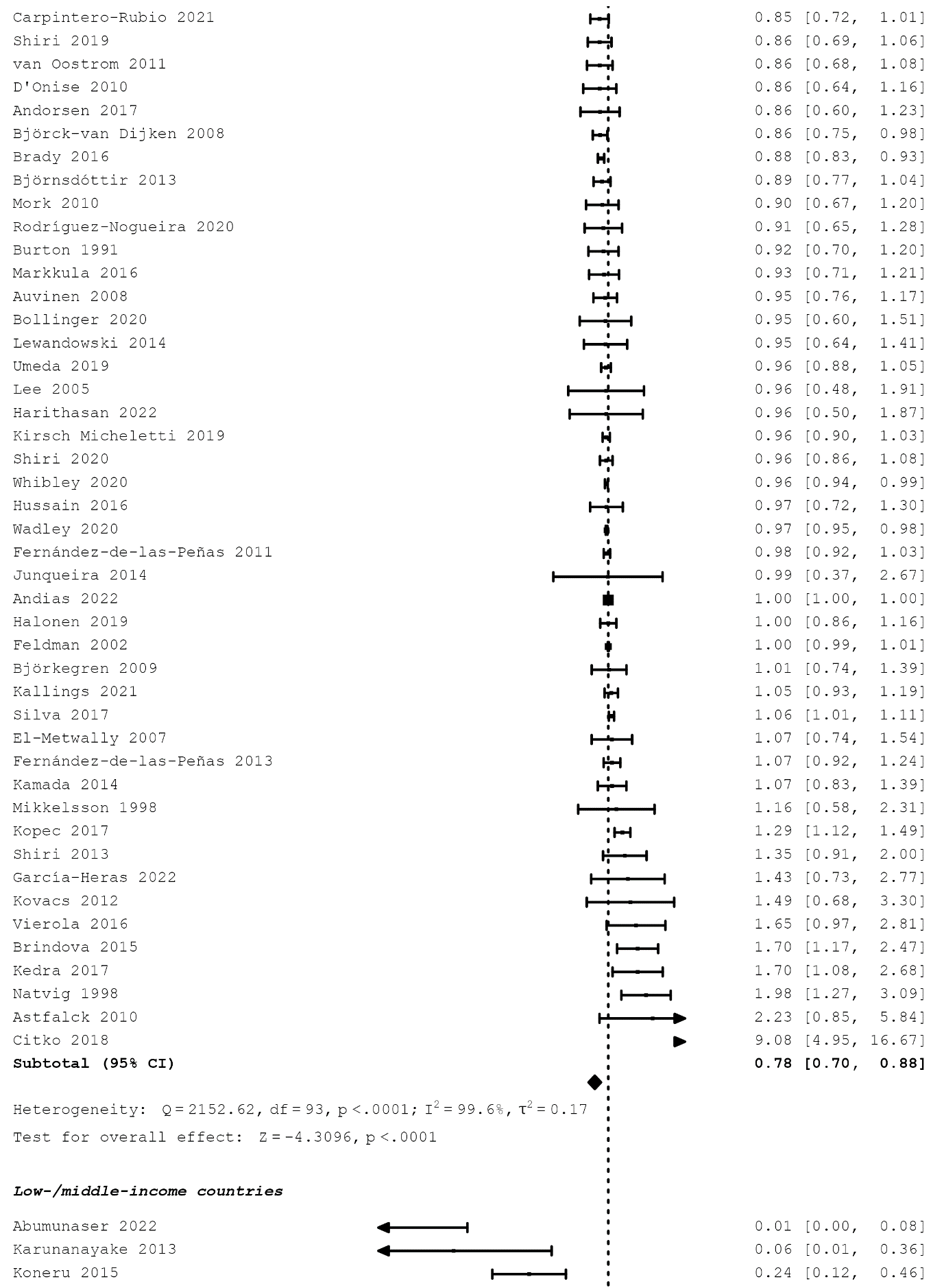
***

***
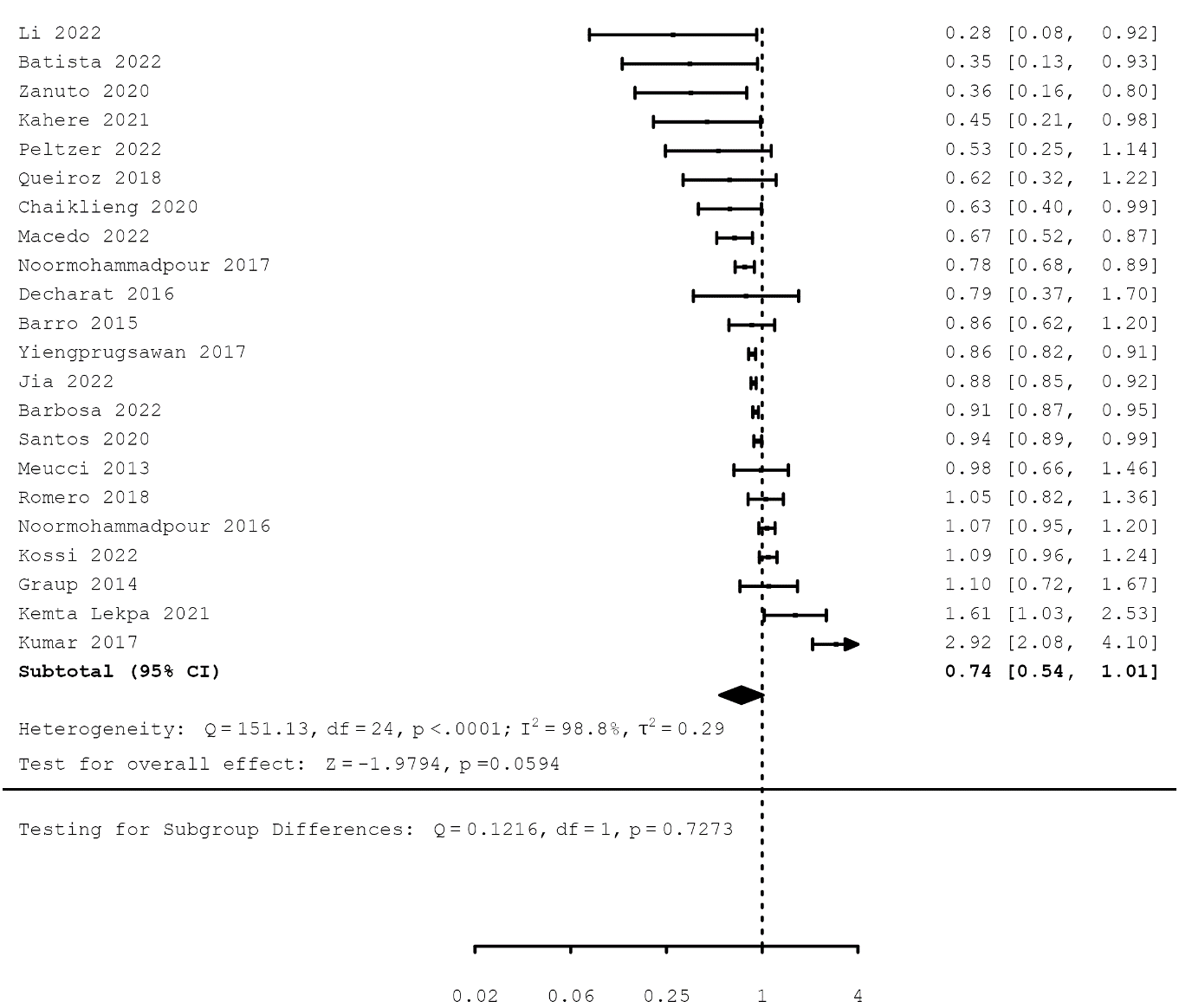
***

**Supp. Figure 5:** Forest plot – subgroup analysis by geographical continent. A pooled odds ratio with 95% confidence interval was calculated by the random-effects model modified by Knapp and Hartung for each subgroup. The difference between the pooled statistics from each subgroup was evaluated by a Wald-type test. CI, confidence interval.


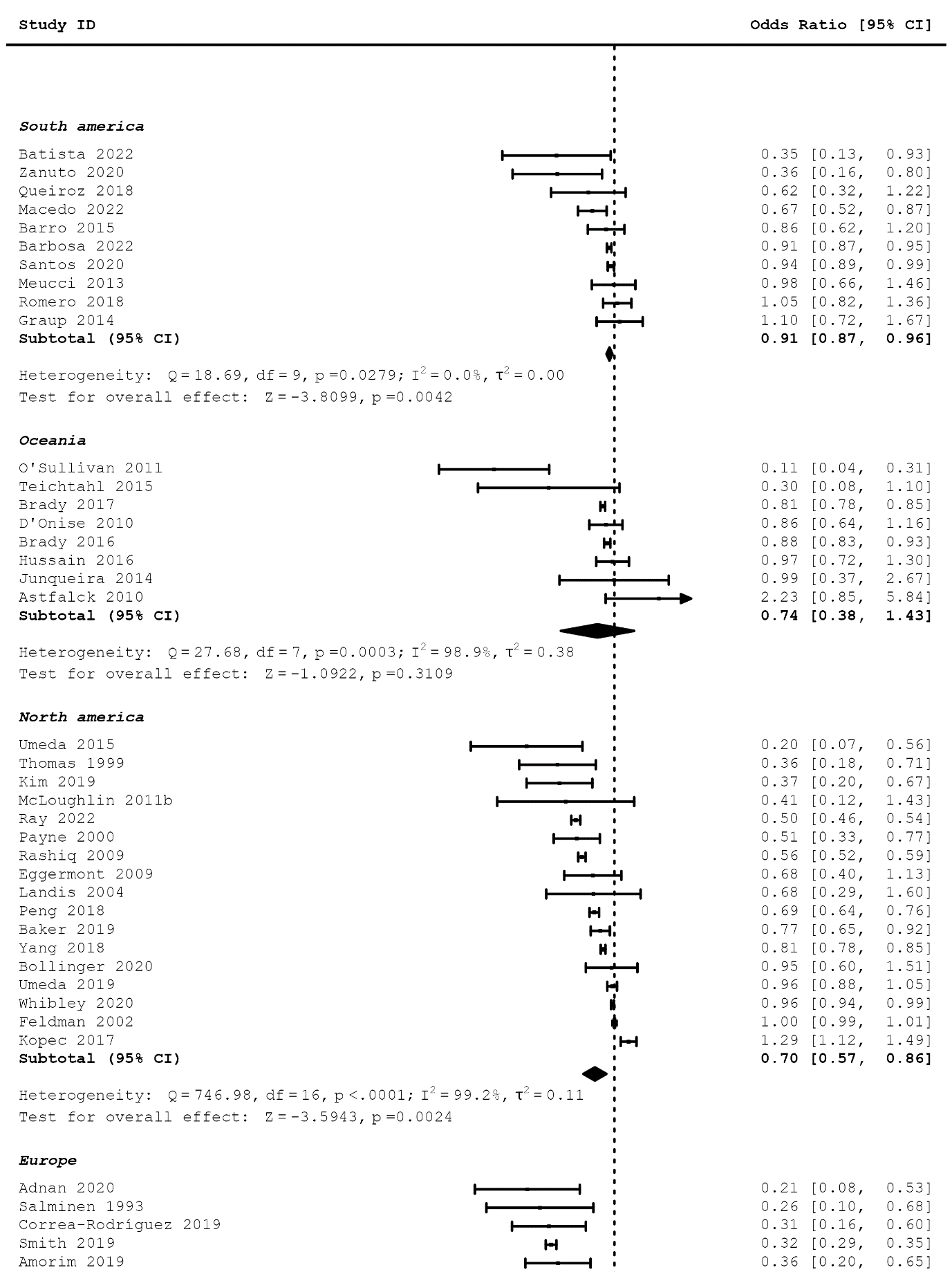


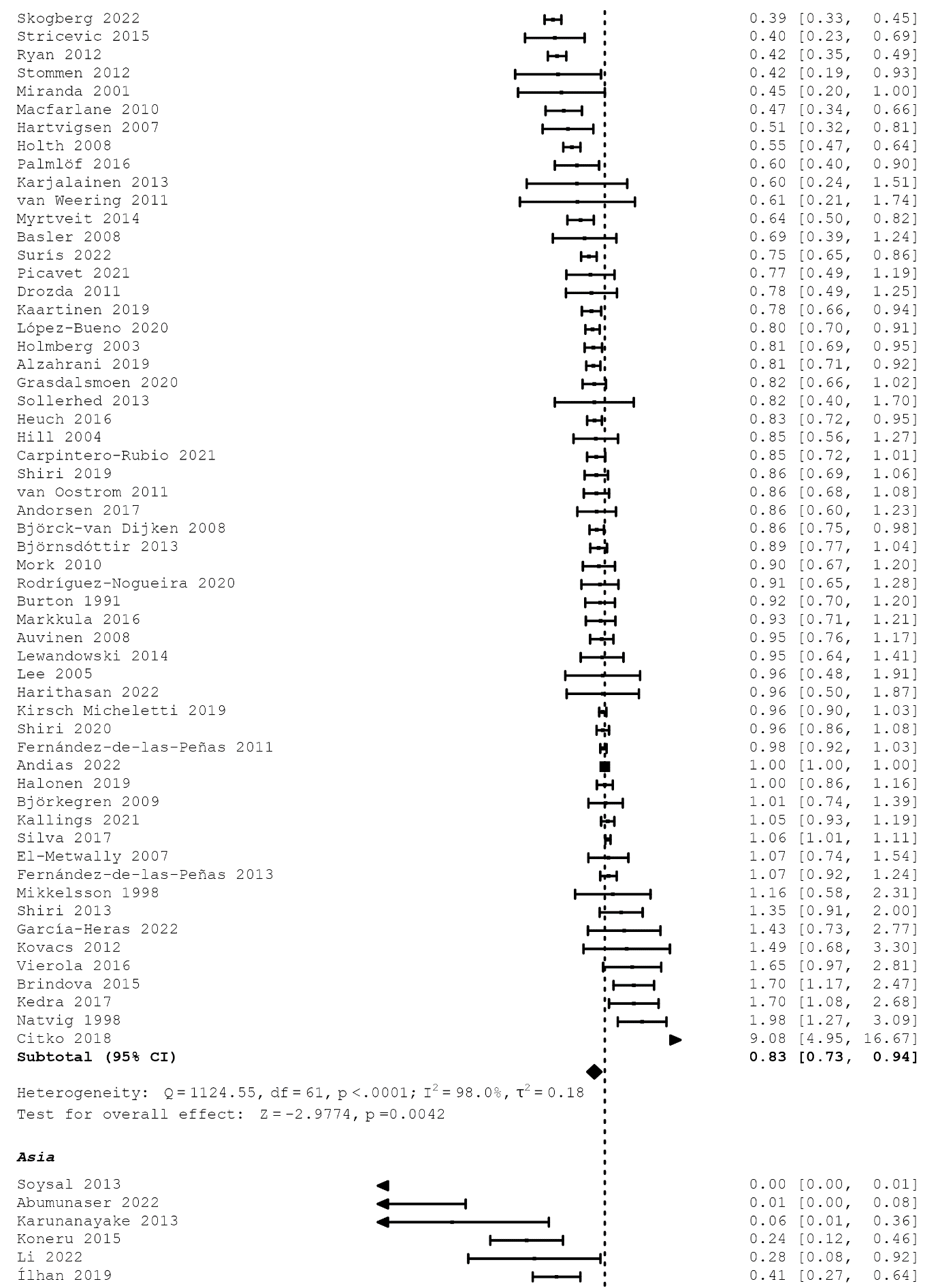


**
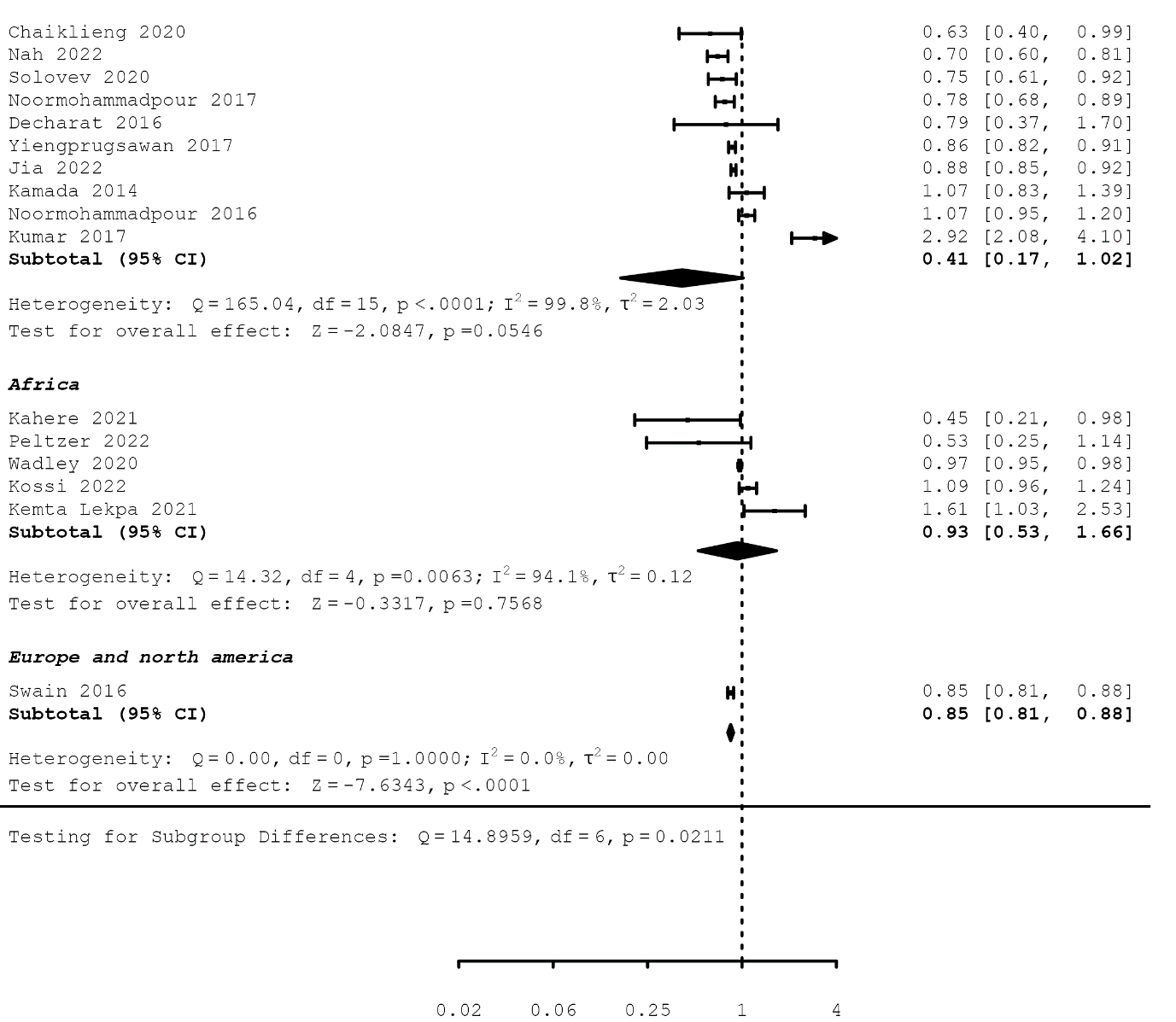
**

**Supp. Figure 6:** Forest plot – subgroup analysis by geographical region. A pooled odds ratio with 95% confidence interval was calculated by the random-effects model modified by Knapp and Hartung for each subgroup. The difference between the pooled statistics from each subgroup was evaluated by a Wald-type test. CI, confidence interval.


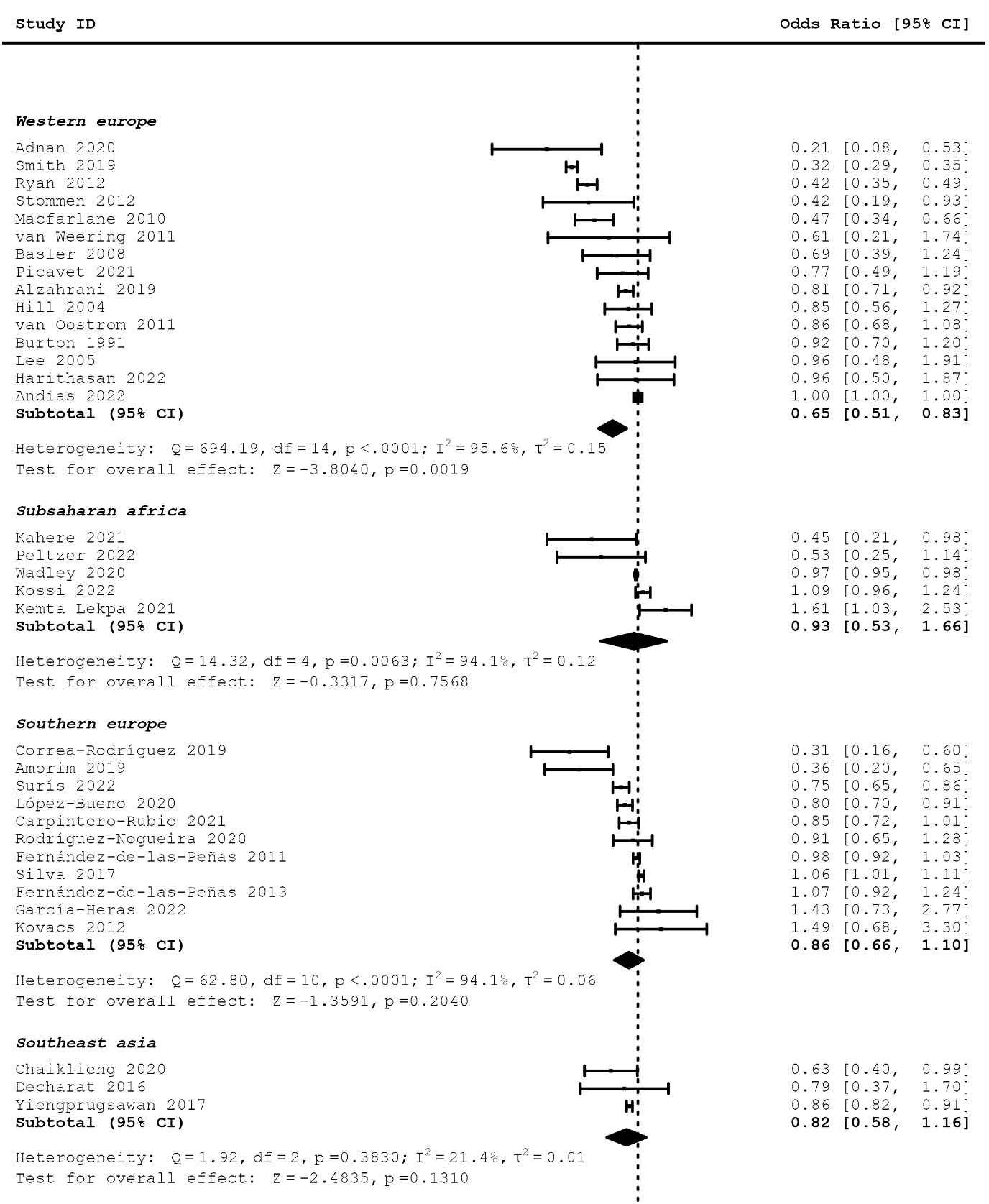


***
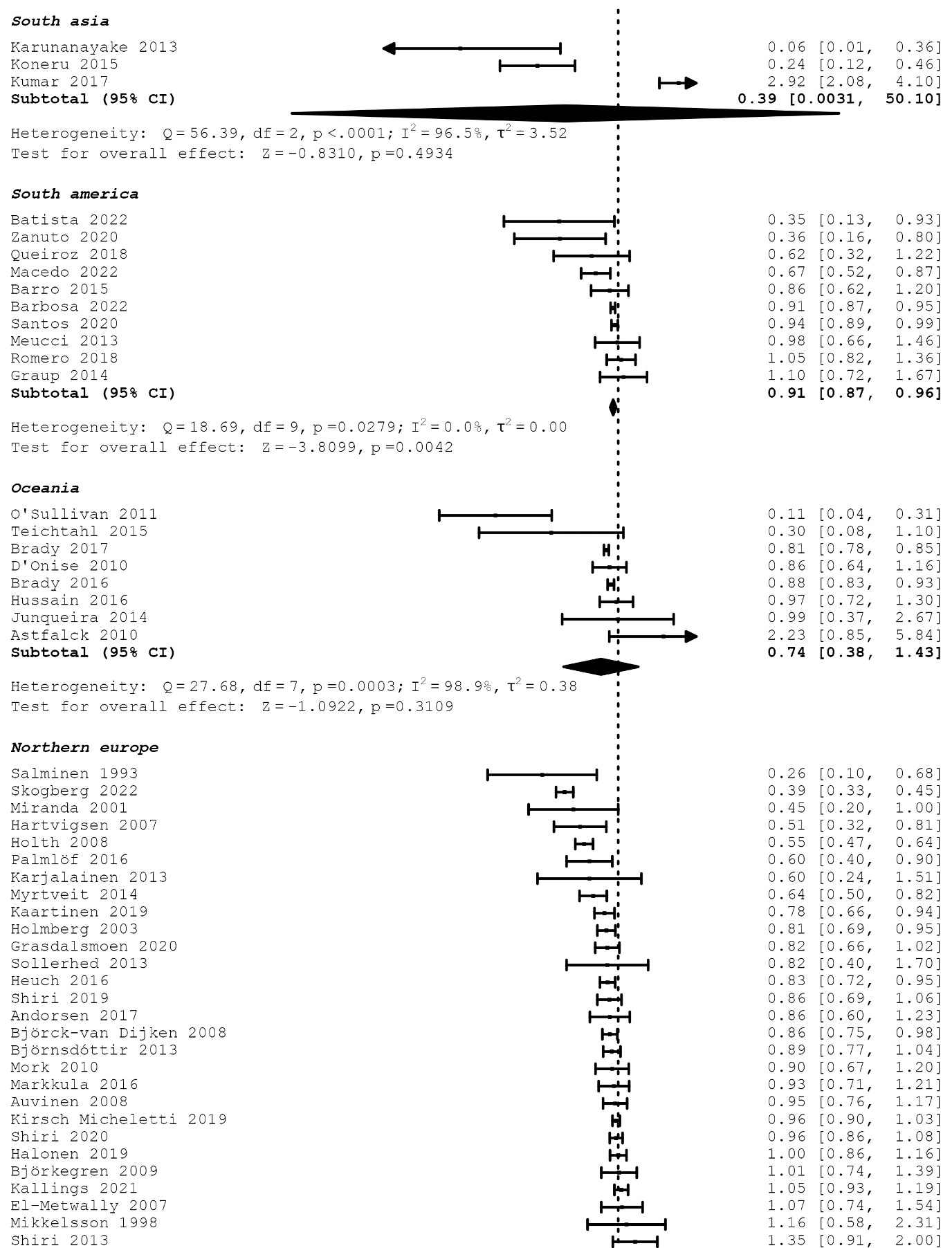
***


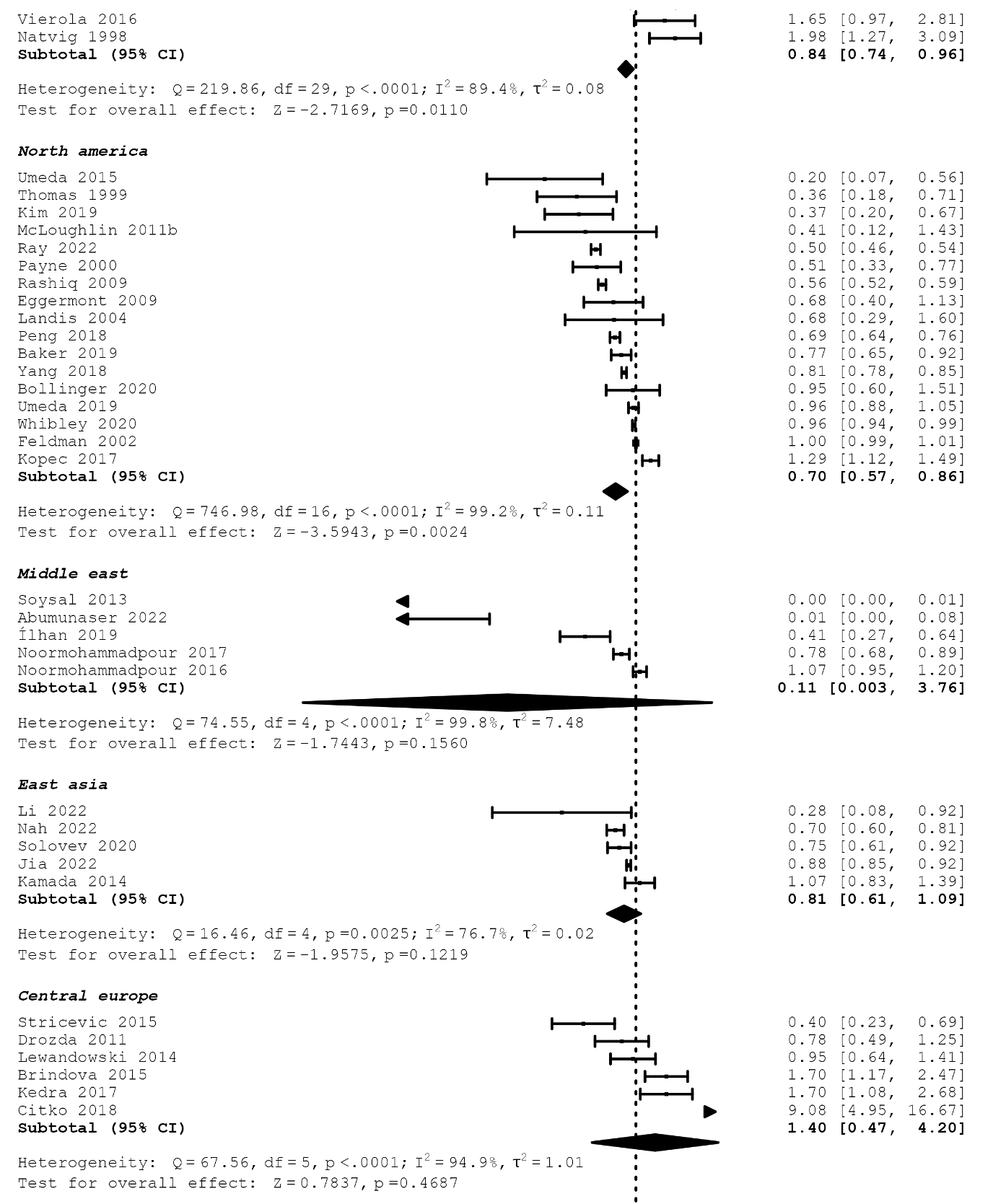


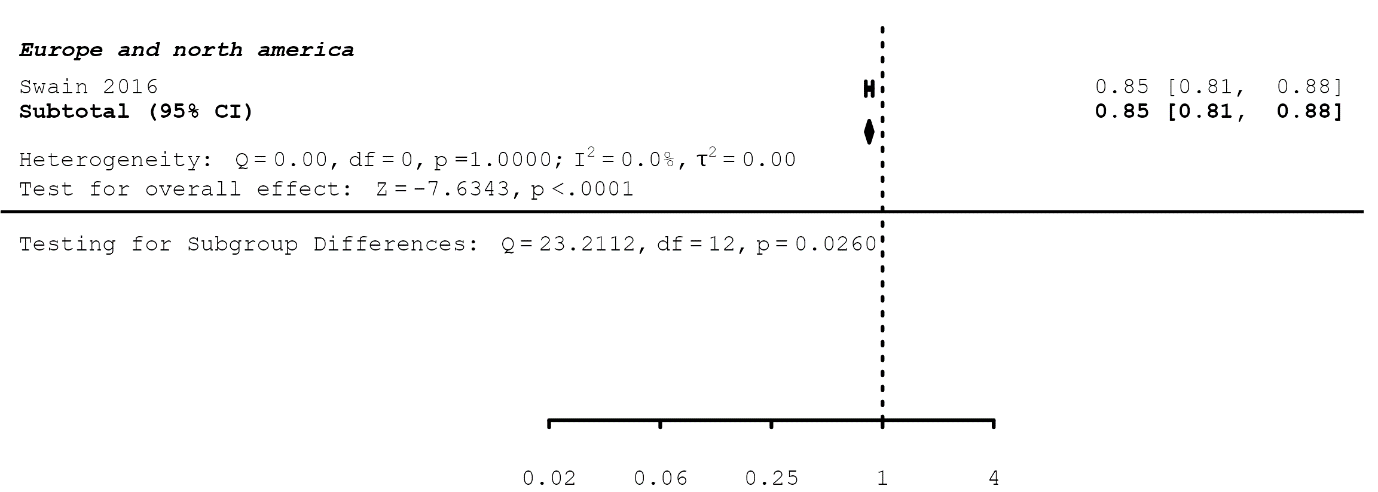

Supplement: Supplementary file 1 — Supplementary material [file mmc1.docx]
