# Supplementary material for: Global disparities in association between leisure-time physical activity and chronic musculoskeletal pain: A systematic review and meta-analysis
Source: Glob Health Res Policy. 2026 May 20;11(1):74–83. doi: 10.1016/j.ghrp.2026.05.002 (PMC13273654; doi:10.1016/j.ghrp.2026.05.002)
Supplement: Supplementary file 2 — Supplementary material Table 1: Search keywords [file mmc2.docx]

# PubMed

(“pain”[mh] OR “pain”[tiab] OR “chronic pain”[mh] OR “chronic pain”[tiab] OR “musculoskeletal pain”[mh] OR “musculoskeletal pain”[tiab] OR “musculoskeletal symptoms”[tiab] OR “fibromyalgia”[mh] OR “fibromyalgia”[tiab]) AND (“exercise”[mh] OR “exercis*”[tiab] OR “physical activit*”[tiab] OR “physical inactivity”[tiab] OR

“sedentary behavior”[mh] OR “sedentary behavior”[tiab] OR “sedentary lifestyle”[tiab] OR

“walking”[tiab] OR “swimming”[tiab] OR “running”[tiab] OR “jogging”[tiab] OR

“swimming”[tiab] OR “cycling”[tiab] OR “martial arts”[tiab] OR “yoga”[tiab]) NOT (“Pain,

Postoperative”[mh] OR “surgery”[tiab] OR “Surgical Procedures, Operative”[mh] OR

“neoplasms”[mh] OR “cancer”[tiab] OR “therapy”[mh] OR “therap*”[tiab] OR “treat*”[tiab] OR “manag*”[tiab] OR “interven*”[tiab] OR “trial”[tiab] OR “trials”[tiab] OR “acute pain”[mh] OR “acute”[tiab] OR “arthritis”[mh] OR “arthritis”[tiab] OR “osteoarthritis”[mh]

OR “osteoarthritis”[tiab] OR “rodentia”[mh] OR “rodent”[tiab] OR “mice”[mh] OR

“mouse”[tiab] OR “mice”[tiab] OR “rats”[mh] OR “rat”[tiab] OR “review”[ti] OR "disease"[ti] OR "disorder"[ti] OR “syndrome”[ti] OR "cardiovascular diseases"[mh])

# Embase and MEDLINE via Ovid

(exp "pain"/ or "pain".ti,ab,cl,oa,kw,kf. or exp "chronic pain"/ or "chronic pain".ti,ab,cl,oa,kw,kf. or exp "musculoskeletal pain"/ or "musculoskeletal pain".ti,ab,cl,oa,kw,kf. or "musculoskeletal symptoms".ti,ab,cl,oa,kw,kf. or exp "fibromyalgia"/ or "fibromyalgia".ti,ab,cl,oa,kw,kf.) and (exp "exercise"/ or "exercis*".ti,ab,cl,oa,kw,kf. or "physical activit*".ti,ab,cl,oa,kw,kf. or "physical inactivity".ti,ab,cl,oa,kw,kf. or exp "sedentary behavior"/ or "sedentary behavior".ti,ab,cl,oa,kw,kf. or "sedentary lifestyle".ti,ab,cl,oa,kw,kf. or

"walking".ti,ab,cl,oa,kw,kf. or "swimming".ti,ab,cl,oa,kw,kf. or "running".ti,ab,cl,oa,kw,kf. or "jogging".ti,ab,cl,oa,kw,kf. or "swimming".ti,ab,cl,oa,kw,kf. or "cycling".ti,ab,cl,oa,kw,kf. or "martial arts".ti,ab,cl,oa,kw,kf. or "yoga".ti,ab,cl,oa,kw,kf.) not (exp "pain, postoperative"/ or "surgery".ti,ab,cl,oa,kw,kf. or exp "surgical procedures, operative"/ or exp "neoplasms"/ or

"cancer".ti,ab,cl,oa,kw,kf. or exp "therapy"/ or "therap*".ti,ab,cl,oa,kw,kf. or

"treat*".ti,ab,cl,oa,kw,kf. or "manag*".ti,ab,cl,oa,kw,kf. or "interven*".ti,ab,cl,oa,kw,kf. or

"trial".ti,ab,cl,oa,kw,kf. or "trials".ti,ab,cl,oa,kw,kf. or exp "acute pain"/ or

"acute".ti,ab,cl,oa,kw,kf. or exp "arthritis"/ or "arthritis".ti,ab,cl,oa,kw,kf. or exp

"osteoarthritis"/ or "osteoarthritis".ti,ab,cl,oa,kw,kf. or exp "rodentia"/ or

"rodent".ti,ab,cl,oa,kw,kf. or exp "mice"/ or "mouse".ti,ab,cl,oa,kw,kf. or

"mice".ti,ab,cl,oa,kw,kf. or exp "rats"/ or "rat".ti,ab,cl,oa,kw,kf. or "review".ti. or "disease".ti.

or "disorder".ti. or "syndrome".ti. or exp "cardiovascular diseases"/)

# Web of Science

TS=((pain OR pain OR "chronic pain" OR "chronic pain" OR "musculoskeletal pain" OR

"musculoskeletal pain" OR "musculoskeletal symptoms" OR fibromyalgia OR fibromyalgia) AND (exercise OR exercis* OR "physical activit*" OR "physical inactivity" OR "sedentary behavior" OR "sedentary behavior" OR "sedentary lifestyle" OR walking OR swimming OR running OR jogging OR swimming OR cycling OR "martial arts" OR yoga) NOT ("Pain, Postoperative" OR surgery OR "Surgical Procedures, Operative" OR neoplasms OR cancer OR therapy OR therap* OR treat* OR manag* OR interven* OR trial OR trials OR "acute pain" OR acute OR arthritis OR arthritis OR osteoarthritis OR osteoarthritis OR rodentia OR rodent OR mice OR mouse OR mice OR rats OR rat OR review OR disease OR disorder OR syndrome OR "cardiovascular diseases"))
