# Supplementary material for: Global disparities in association between leisure-time physical activity and chronic musculoskeletal pain: A systematic review and meta-analysis
Source: Glob Health Res Policy. 2026 May 20;11(1):74–83. doi: 10.1016/j.ghrp.2026.05.002 (PMC13273654; doi:10.1016/j.ghrp.2026.05.002)
Supplement: Supplementary file 3 — Supplementary material Table 2: Extracted data for 137 studies included in systematic review [file mmc3.docx]

| **Study ID^@^** | **Study design^@@^** | **Country** | **Age group^**^** | **Occupation** | **LTPA measurement^***^** | **Pain type^^^** | **Pain assessment^^^^** | **N** | **ACR^^^^^** | **Format(s) of other statistical results^#^** | **confounders** | **Groups available^##^** | **Default Group** | **Other groups analysed** | **Default statistics** | **Also analysed groups** | **Other groups statistics** | **Remarks** | **NOS1^###^** | **NOS2^###^** | **NOS3^###^** | **JBI-Q1^###^** | **JBI-Q2^###^** | **JBI-Q3^###^** | **JBI-Q4^###^** | **JBI-Q5^###^** | **JBI-Q6^###^** | **JBI-Q7^###^** | **JBI-Q8^###^** |
| --- | --- | --- | --- | --- | --- | --- | --- | --- | --- | --- | --- | --- | --- | --- | --- | --- | --- | --- | --- | --- | --- | --- | --- | --- | --- | --- | --- | --- | --- |
| Abumunaser 2022 ^1^ | CS | Saudi Arabia | adults | general population | structured questionnaire inquiring weekly PA frequency | LBP | structured questionnaire inquiring prevalence, intensity, medical help-seeking and painkiller use | 288 | 0.678 | Raw counts | N.A. | PA: 6-7/week, 4-5/week, 2-3/week, 1/week, none Back pain (by intensity): none, mild, moderate, severe | >3/week PA -> back pain of at least moderate intensity | >=1/week PA -> back pain of at least moderate intensity >5/week PA -> back pain of at least moderate intensity | 1, 45, 164, 78 | >=1/week PA -> back pain of at least moderate intensity >5/week PA -> back pain of at least moderate intensity | 110, 99, 33, 46 0, 9, 200, 79 | None |  |  |  | T | F | F | N/A | F | F | T | T |
| Adnan 2020 ^2^ | CC | Belgium | adults 36.784 ± 13.13 | general public | Checklist of Individual Strength | LBP | assessment by an experienced physiotherapist based on a well-described guideline | 54 | N/A | mean(n,SD) - mean(n,SD) | age, BMI | groups: healthy control*, recurrent low back pain, non-continuous chronic low back pain, continuous low back pain PA: continuous | combined low back pains vs healthy control: PA | All | 6.8(21,3.3) - 10.0815(54,3.943) | None | N/A | None | 3 | 2 | 2 |  |  |  |  |  |  |  |  |
| Alzahrani 2019 ^3^ | CS | UK | adults 45.7±16.7 | general public | LTPA assessed by Physical Activity and Sedentary Behaviour Assessment Questionnaire (PASBAQ) | chronic back conditions | structured questionnaire | 60134 | 0.08 <65yr: 0.093 >=65yr: 0.056 M: 0.0854 F: 0.0751 | aOR+SE | age, sex, ethnicity, BMI, smoking, education, occupation, employment status, psychological distress, cardiovascular disease, cancer , musculoskeletal conditions (arthritis/rheumatism/fibrositis, and other problems of bones/joints/muscles). | age: <65yr, >=65yr, all* gender: M, F, both* LTPA (MET-hr/w): inactive (no PA)*, insufficiently active (<7.5), sufficiently active (7.5-1.5), very active (>=15) chronic back conditions: yes*, no | all ages, both genders: SA -> chronic back conditions | All | 0.81+0.0661 | age ages, both genders: IA -> chronic back conditions age ages, both genders: VA -> chronic back conditions <65y, both genders: SA -> chronic back conditions >=65y, both genders: SA -> chronic back conditions all ages, M: SA -> chronic back conditions all ages F: SA -> chronic back conditions | 0.89+0.0573 0.77+0.0532 0.66+0.0690 0.81+0.1668 0.82+0.0932 0.80+0.0917 | None |  |  |  | T | F | T | N/A | T | T | T | T |
| Amorim 2019 ^4^ | CS | Spain | middle-aged and elderly 53.41 ± 7.20 | general public | weekly frequency and total duration of walking, moderate PA and vigorous PA >10/session inquired by structured questionnaire | LBP | structured questionnaire 1. "Have you ever suffered from chronic LBP?" 2. "When was the last time you experienced LBP?" 3. "How long has it been since you have had a whole month pain free?" | 1059 | 0.0683 | aOR+SE | age, sex, depression, anxiety | any LTPA: moderate, vigorous, total LBP: yes*, no | total LTPA -> LBP | All | 0.36+0.3007 | moderate LTPA -> LBP vigorous LTPA -> LBP | 0.44+0.290 0.12+1.050 | Murica Twin Registry |  |  |  | T | F | T | N/A | T | T | F | T |
| Andias 2022 ^5^ | CH | Portugal | adolescents 16.4 ± 1.15 | secondary school students | IPAQ for adolescents | CMSP | adapted NMQ inquiring pain in the last 3 months, at least once a week | 215 | N/A | OR+SE | N/A | LTPA: continuous (min/w) total PA CMSP: yes, no | LTPA -> CMSP | N/A | 0.9995+0.0023 | N/A | N/A | None | 4 | 0 | 3 |  |  |  |  |  |  |  |  |
| Andorsen 2017 ^6^ | CH | Norway | adults 43.2 ± 11.0 | general public | structured quesstionnaire | chronic musculoskeletal complaints (MSCs)s | structured questionnaire | 4496 | 0.598 M: 0.530 F: 0.682 | aOR+SE | age, gender, current smoking, self-perceived general health, mental health complaints, educational level, BMI | gender: both*, M, F PA (hr/w): sedentary (0), low (<3), moderate (3-5), high (>=6)* MSCs: any, neck/shoulders, arms/hands, upper back, lumbar back, hip/leg/feet, other regions | both genders: high PA (vs sedentary) -> any MSC | All | 0.8610+0.1807 | both genders: low PA (vs high) -> any MSC both genders: moderate PA (vs high) -> any MSC M: high PA (vs sedentary) -> any MSC F: high PA (vs sedentary) -> any MSC both genders: high PA (vs low) -> lumbar back MSCs both genders: high PA (vs low) -> neck/shoulder MSCs | 1.1889+0.1253 1.1850+0.1236 1.0040+0.2256 0.6207+0.3086 0.9274+0.1962 0.7369+0.1928 | Tromsø study 1-7 | 4 | 2 | 3 |  |  |  |  |  |  |  |  |
| Astfalck 2010 ^7^ | CC | Australia | adolescents 15.6 ± 0.52 | general public | min/w MVPA by Multimedia Activity Recall for Children and Adolescents (MARCA) | non-specific CLBP | clinical assessment by a validated classification system | 56 | None | mean(N,SD) - mean(N,SD) | None | group: control*, NSCLBP MVPA (min/w): continuous | NSCLBP -> MVPA | N/A | 1158(28,618) - 919(28,449) | N/A | N/A | sampled from Western Australian Pregnancy Cohort (Raine) Study | 3 | 0 | 2 |  |  |  |  |  |  |  |  |
| Auvinen 2008 ^8^ | CS | Finland | adolescents 15-16 | general public | hr/w brisk and light PA after school, and PA commuting to school inquired by validated questionnaire as LTPA level | LBP | structured questionnaire: "Have you had any pain or aching in your low back area during the past six months?"" | 5999 M: 2828 F: 3187 | 0.396 M: 0.311 F: 0.469 | aOR+SE | BMI, smoking | gender: M, F LTPA: very active (>6hr/w brisk PA), active (4-6hr/w brisk PA), moderately active (2-3hr/w brisk PA)*, lightly active (1hr/w brisk PA or <0.5hr/w brisk PA + >2hr/w light/commuting PA), inactive (<0.5hr/w brisk PA, <2hr/w commuting PA) LBP: reporting LBP*, consultation LBP | M, F: moderately active (vs inactive) -> reporting LBP | All | M: 1.0638+0.1556 F: 0.8547+0.1428 | M, F: lightly active -> reporting LBP M, F: active -> reporting LBP M, F: very active -> reporting LBP | M: 0.8379+0.1295 F: 1.0960+0.0949 M: 1.2262+0.1196 F: 1.1696+0.1061 M: 1.1566+0.1223 F: 1.5090+0.1386 | Northern Finland birth cohort 1986 at 2001-2002 follow-up |  |  |  | T | F | T | N/A | T | T | T | T |
| Baker 2019 ^9^ | CS | USA | M elderly 73.99 ± 7.13 | general public | structured questionnaire inquiring frequency of participating in vigorous, moderate, and mild exercises respectively, with a proper definition for each intensity level, and frequencies >1/w are considered regular PA (positive response) | pain | structured questionnaire: 1. "Are you often troubled with pain?" 2. "How bad is the pain most of the time: mild, moderate or severe?" | 4486 | None | aOR+SE | age, education, health conditions, physical functioning , race group | PA: light, moderate/vigorous pain: no pain*, mild pain, moderate pain, severe pain | MVPA -> moderate pain | all | 0.774+0.0886 | light PA -> moderate pain | 0.9609+0.0872 | Health and Retirement Study at 2008 follow-up |  |  |  | T | T | F | N/A | T | T | F | T |
| Barbosa 2022 ^10^ | CS | Brazil | adults 43.1 ± 9.4 | teachers | validated questionnaire using yes/no questions | back pain | structured questionnaire "During the pandemic, with the changes in your usual activities, did you start to have any back pain (upper, mid or low)?" | 15276 | 0.647 | aOR+SE | age | PA: stayed active*, positive changes, stayed inactive, negative changes back pain: yes, no | stayed inactive (vs stayed inactive) -> back pain | All | 0.909+0.02079 | stayed active (vs negative changes) -> back pain | 0.8937+0.02279 | ProfSMoc Project-Minas COVID Stage |  |  |  | T | F | T | N/A | T | T | F | T |
| Barro 2015 ^11^ | CS | Brazil | adults 30.8 ± 8.5 | shift workers | structured questionnaire inquiring weekly duration of PA undertaken for leisure or transportation | 12mo MSP | adapted NMQ inquiring the 12mo-prevalence of pain ("none", "sometimes", "often", or "always") and its location (upper extremities, lower extremities, or trunk) | 1103 | 0.179 | Raw counts | N/A | LTPA (min/w): inactive (<150)*, active (>=150) MSP: upper extremity, trunk*, lower extremity | active -> MSP at the trunk | all | 63, 336, 126, 578 | None | N/A | None |  |  |  | T | F | F | N/A | T | F | T | T |
| Basler 2008 ^12^ | CC | Germany | elderly 71.33 ± 5.02 | general public | Freiburg Activity Questionnaire | CLBP | medical diagnosis: CLBP due to osteoporosis or degenerative spine disorders regardless of previous surgical intervention to the spine | 162 | None | mean(N,SD) - mean(N,SD) | None | group: CLBP, controls LTPA: diary, questionnaire (continuous)* | CLBP vs controls: LTPA questionnaire (continuous) | None | 39.95(103,27.58) - 46.01(59,33.0) | N/A | N/A | None | 3 | 0 | 2 |  |  |  |  |  |  |  |  |
| Batista 2022 ^13^ | CS | Brazil | adults 33.9 ± 8.6 | fruit-growing workers | structured questionnaire inquiring account the time of postures adopted outside of work and the performance of physical activity. | high-intensity shoulder pain | intensity of shoulder pain in the past 3 months assessed with 0-10 NRS, positive shoulder pain at NRS > 4 | 180 | N/A | aOR+SE | gender, BMI, co-morbidities, alcohol consumption, use of medication, child at home, work-related variables, family income, daytime fatigue, fatigue after work, sleep problems | LTPA: sufficient, insufficient (by WHO recommendations) High-intensity shoulder pain: NRS > 4, NRS > 3 | insufficient LTPA -> shoulder pain with NRS > 4 | insufficient LTPA -> shoulder pain with NRS > 3 | 2.85+0.4998 | insufficient LTPA -> shoulder pain with NRS > 3 | 2.39+0.3262 | None |  |  |  | T | T | T | N/A | T | T | T | T |
| Björck-van Dijken 2008 ^14^ | CS | Sweden | young adults-elderly 54.6 ± 12.6 | general public | structured questionnaire inquiring any moderate physical activity 1-2hr/w e.g. jogging, tennis, swimming, gymnastics | chronic LBP: LBP that had lasted continuously for more than 6 months | structured questionnaire inquiring for yes/no, duration and frequency of LBP | 5798 | 0.427 M: 0.387 F: 0.461 | aOR+SE | BMI, age, sex, education, regular smoking , community size | LTPA last year: low, otherwise* | high LTPA last year (vs low) -> CLBP | N/A | 0.8621+0.0677 | N/A | N/A | MONICA study |  |  |  | T | F | F | N/A | T | T | F | T |
| Björkegren 2009 ^15^ | CC | Sweden | adults 49.0 ± 8.4 | general public | LTPA inquired by structured questionnaire | Fibromyalgia | Fibromyalgia diagnosis by record linkage, confirmed by the Complaint Score sub-scale of the Gothenburg Quality of Life Instrument | 539  Cases: 138 Referents: 401 | 0.271 | OR+SE | age, sex, residential area | LTPA: low, otherwise Fibromyalgia: cases, referents | low LTPA -> fibromyalgia case | N/A | 0.9859+0.1604 | N/A | N/A | None | 3 | 2 | 3 |  |  |  |  |  |  |  |  |
| Björnsdóttir 2013 ^16^ | CS | Iceland | adults 18-79 | general public | IPAQ-SF | CP | 20-item structured questionnaire inquiring chronic backpain, chronic neck symptoms, and fibromyalgia | 5756 | 0.247 | aOR+SE | age monthly income BMI smoking educational level residential area | Gender: M, F LTPA: adequate, inadequate | M, F: inadequate LTPA -> CP | N/A | M: 1.00+0.1274 F: 1.20+0.0991 | N/A | N/A | Public Health Instiute of Iceland, 2007 |  |  |  | T | F | T | N/A | T | T | F | T |
| Bollinger 2020 ^17^ | CS | USA | adults 27.6 | general public | structured questionnaire inquiring if activity level >=150min/w | CP | structured questionnaire inquiring 3mo frequency and intensity of pain | 1334 | 0.374 | aOR+SE | number of chronic pain conditions, age, gender, race, marital status, educational attainment, family income, smoker, alcohol consumption, diabetes history, self-rated health status, fatigue , activity limitation, BMI, depression, anxiety, >10 healthcare visits in past year , mental health visit | PA: highly-sufficiently, insufficiently-inactive* CP: yes*, no | highly-sufficiently PA -> CP | N/A | 0.95+0.238 | N/A | N/A | NHIS 2014 of NHPI population |  |  |  | T | T | F | N/A | T | T | F | T |
| Brady 2016 ^18^ | CH | Australia | F young adults 24.5 ± 1.5 | general public | MET inquired by locally validated questionnaire | 12mo back pain | structured questionnaire inquiring 12mo-prevalence of back pain: "sometimes/often" or "never/rarely" | 9105 | 0.429 | aOR+SE | age, weight, height, education status, depression | PA baseline: none, low, moderate, high back pain: yes*, no | moderate-high PA (vs none-low) -> back pain | None | 0.877+0.02899 | N/A | N/A | Australian Longitudinal Study of Women's Health (ALSWH) | 3 | 2 | 3 |  |  |  |  |  |  |  |  |
| Brady 2017 ^19^ | CH | Australia | F middle-aged 49.5 ± 1.5 | general public | MET inquired by validated local questionnaire, vigorous PA defined as "the ones that make you puff and pant, like vigorous aerobics, competitive sport, vigorous cycling, running, swimming" | back pain | structured questionnaire inquiring 12mo-prevalence of back pain: "sometimes/often" or "never/rarely" | 11478 | 0.547 | aOR+SE | age, weight, height, employment status, depression, menopausal status, smoking status | vigorous LTPA: yes, no* back pain: yes*, no | vigorous LTPA -> back pain | N/A | 0.81+0.0219 | N/A | N/A | Australian Longitudinal Study on Women's Health (ALSWH) | 3 | 2 | 3 |  |  |  |  |  |  |  |  |
| Brindova 2015 ^20^ | CS | Slovakia | adolescents 13.1 ± 1.35 | general public | PA assessed by structured questionnaire as d/w with >=60min MVPA | backache | HBSC-SCL inquiring frequency of backache during the last 6 months. >=1/w is considered positive | 8042 | 0.18 | aOR+SE | age, gender, hours/day working with PC or playing PC games | PA: 7d/w*, 3-6d/w, <=2d/w backache: yes*, no | 2-3hr/d computer-based activities: 7d/w PA (vs otherwise) -> backache | None | 1.70+0.18958 | N/A | N/A | None |  |  |  | T | T | F | N/A | T | T | T | T |
| Brown 2000 ^21^ | CS | Australia | F young adults 18-23 F middle-aged 45-50 F elderly 70-75 | general public | structured questionnaire inquiring weekly frequency of light and moderate-vigorous with >=20min/session, with examples respectively | BP | structured questionnaire | young adults: 14502 middle-aged: 13609 elderly: 11421 | young adults: 0.446 middle-aged: 0.570 elderly: 0.596 | aOR+SE | smoking status, alcohol consumption, number of diets in last year, BMI, low iron, type of contraception or hormone, stress level, education, area of residence, menopausal status | age group: young adults, middle-aged, elderly PA: none/very low (<=1/w moderate PA)*, low-moderate (2-4/w moderate PA or 1-2/w vigorous PA), moderate-high (5-8/w moderate PA or 2-4/w vigorous PA), high (8-13/w moderate PA or 5-8/w vigorous PA), very high (>8/w vigorous PA, or equivalent amounts of moderate PA) BP: yes*, no | each age group: moderate-high PA -> BP | All | 0.7647+0.0599; 0.8497+0.0569; 0.7792+0.0620 | each age group: low-moderate PA -> BP each age group: high PA -> BP each age group: very high PA -> BP | 0.8340+0.0610; 0.8899+0.0544; 0.9053+0.0628 0.7288+0.0663; 0.8385+0.0637; 0.6769+0.0788 0.7217+0.0775 | Australian Longitudinal Study on Women's Health (ALSWH) baseline in 1996 |  |  |  | T | F | F | N/A | T | T | F | T |
| Burton 1991 ^22^ | CS | UK | children 10-11 adults 16-84 | general public | structured questionnaire inquiring any (amateur-level leisure) sports participation, i.e. physical education lessons for children aged 10-11y, and at least weekly participation for adults aged 16-84y | low back trouble | structured questionnaire: "Have you ever had pain in your low back (other than normal ache/stiffness after, say, gardening)?" Positive responses were followed by frequency and age-at-onset questions | 958 | children: 0.293 adults: 0.386 | Raw counts | N/A | Sports: yes, no*, regular, heavy low back trouble: yes, no, recurrent, isolated onset-age of low back trouble: <=25y, >25y | sports -> recurrent low back trouble | All | children: 84, 184, 53, 80 adults: 124, 133, 98, 131 | None | N/A | None |  |  |  | T | F | T | N/A | F | F | T | T |
| Carpintero-Rubio 2021 ^23^ | CS | Spain | adults / | general public | structured questionnaire inquiring time, type, frequency and feeling of effort about sports activity | MSP | structured questionnaire inquiring perception of musculoskeletal health, any ailment of the musculoskeletal system, duration, location, current/weekly average/worst intensity, interference, and coping strategies of pain | 3247 | 0.504 | Raw counts | N/A | confinement: before*, after /d sports activity: >1hr, <1hr* pain: yes, no* | before confinement: >1hr -> pain | during confinement: >1hr -> pain | 866, 1022, 392, 386 | during confinement: >1hr -> pain | 458, 421, 754, 499 | most pain responses are chronic |  |  |  | T | F | F | N/A | T | F | F | T |
| Chaiklieng 2020 ^24^ | CS | Thailand | adults 37.49 ± 6.31 | workers | structured questionnaire | LBP | structured questionnaire inquiring 7d- and 6mo-prevalence | 446 | 0.5447 | aOR+SE Raw counts | age, gender, work experience, family history of LBP, repetitive working, prolonged sitting , working hour/d, fixed posture | exercise: none*, irregular, regular, irregular-regular LBP: 6mo*, 7d | irregular-regular exercise -> 6mo LBP | All | 0.6268+0.2332 | regular exercise -> 6mo LBP | 66, 113, 67, 56 | None |  |  |  | T | F | F | N/A | T | T | F | T |
| Chun 2018 ^25^ | CS | Korea | middle-aged 63.99 ± 1.97 | general public | structured questionnaire inquiring d/mo with moderate PA >=10min/d | MSP at knee, hip, and lower back | structured questionnaire inquiring MSP for >30d in the past 3mo | 17108 | 0.391 | Raw counts | N/A | Moderate exercise: never*, 1-3d/w, 4-6d/w, everyday MSP: any*, knee joint pain, hip joint pain, low back pain | 4-6d/w moderate exercise -> any MSP | 1-3d/w, everyday moderate exercise -> any MSP | 912, 1864, 3400, 5299 | 1-3d/w moderate exercise -> any MSP everyday moderate exercise -> any MSP | 1669, 3173, 3400, 5299 223, 567, 3400, 5299 | KNHANES 2010-2012, 2013-2015 |  |  |  | T | T | F | N/A | T | F | F | T |
| Citko 2018 ^26^ | CS | Poland | adults 40.99 ± 6.66 | medical personnel | IPAQ-SF | nonspecific LBP (nsLBP): recurring nsLBP as >=3 episodes of acute pain in 12mo chronic nsLBP as persistent pain >=12w | NMQ | 609 | 0.1275 | OR+SE | N/A | PA: sedentary*, moderate, increased, high nsLBP: recurrent, chronic* | PA not sedentary -> chronic nsLBP | PA not sedentary -> recurrent nsLBP | 9.08+0.3099 | PA not sedentary -> recurrent nsLBP | 0.279+0.1735 | None |  |  |  | F | F | T | N/A | T | F | T | T |
| Constantino Coledam 2019 ^27^ | CS | Brazil | adults 40.1 ± 8.88 | teachers | structured questionnaire: "Do you practice sports?", IPAQ-LF | MSS | NMQ inquiring 12mo-prevalence | 530 | 0.695 vs 0.623 | aPR+SE | gender, perception of school infrastructure, TV viewing time, high stress, common mental disorder, diagnosed musculoskeletal disease | PA: <150min/w*, >=150min/w MSS: upper limbs, lower limbs, back and neck* | PA >=150min/w -> back and neck MSS | All | 0.99+0.077 | None | N/A | None |  |  |  | T | F | T | N/A | T | T | T | T |
| Correa-Rodríguez 2019 ^28^ | CC | Spain | F adults 56.1 ± 9.2 | general public | IPAQ-SF | fibromyalgia | record linkage | 203 95 cases 108 controls | 0.696 | Raw counts | menopause status | LTPA: low, moderate, high Fibromyalgia: case, control | moderate-high LTPA -> fibromyalgia case | high LTPA -> fibromyalgia case | 54, 77, 39, 17 | high LTPA -> fibromyalgia case | 10, 24, 83, 70 | None | 2 | 1 | 2 |  |  |  |  |  |  |  |  |
| D'Onise 2010 ^29^ | CH | Australia | adults 45.43 ± 11.20 | general public | telephone interview using validated local questionnaire | current shoulder pain | telephone interview inquiring any experience of shoulder pain, aching or stiffness on most days lasting >=1mo ever, followed by Shoulder Pain And Disability Index (SPADI) | 1502 | None | aOR+SE | age, sex | LTPA (MET-min/2w): sedentary (<100)*, low (100-1600), moderate (1600-3200 or >3200 and >2hr vigorous exercise), high (>3200 and >2hr vigorous exercise) shoulder pain: yes*, no | not sedentary LTPA -> shoulder pain | None | 0.857+0.1529 | N/A | N/A | NWAHS | 4 | 2 | 2 |  |  |  |  |  |  |  |  |
| Decharat 2016 ^30^ | CS | Thailand | adults 37.3 | dental health workers | structured interview inquiring PA | MSS | structured interview inquiring 12mo-prevalence and location | 124 | 0.339 | Raw counts | N/A | exercise: yes, no MSS: lower back, shoulders, neck | exercise -> lower back MSS | all | 17, 42, 22, 43 | exercise -> shoulders MSS exercise -> neck MSS | 12, 47, 47, 18 3, 56, 45, 20 | None |  |  |  | F | F | F | N/A | T | F | F | T |
| Drozda 2011 ^31^ | CS | Poland | adolescents 16.46 ± 1.40 | students | Structured questionnaire inquiring hours of additional physical activity and frequency of activities requiring physical work | BP | Structured questionnaire inquiring yes/no, frequency, location and precipitating circumstances of back pain | 1475 | 0.44 | Raw counts | None | additional PA: none*, 1hr/w, 2-3hr/w, >=4hr/w Back/neck pain: no pain*, at least several episodes a month | 2-3hr/w -> at least several episodes a month of pain | 1hr/w -> at least several episodes a month of pain >=4hr/w -> at least several episodes a month of pain | 99, 161, 44, 56 | 1hr/w -> at least several episodes a month of pain >=4hr/w -> at least several episodes a month of pain | 91, 146, 44, 56 67, 126, 44, 56 | None |  |  |  | T | F | F | N/A | T | F | F | F |
| Eggermont 2009 ^32^ | CS | USA | elderly 78.2 ± 5.3 | general public | Physical Activity Scale for the Elderly (PASE) | MSP | structured interview inquiring pain in musculoskeletal sites (hand or wrist, shoulder, back, hip, knee, or foot) lasting 3 or more months in the previous year and present in the previous month | 544 | 0.722 | Raw counts | N/A | PA quartile: 1st*, 2nd, 3rd, 4th Pain: no pain*, single-site pain, multisite pain, widespread pain | 3rd PA quartile -> any pain | All | 86, 49, 96, 37 | PA 2nd quartile -> any pain PA 4th quartile -> any pain | 81, 59, 96, 37 76, 60, 96, 37 | MOBILIZE Boston Study |  |  |  | T | F | T | N/A | T | F | F | T |
| El-Metwally 2007 ^33^ | CH | Finland | children 10.8 | students | structured questionnaire inquiring d/w with >=30min exercise to breathlessness | non-specific MSP | structured questionnaire inquiring any MSP symptoms at neck, upper limb, chest, upper back, lower back and buttock during past 3mo and their frequencies, any prior trauma causing the pain | 1113 | 0.194 | aOR+SE OR+SE | age, sex, psychosomatic symptoms, hypermobility score | PA: 0-2/w*, 3-4/w, 5-7/w MSP: non-traumatic*, traumatic | 3-4/w PA -> non-traumatic MSP | All | 1.0675+0.1870 | 5-7/w PA -> non-traumatic MSP (unadjusted) | 1.1269+0.2257 | None | 4 | 2 | 2 |  |  |  |  |  |  |  |  |
| Fanavoll 2016 ^34^ | CH | Norway | adults >20 | general public | structured questionnaire inquiring frequency, intensity and duration of leisure time physical exercise | CNSP | NMQ | 29496 M: 12693 F: 8925 | None | aRR+SE | age, BMI, smoking, occupation, education, psychological well-being | M, F perceived work stress: not at all*, rarely, a certain amount, almost all the time exercise*: inactive*, <2hr/w, >=2hr/w usual exercise intensity: inactive*, low, moderate/high* CNSP: yes, no | M, F without perceived work stress: >=2hr/w exercise -> CNSP | All | M: 0.74+0.194 F: 0.73+0.164 | M, F without perceived work stress: <2hr/w exercise -> CNSP M, F without perceived work stress: moderate/vigorous exercise -> CNSP | M: 0.59+0.1664 F: 0.99+0.1003 M: 0.68+0.1689 F: 1.00+0.1191 | HUNT 1-2 | 4 | 2 | 2 |  |  |  |  |  |  |  |  |
| Feldman 2002 ^35^ | CH | Canada | adolescents 13.8 ± 0.1 | students | structured questionnaire inquiring any participation of sports activities, its duration and frequency for calculation of MET | neck/upper limb pain | structured questionnaire inquiring neck and upper limb pain occurring >=1/w in preceding 6mo | 502 | None | aOR+SE | playing any musical instrument, working vs not working, mental health index, high growth (>5cm/6mo), height, BMI, age, gender, smoker | Sport activity -> neck/upper limb pain in GEE analysis | Sport activity -> neck/upper limb pain in GEE analysis | None | 1.00+0.00510 | N/A | N/A | None | 3 | 2 | 2 |  |  |  |  |  |  |  |  |
| Feller 2020 ^36^ | CS | USA | elderly 68.91 ± 10.21 | general public | structured questionnaire inquiring if, at leisure, play sports or exercise, or walk for >=20min, >=1/mo | pain | structured questionnaire "are you often troubled by pain?" | 7541 | 0.476 | Raw counts | N/A | exercise: regular, not regular pain: yes, no | regular exercise -> pain | N/A | 1191, 2656, 1748, 1925 | N/A | N/A | Health and Retirement Study (HRS) 2014 |  |  |  | T | T | F | N/A | F | F | F | T |
| Fernández-de-las-Peñas 2011 ^37^ | CS | Spain | adults | general public | structured questionnaire: Did you practice any physical activity, such as walking or practicing sports during your free time? | neck pain (NP), LBP | structured questionnaire: 1. Have you suffered pain over the previous 12 months? 2. Has your medical physician confirmed the diagnosis? | 29478 | LBP: 0.201 NP: 0.195 | Raw counts | N/A | physical exercise: yes, no* pain: LBP*, NP | physical exercise -> LBP | physical exercise -> NP | 3454, 14080, 2401, 9544 | physical exercise -> NP | 3402, 14132, 2329, 9615 | Spanish National Health Survey (SNHS) |  |  |  | T | T | F | N/A | F | F | F | F |
| Fernández-de-las-Peñas 2013 ^38^ | CS | Spain | adults 53.8 ± 14.2 | general public | structured questionnaire: Do you practice any leisure time moderate or intense physical activity for at least 3 days per week? | neck pain (NP), LBP | structured questionnaire: 1. Have you suffered the pain over the last 12 months? 2. Have you visited your medical doctor for this symptom? | 22188 | LBP: 0.082 NP: 0.049 | aPR+SE | sex, age, marital status, educational level, self-rated health, monthly income, smoking habit, obesity , heart disease, headache, chronic bronchitis, osteoporosis, injuries | Physical exercise: yes, no* pain: NP, LBP | physical exercise -> LBP | physical exercise -> NP | 1.063+0.0703 | physical exercise -> NP | 1.233+0.0752 | None |  |  |  | T | T | F | N/A | T | T | F | T |
| Fjeld 2022 ^39^ | CS | Norway | adults 56.8 ± 11.1 | general public | Saltin-Grimby physical activity scale | chronic pain | GRIP, pain intensity, pain-related distress, and task interference assessed by 11-point NRS | 17421 | N/A | aPR+SE | age, education level, smoking, occupational physical activity, sex | LTPA: inactive*, low, moderate, vigorous CP: CP, moderate-to-severe CP | moderate LTPA -> CP | All possible combinations | 0.91+0.0186 | low LTPA -> CP vigorous LTPA -> CP moderate LTPA -> moderate-to-severe CP | 0.96+0.0174 0.80+0.0448 0.69+0.0519 | Tromsø study 7 |  |  |  | T | T | T | N/A | T | T | T | T |
| García-Heras 2022 ^40^ | CS | Spain | M adults 36.4294 ± 6.5146 | firefighters | structured questionnaire inquiring hr/w of PA as any exercise/training performed in leisure time | CP | structured questionnaire inquiring prevalence and location of CP | 221 | 0.531 | Raw counts | N/A | PA: low*, moderate, high CP: no*, single-site, multisite | moderate PA -> single-site - multisite CP | All | 79, 49, 26, 23 | high PA -> single-site - multisite CP moderate - high PA -> single-site/multisite CP | 27, 17, 26, 23 106, 66, 26, 23 | None |  |  |  | T | F | F | N/A | F | F | F | T |
| Ghim 2022 ^41^ | CS | USA | adults 41.2037 ± 11.5925 | general public | structured questionnaire: 1. "How often do you do LIGHT OR MODERATE leisure-time physical activities for AT LEAST 10 MINUTES that cause ONLY LIGHT sweating or a SLIGHT to MODERATE increase inbreathing or heart rate?" 2. "How often do you do VIGOROUS leisure-time physical activities for AT LEAST 10 MINUTES that cause HEAVY sweating or LARGE increases in breathing or heart rate?" Or inquired with a list of PAs corresponding to each intensity level | Back pain | structured questionnaire: "Have you ever been told by a doctor or health professional that you have back pain?" | 890 | None | aOR+SE | chronological age, gender, race, marital status, federal poverty level, working status, insurance coverage | PA: inactive(<150min/w PA)*, aerobically active (>=150min/w PA, <=1d/w muscle training PA), muscularly active (>=150min/w PA, >=2 d/w muscle-training PA) | aerobically active -> back pain | muscularly active -> pain | 0.7884+0.1745 | muscularly active -> pain | 0.6859+0.1034 | NHIS 2018 |  |  |  | T | T | F | N/A | T | T | F | T |
| Grasdalsmoen 2020 ^42^ | CS | Norway | young adults 23.2 ± 3.3 | students | validated questionnaire inquiring frequency, intensity and duration per session of physical exercise | ICD-11 (moderate to severe) CP | The Graphical Index of Pain (GRIP) | 36625 M: 12025 F: 24600 | 0.581 M: 0.495 F: 0.635 | M:aOR+SE, F:aOR+SE | age, marital status, immigrant status, economic activity (active/inactive), BMI, alcohol use and problems, sleep duration, self-reported depression | M, F physical exercise: never/less than once a week*, once a week, 2-3 times per week, almost every day ICD-11 CP: CP*, moderate to severe CP | M, F: 2-3 times per week physical exercise -> ICD-11 CP | All | M:0.736+0.0554, F:0.916+0.0417 | M, F: once a week physical exercise -> ICD-11 CP M, F: almost every day physical exercise -> ICD-11 CP M, F: 2-3 times per week physical exercise -> ICD-11 moderate to severe CP | M:0.833+0.0672, F:0.954+0.0267 M:0.685+0.0594, F:0.846+0.0482 M:0.647+0.0980, F:0.895+0.0512 | SHoT2018 study |  |  |  | T | T | T | N/A | T | T | T | T |
| Graup 2014 ^43^ | CS | Brazil | adolescents 13.56 ± 2.02 | students | Activity Questionnaire for Older Children (PAQ-C) and Adolescents (PAQ-A) | nonspecific lumbar pain | Structured questionnaire: "Have you ever had pain or discomfort in your back, in the lumbar region?", with a drawing indicating the location of the lumbar region provided. | 1455 | 0.197 | aOR+SE | socioeconomic level, sex, age, sedentary behavior | PA tercile: most active*, intermediate, least active lumbar pain: yes*, no | most active (vs least active) -> lumbar pain | All | 1.0990+0.2125 | most active (vs intermediate) -> lumbar pain | 1.3170+0.2091 | None |  |  |  | T | F | T | N/A | T | T | F | T |
| Halonen 2019 ^44^ | CH | Sweden | adults 54.1 ± 11.3 | general public | structured questionnaire inquiring frequency of PA | LBP | structured questionnaire inquiring 3mo prevalence of LBP and its interference with daily activities | 17962 | 0.282 | aRR+SE | age, sex, study survey, education, twisting, lifting , BMI, smoking, depressive symptoms, sleep problems | PA: regularly, sometimes, never or seldom* LBP: incident*, recurrent | regular PA -> incident LBP | All | 1.00+0.0535 | regular PA -> recurrent LBP | 0.972+0.0248 | Swedish Longitudinal Occupational Survey of Health study | 4 | 2 | 2 |  |  |  |  |  |  |  |  |
| Harithasan 2022 ^45^ | CS | UK | young adults 18-25 | undergraduate students | self-perceived exercise inquired by online survey | MSP | NMQ | 179 | None | OR+SE | N/A | self-perceived exercise: yes, no* MSP: shoulder, wrist/hand, upper back, lower back, hips and thigh, knee | self-perceived exercise -> LBP | All | 0.963+0.3376 | self-perceived exercise -> upper back pain | 0.475+0.304 | None |  |  |  | T | F | F | N/A | F | F | T | T |
| Hartvigsen 2007 ^46^ | CH | Denmark | elderly 70-100 | general public | Participants were asked if they at present were engaged in strenuous physical activity, such as heavy gardening, long (more than half an hour) walks or bike rides, sports, or dancing, and its frequency if yes. | LBP | modified NMQ inquiring how many days out of the past year they had had LBP (pain, stiffness, or other discomfort) | 1844 | 0.05 | aOR+SE | None (twin study) | PA: sedentary*, any light PA, any strenuous PA* strenuous PA: no*, <1/w, >=1/w LBP: any LBP past year, LBP >30d past year* | any strenuous PA -> LBP >30d past year | All | 0.514+0.242 | any light PA -> LBP >30d past year <1/w strenuous PA -> LBP >30d past year >=1/w strenuous PA -> LBP >30d past year | 0.852+0.320 0.654+0.738 0.509+0.237 | Longitudinal Study of Aging Danish Twins (LSADT) 2001 | 4 | 2 | 2 |  |  |  |  |  |  |  |  |
| Heuch 2016 ^47^ | CH | Norway | adults | general public | structured questionnaire inquiring light activity, which not involve sweating or breathlessness, and hard physical activity, which causes sweating or shortness of breath. Duration (hr/w) of light and hard physical activity was also inquired. | CLBP | structured questionnaire: 1. During the last year, have you had pain and/or stiffness in your muscles and limbs that has lasted for at least 3 consecutive months? 2. Where did you have pain and/or stiffness? | 18068 M: 9616 F: 8452 | 0.190 M:0.161 F: 0.217 | M:aRR+SE, F:aRR+SE | age, education, work status, PA at work, BMI, smoking | M, F LTPA (hr/w): <1 light + 0 hard*, >=1 light + 0 hard, <1 hard, 1-2 hard, >=3 hard | M, F: 1-2hr/w hard LTPA -> CLBP | All | M:0.812+0.0906, F:0.891+0.0742 | M, F: <1hr/w hard LTPA -> CLBP M, F: >=3hr/w hard LTPA -> CLBP | M:0.962+0.0845, F:0.992+0.0667 M:0.961+0.1002, F:1.045+0.0994 | HUNT2-HUNT3 | 4 | 2 | 2 |  |  |  |  |  |  |  |  |
| Hill 2004 ^48^ | CH | UK | adults 51 ± 16 | general public | structured demographics questionnaire inquiring perceived physical activity for age ("more or the same"or"less"compared with others), gardening, do-it-yourself work ("often"or"not of-ten"), daily walking, cycling, and hours watching TV (categories were dichotomised according to the median number of 3 hours per day) | persistent neck pain | structured questionnaire inquiring 1mo prevalent neck pain using shaded mannequin | 1359 | 0.543 | aOR+SE | age, alcohol intake, general health, GHQ, LBP, neck injury, employment status, cycling each day | PA: average or better, less than average*(inverted) 12mo persistent NP: yes, no* | average or better PA -> 12mo persistent NP | None | 0.847+0.208 | N/A | N/A | None | 4 | 2 | 2 |  |  |  |  |  |  |  |  |
| Holmberg 2003 ^49^ | CS | Sweden | M adults 50 | farmers and matched non-farmers (general public) | assessed by structured interview as sedentary, slightly active, moderately active and vigorously active, according to WHO 1968 guidelines | MSS | structured questionnaire: 1. "Have you ever and not only occasionally had problems in the neck, shoulder or shoulder joint areas with ache, pain or discomfort?" 2. "Have you ever had problems with numbness or a pricking sensation in your hands?" 3. "Have you ever had problems in the low back area with ache, pain or discomfort?" 4. "Have you at any time had problems in the hip area with ache, pain or discomfort?" | 2351 Farmers: 1221 Referents: 1130 | None | OR+SE aOR+SE | farmer vs referent, workload, vibrations, heavy lifting, difficult working positions, work hours, sleep hours, time in current job, self-employment, leisure-time activity, BMI, total muscle strength, physical capacity | group: all*, farmers, referents LTPA: yes, no* MSS: neck/shoulder, hand/forearm, low back*, hips, knees OR+SE, aOR+SE* | all: LTPA -> low back symptoms (OR+SE) | All | 0.810+0.0816 | all: LTPA -> neck/shoulder symptoms (OR+SE) farmers: LTPA -> neck/shoulder symptoms (aOR+SE) referents: LTPA -> neck/shoulder symptoms (aOR+SE) | 0.958+0.0795 1.199+0.3014 0.683+0.1293 | aOR+SE were not reported for LTPA -> low back symptoms category due to statistical insignificance. |  |  |  | T | T | T | N/A | T | T | F | T |
| Holth 2008 ^50^ | CH | Norway | adults 56.67 ± 14.38 | general public | structured questionnaire inquiring the intensity, average duration and weekly frequency of physical exercise at leisure time | MSCs | modified NMQ, asking whether participants had suffered pain or stiffness in muscles and joints lasting at least 3 months during the last year, the number of days during the last moth with such complaints, and the area(s) of the body with the complaints | 39520 | 0.567 | adjusted counts | age, gender, BMI, smoking, education | baseline-followup PA: inactive-inactive*, very low-very low, low-low, medium-medium, high-high, increased level, decreased level, missing chronic MSCs: any*, widespread, non-widespread | medium PA -> any chronic MSCs | All | 385, 540, 1165, 893 | very low PA -> any chronic MSCs low PA -> any chronic MSCs high PA -> any chronic MSCs increased PA -> any chronic MSCs | 12147, 11524, 1165, 893 1285, 1416, 1165, 893 229, 329, 1165, 893 1089, 1253, 1165, 893 | HUNT 1-2 | 4 | 2 | 2 |  |  |  |  |  |  |  |  |
| Hulsegge 2011 ^51^ | CH | Netherlands | children 10-12 | general public | PA at ages 5y, 7y and 8y assessed by questionnaire for parents, in which hr/weekday of MVPA or walking/cycling to school was inquired. PA at age 11y was assessed by questionnaire for children: 1. With examples provided, "How many days a week are you active (altogether) for at least1full hour a day?"" >=5d is active. 2. ""How many minutes are you normally walking or cycling from home to school and back?"" >60min is active. | long-lasting MSCs | structured questionnaire inquiring children of any experience of 15 listed diseases and complaints, including MSCs in the back, upper extremities and lower extremities:  1. ""Could you mark for the following diseases or conditions whether you have had it in the past 12 months (=last year) and whether you visited your doctor in the past 12 months for that reason?"" ""If "long-lasting" is stated, we mean conditions which bothered you in total for more than 1 month,"" | 2638 | None | aOR+SE | maternal education level, ethnicity, pubertal status, height-for-age and -sex z score, height gain cm/y, weight-for-height z score, weight gain kg/y, mental health status , daytime tiredness, television/computer use, PA from ages 5 to 11y | PA at age 11y: <5d/w*, >=5d/w MSC: back, upper extremity, lower extremity, any MSCs | <5d/w PA at age 11y -> any MSCs | All | 1.2775+0.1148 | <5d/w PA at age 11y -> back complaints (unadjusted) | 0.6387+0.2388 | PIAMA study | 4 | 2 | 2 |  |  |  |  |  |  |  |  |
| Hussain 2016 ^52^ | CH | Australia | adults 48.39 ± 11.22 | general public | Active Australia Survey inquiring LTPA, where >=150min/w MVPA is considered sufficient | LBP | CPGQ | 4974 M: 2197 F: 2777 | None | aOR+SE | age, education, smoking status, dietary guideline index score, BMI , mental component of SF-36 | gender: M, F PA: sufficient*, insufficient LBP: no LBP*, low intensity, high intensity LBP disability: none*, low disability, high disability | M, F: sufficient PA (vs insufficient) -> high intensity LBP | All | M: 0.8252+0.1575, F:1.1164+0.1406 | M, F: sufficient PA (vs insufficient) -> high LBP disability | M: 0.8784+0.1737, F:1.0009+0.1436 | Australian Diabetes, Obesity and Lifestyle (AusDiab) Study | 4 | 2 | 2 |  |  |  |  |  |  |  |  |
| Ílhan 2019 ^53^ | CS | Turkey | elderly 75.5 ± 6.6 | general public | structured interview inquiring frequency of physical activity per week | CP | structured interview inquiring any experience of musculoskeletal system-based pain ongoing >=3mo. 0-10 NRS or mild-moderate-severe indication for severity | 1441 | 0.678 | Raw counts | N/A | PA: never*, 1-2 times/week, everyday chronic pain: no*, yes | everyday PA -> chronic pain | 1-2 times/week PA -> chronic pain | 99, 114, 101, 48 | 1-2 times/week PA -> chronic pain | 82, 56, 101, 48 | only 34.7% subjects have their PA status collected |  |  |  | T | T | F | N/A | T | F | F | T |
| Jia 2022 ^54^ | CS | China | adults 32.3 ± 9.2 | on-duty workers | Physical exercise inquired by structured questionnaire | LBP | Chinese version NMQ | 57501 | 0.188 | aOR+SE | occupational musculoskeletal risk factors, education level, cigarette smoking, BMI, age | physical exercise: frequently, occasionally, no* LBP: case, non-case | occasional/regular physical exercise -> LBP case | None | 0.882+0.0191 | N/A | N/A | None |  |  |  | T | F | F | N/A | T | T | T | T |
| Junqueira 2014 ^55^ | CC | Australia | adults 39.6 ± 12.3 | general public | locally validated Active Australia survey. Results categorised as "no practice" (<2hr/w), "2 hr or more of practice per week" | CLBP | structured questionnaire: 1. self-recall 4w LBP 2. "If you had low back pain in the last 4 weeks, how long was it since you had a whole month without any low back pain?"" | 210 | None | OR+SE | N/A | PA measurement: any vigorous PA, total time spent in vigorous PA, any moderate PA, total time spent in moderate PA, any light walk, total time spent in light walk, any vigorous gardening or heavy work around the house, total time spent in vigorous gardening or heavy work around the house CLBP: yes*, no | total time spent in moderate PA -> CLBP | All | 0.9939+0.5042 | any moderate PA -> CLBP | 0.5940+0.7373 | AUTBACK study | 3 | 0 | 2 |  |  |  |  |  |  |  |  |
| Kaartinen 2019 ^56^ | CH | Finland | adults 34.0 ± 1.2 | general public | leisure-time MET calculated from results of structured questionnaire: "What is your leisure-time physical activity like?" | LBP and NSP | structured questionnaire: "During the past 6 months, have you had any of the following symptoms, and if yes, how often?" | LBP: 3201 NSP: 3207 | None | mean(n,SD) - mean(n,SD) | None | LTPA: continuous (MET-hr/d) pain: LBP, NSP pain frequency: never/seldom, monthly, weekly | LTPA -> weekly vs never/seldom LBP | All | 3.1(510,3.4) - 3.6(1765,3.8) | LTPA -> weekly vs never/seldom NSP | 3.9(876,3.7) - 4.9(1249,3.9) | FinnTwin16 study. Only baseline (cross-sectional) data were used for this review. |  |  |  | T | T | T | F | F | F | F | T |
| Kahere 2021 ^57^ | CS | South Africa | adults 44.51 ± 11.32 | general public | exercise frequency inquired by structured questionnaire | CLBP | previous history, current status, duration, onset, severity, progression, healthcare service utilisation and social implications of LBP inquired by structured questionnaire | 678 | 0.261 | aOR+SE | age, gender, BMI, highest level of education, smoking attitude, alcohol consumption, type of work, sitting posture, use of back support, how long in this job, positive family history of CLBP | exercise frequency: no*, yes CLBP: no*, yes | exercise frequency (yes) -> CLBP | N/A | 0.450+0.396 | N/A | N/A | None |  |  |  | T | T | F | N/A | T | T | F | T |
| Kallings 2021 ^58^ | CS | Sweden | adults 42.11 ± 11.55 | working population | structured questionnaire: "I exercise for the purpose of maintaining / improving my physical fitness, health and well-being ...": "Never", "Sometimes", "1-2 times/week", "3-5 times/week", "At least 6 times/week" | back/neck pain | structured questionnaire: "I have back/neck issues ...": "Very often", "Often", "Sometimes", "Rarely or never" | 44978 | None | aOR+SE | age, sex, occupation, diet, smoking, stress at work, stress overall, BMI, leisure time sitting | sedentary work: almost all time*, 25-75% of the time, almost no time regular exercise: >=1/w*, <1/w back/neck pain: very often/often*, otherwise | almost all time sedentary work: >=1/w regular exercise (vs <1/w) -> very often/often back/neck pain | None | 1.0548+0.0617 | N/A | N/A | Health Profile Assessment database |  |  |  | T | T | F | N/A | T | T | F | T |
| Kamada 2014 ^59^ | CS | Japan | middle-aged - elderly 60.9 ± 10.6 | general public | Japanese version IPAQ-SF | CMSP (CLBP, CKP) | validated Knee Pain Screening Tool (KNEST), 100-point VAS | 4559 | None | aOR+SE | age, sex, education years, self-rated health, chronic disease history, depressive symptom, smoking, BMI, history of joint injuries, pain management (medication use and consultation with physicians) | PA (MET-hr/w): 0, 0.1-8.24, 8.25-23.0*, 23.1-75.3, >=75.4 CMSP: CLBP*, CKP | PA (MET-hr/w) 8.25-23.0 (vs 0) -> CLBP | All | 1.071+0.132 | PA (MET-hr/w) 8.25-23.0 (vs 0) -> CKP PA (MET-hr/w) 0.1-8.24 (vs 8.25-23.0) -> CLBP PA (MET-hr/w) 23.1-75.3 (vs 8.25-23.0) -> CLBP PA (MET-hr/w) >=75.4 (vs 8.25-23.0) -> CLBP | 0.877+0.1499 0.864+0.1372 0.979+0.1566 1.021+0.1310 | None |  |  |  | T | F | T | N/A | T | T | T | T |
| Karjalainen 2013 ^60^ | CS | Finland | adolescents 18 | general public | structured questionnaire: "How often do you participate in moderate physical activity causing at least some shortness of breath and sweating outside school or working hours?" | (long-term) sciatic pain | structured questionnaire: 1. "Have you suffered from sciatic pain (1) ever? (2) during the past year? (3) during the past month? (4) during the past week? (5) today?" 2. 10-point NRS 3. "Have you ever suffered from long-term, continuous sciatic pain lasting during 2 weeks?" 4. other questions for level of physical impairment and related healthcare utilization | 1987 | None | aOR+SE | sex, LBP at 16 yr, BMI, smoking, sedentary behavior, physical workload | PA: 4-7/w*, 1-3/w, ≤2-3/mo sciatica: mild*, severe | 4-7/w PA (vs ≤2-3/mo) -> mild sciatica | All | 0.600+0.470 | 1-3/w PA (vs 4-7/w) -> mild sciatica 4-7/w PA -> severe sciatica 1-3/w PA (vs 4-7/w) -> severe sciatica | 1.406+0.410 2.766+0.631 1.060+0.420 | Oulu Back Study, a subcohort of the 1986 Northern Finland Birth Cohort |  |  |  | T | T | F | N/A | T | T | F | T |
| Karunanayake 2013 ^61^ | CC | Sri Lanka | M adults 47.8 ± 16 | general public | duration/day and frequency/w of exercise inquired by structured questionnaire | continuous LBP | clinical examination for pain confined to lumbar and sacral regions of the spine with or with-out radiation to lower limbs for more than 3 months duration | 362 | 0.889 | aOR+SE | education, family history of back pain, posture, alcohol consumption, animal protein consumption | exercise grade: 1 (<1/w), 2, 3*(>=3/w >=30min/d) LBP: no*, yes | exercise grade 3 (vs 1) -> LBP | All | 0.0609+0.9045 | exercise grade 2 (vs 3) -> LBP | 5.5463+0.5549 | None | 2 | 2 | 2 |  |  |  |  |  |  |  |  |
| Kędra 201 ^62^ | CS | Poland | young adults | students | structured questionnaire inquiring 1. Courses enrolled in Bachelor's degree 2. Session duration and weekly frequency of free time PA 3. Proportion by time of physical education (PE) classes of the course curriculum 4. Any training experience | Back pain | structured questionnaire inquiring 12mo-prevalence, frequency, location, intensity (10-point VAS) | 1131 | 0.106 | Raw counts | N/A | PA: inactive students (no training + no PE classes)*, untrained PE students (no training + 798hr/3y PE classes), trained PE students (5d/w 60min/d training + 798hr/3y PE classes) back pain incidence: 1-2/y, 3-6/y, >1-2/mo* | untrained PE students -> >1-2/mo back pain | All | 36, 179, 55, 465 | trained PE students -> >1-2/mo back pain | 35, 157, 55, 465 | None |  |  |  | T | F | F | N/A | F | F | F | T |
| Kemta Lekpa 2021 ^63^ | CS | Cameroon | children 11 ± 1 | students | structured questionnaire inquiring usual PA | LBP | structured questionnaire inquiring any pain or discomfort in the area located between the lower margin of the 12th ribs to the lower gluteal folds with or without pain referred into 1 or both lower limbs that lasts for at least 1day | 1075 | 0.0339 | aOR+SE | age, gender, parental LBP, weight of schoolbags, bad sitting posture | competitive sports: no*, yes LBP: no*, yes | competitive sports -> LBP | N/A | 1.6143+0.2293 | N/A | N/A | None |  |  |  | T | F | F | N/A | T | T | F | T |
| Kesiena 2021 ^64^ | CS | USA | adults | general public | PA assessment not documented | CLBP | structured questionnaire: "during the past three months, did you have low back pain?" | 25397 | 0.297 | Raw counts | None | PA: <150min/w, >150min/w CLBP: yes, no | PA >150min/w -> CLBP | N/A | 3467, 6466, 4595, 10869 | N/A | N/A | NHIS 2018 |  |  |  | T | T | F | N/A | F | F | F | T |
| Kim 2019 ^65^ | CH | USA | middle-aged and elderly ≥ 40 | general public | moderate-to-vigorous PA in leisure time in the past 30 days inquired during household interview | CP | miscellaneous pain questionnaire | 7384 | 0.156 | aOR+SE converted from aHR+SE | age, gender, sociodemographic characteristics, weight status, chronic health conditions, and unhealthy lifestyle behaviours. | PA: none, insufficient, sufficient chronic pain: localised, widespread | sufficient PA -> localised CP | all possible combinations | 0.37+0.3034 | insufficient PA -> localised CP sufficient PA -> widespread CP insufficient PA -> widespread CP | 0.80+0.2650 0.26+0.6709 0.60+0.5001 | NHANES 1999-2004 | 3 | 2 | 2 |  |  |  |  |  |  |  |  |
| Kirsch Micheletti 2019 ^66^ | CS | Denmark | adults 43.5 ± 11.7 | working population | structured questionnaire enquiring hr/w of leisure-time activities of low/moderate/high intensities, with examples given, in the past year | MSP (LBP, NSP) | 0-9 scale to the following question in a structured questionnaire: "Have you experienced any trouble (pain or discomfort) in your (body region) during the last 12 months?" | 10427 | LBP: 0.302 NSP: 0.316 | aRR+SE | alcohol, fruit and vegetable intake, smoking, age, gender, BMI, physical activity at work, psychosocial work factors (influence and emotional demands), job group, chronic disease (depression, cardiovascular, cancer) | physical exercise: 0hr/w*, 1-2hr/w, 3-4hr/w, >=5hr/w MSP: LBP*, NSP pain intensity: low (0-3.9)*, high (>=4) | 3-4hr/w physical exercise -> high LBP | All | 0.974+0.02356 | 1-2hr/w physical exercise -> high LBP >=5hr/w physical exercise -> high LBP 3-4hr/w physical exercise -> high NSP | 0.964+0.02116 0.946+0.02696 0.947+0.04307 | Danish Work Environment Cohort Study (DWECS) |  |  |  | T | F | F | N/A | T | T | F | T |
| Koneru 2015 ^67^ | CS | India | adults - elderly 30-70 | dentists | >1yr experience of >=4/w >=1hr/d physical activity and yoga assessed by structured questionnaire | work-related MSD | NMQ inquiring 12mo prevalence and 0-10 Pain Rating Scale | 220 M: 115 F: 105 | 0.456 | Raw counts | N/A | PA: no PA*, yoga activity, other PA pain: no*, severe, moderate, mild | yoga or other PA -> any pain | None | 14, 70, 62, 74 | N/A | N/A | None |  |  |  | T | T | F | N/A | F | F | T | T |
| Kopec 2017 ^68^ | CC | Canada | adults 41.24 ± 6.13 | general public | validated questionnaire inquiring occupation, domestic and recreational PAs (average level in lifetime). METs were calculated from the results | chronic pain in the hip joint (hip pain) | validated questionnaire inquiring any experience of pain, stiffness or discomfort in the left and right groin or the front of the upper thigh as well as duration and frequency of these symptoms | 500 | None | aOR+SE | age, gender, cam or pincer morphology | PA: all, occupational, domestic, recreational* hip pain: yes, no* | Recreational -> hip pain | All | 1.292+0.0728 | Domestic -> hip pain | 1.152+0.0903 | None | 2 | 2 | 2 |  |  |  |  |  |  |  |  |
| Kossi 2022 ^69^ | CS | Benin | adults 32.9 ± 13.1 | general public | structured questionnaire inquiring weekly frequency and duration of PA | CLBP | 0-10 NRS | 4320 | 0.347 | Raw counts | None | region: urban, rural PA: no regular PA, regular PA <150 min/w, regular PA ≥150 min/w CLBP: yes, no | urban+rural: regular PA ≥150 min/w -> CLBP | all possible combinations | 641, 1104, 854, 1604 | urban+rural: regular PA <150 min/w -> CLBP | 38, 79, 854, 1604 | None |  |  |  | T | T | F | N/A | T | F | F | T |
| Kovacs 2012 ^70^ | CC | Spain | adults 38-47 | general public | Unspecified validated questionnaire | CLBP | despite clinical records, duration and date of appearance of pain inquired by structured questionnaire. Also used were 0-10 VAS, Roland-Morris Disability Questionnaire | 304 | None | Raw counts | None | highly intense PA: no, yes CLBP: yes, no | highly intense PA -> CLBP | None | 51, 9, 148, 39 | N/A | N/A | None | 2 | 0 | 2 |  |  |  |  |  |  |  |  |
| Kumar 2017 ^71^ | CS | India | children 11.37 ± 3.15 | students | Children were inquired about indulgence in contact sports like football, wrestling, kabaddi, martial arts, etc., and its type and frequency | idiopathic MSP | Children were inquired about the presence, location, duration, frequency, character, description for each episode of MSP. Intensity was graded by 1-10 VAS. The information obtained was verified from the parents. Children with MSP duration <6w were excluded. | 1018 | 0.123 | Raw counts | N/A | contact sports: no*, yes idiopathic MSP: no, yes* | contact sports -> idiopathic MSP | N/A | 91, 253, 74, 600 | N/A | N/A | None |  |  |  | T | F | F | N/A | F | F | F | T |
| Landis 2004 ^72^ | CC | USA | F adults 45.0 ± 7.9 | general public | Three items from the Paffenbarger Physical Activity Questionnaire (number of stairs climbed, blocks walked, and time spent in vigorous activities) | fibromyalgia | fibromyalgia diagnosis from record linkage 1-10 self-rated pain score for severity | 70 | N/A | mean(N,SD) - mean(N,SD) | gender | PA: continuous (kcal/w) pain: fibromyalgia, control | PA: fibromyalgia - control | N/A | 615(33,628) - 725(37,412) | N/A | N/A | None | 3 | 0 | 1 |  |  |  |  |  |  |  |  |
| Landmark 2011 ^73^ | CS | Norway | adults 20-64 (45.71 ± 11.30) elderly >=65 | general public | validated structured questionnaire inquiring times/w frequency, average duration and average intensity each time of recreational exercise, e.g. going for a walk, skiing, swimming | CP | structured questionnaire inquiring | 20-64y: M: 19777, F: 16507 >=65y: M: 5347, F: 4902 | None | aPR+SE | age, smoking, education, exercise duration and intensity | age: 20-64y, >=65y gender: M, F exercise frequency: nonexercise (<1/w, <15min)*, 1/w, 2-3/w, >=4/w CP: yes*, no | 20-64y M: >=4/w exercise -> CP 20-64y F: >=4/w exercise -> CP >=65y M: >=4/w exercise -> CP >=65y F: >=4/w exercise -> CP | All | 1.0269+0.0397 1.0029+0.0330 0.7893+0.0613 0.6573+0.0465 | 20-64y M: 1/w exercise -> CP 20-64y F: 1/w exercise -> CP >=65y M: 1/w exercise -> CP >=65y F: 1/w exercise -> CP 20-64y M: 2-3/w exercise -> CP 20-64y F: 2-3/w exercise -> CP >=65y M: 2-3/w exercise -> CP >=65y F: 2-3/w exercise -> CP | 0.9027+0.0367 0.9133+0.0307 0.90 (0.79-0.95) 0.7613+0.0502 0.8833+0.0317 0.8979+0.0256 0.7301+0.0593 0.7275+0.0420 | HUNT3 |  |  |  | T | T | T | N/A | T | T | T | T |
| Lee 2005 ^74^ | CS | Switzerland | adults 39.11 ± 11.04 | employees of 2 large Swiss enterprises | structured questionnaire inquiring whether participants performed LTPA at least once a week and on how many days a week (running, cycling, aerobics, etc.) | BP | structured questionnaire inquiring whether, in the previous 4 weeks, participants had suffered from back pain: "not at all", "a little" or "severely" | 10321 | 0.519 | aOR+SE | age, gender, hierarchy , stress, obesity , upper body strength, abdominal musculature, smoking | PA (d/w): 0, 1, 2, 3, 4, 5, 6, 7* BP: any intensity, severe | 7d/w PA (vs none) -> BP of any intensity | All | 0.9616+0.3497 | 7d/w PA (vs none) -> severe BP | 1.2526+0.7460 | Check Bus Project |  |  |  | T | T | F | N/A | T | T | F | T |
| Lewandowski 2014 ^75^ | CS | Poland | adolescents 16.5 ± 1.5 | students | Structured questionnaire inquiring determinants related to lifestyle correlating with SP occurrence | spinal pain (SP) | the prevalence, characteristics, and circumstances of occurrence of SP were assessed, as well as the functional consequences of the occurrence of SP | 2095 | 0.373 | Raw counts | N/A | population: village residents, city residents, all* additional PA: none, >4hr/w SP: none, 1 episode in life, several episodes a year, several episodes a month, several episodes a week | all: >4hr/w additional PA -> SP several episodes each week - each month | All | 110, 194, 62, 104 | None | N/A | None |  |  |  | T | T | F | N/A | F | F | F | T |
| Li 2022 ^76^ | CS | China | adults 18-45 | general public | exercising hours per week inquired by structured interview | LBP | 0-10 VAS for pain intensity within the last 12 months 1-3 self-reported reliability score of VAS VAS * reliability / 3 ≥ 1 is considered having LBP | 264 | N/A | aOR+SE | working posture Tuffier's line  lumbosacral transitional vertebra sacral slope lumbar lordosis angle | exercise (hr/w): ≤0.5*, 0.5-1.5, 1.5-3, >3 LBP: yes, no | >3hr/w exercise -> LBP | all possible combinations | 0.275+0.618 | 0.5-1.5hr/w exercise -> LBP 1.5-3hr/w exercise -> LBP | 0.416+0.589 0.278+0.599 | None |  |  |  | T | F | F | N/A | T | T | T | T |
| Lilje 2015 ^77^ | CS | Sweden | middle-aged - elderly 68.35 ± 6.54 | general public | Physical leisure activity inquired by structured questionnaire: "For lei-sure, do you normally, during the last 12 months or earlier: (a) do garden work, (b) pick mushrooms, (c) walk in the forest, or (d) go hunting or fishing?" | MSP interfering with normal life | EuroQol 5 Dimensions (EQ5D) and "Have you experienced ache/pain during the last four weeks?" in structured questionnaire | 641 | 0.284 | aOR+SE | psychosocial workload , physical workload, age, gender, growing up environment, living alone, educational level, smoking, obesity | Physical leisure activities: yes*, no MSP interfering with normal life: yes*, no | Physical leisure activities -> MSP interfering with normal life | None | 0.3842+0.3868 | None | N/A | Swedish National Study on Aging and Care (SNAC) |  |  |  | T | T | F | N/A | T | T | T | T |
| López-Bueno 2020 ^78^ | CS | Spain | adults 44.4 ± 10.4 | general public | IPAQ-SF | CLBP | structured questionnaire: "Have you suffered from chronic low-back pain within the last 12 months?" | 9695 | N/A | aOR+SE | sex, age, education, BMI, smoking, occupational class, fruit consumption , occupational PA | PA (MET-min/w): <600, >=600 LBP: yes*, no | PA >=600 MET-min/w -> LBP | N/A | 0.7981+0.0669 | N/A | N/A | Spanish National Health Survey (ENSE) 2017 |  |  |  | T | T | T | N/A | T | T | F | T |
| Macedo 2022 ^79^ | CS | Brazil | adults ≥ 18 | students and professros | practice, level (sedentary or active) and time spent in physical activity inquired by structured online questionnaire | MSP | presence of pain, region, and 0-10 NRS for intensity during and before pandemic | 1254 | 0.552 | aPR+SE | sex, income, physical activity before the pandemic, general physical activity level, sedentary behavior during the pandemic, electronic device usage before the pandemic, electronic device usage during the pandemic | physical activity during the pandemic: yes, no increased perceived pain during the pandemic: yes, no | no physical activity during the pandemic -> increased perceived pain during the pandemic | N/A | 1.22+0.0710 | N/A | N/A | None |  |  |  | T | F | F | N/A | T | T | F | T |
| Macfarlane 2010 ^80^ | CH | UK | adults ≥ 25 | general public | Structured questionnaire: "During the past month, on average, on how many days per week have you taken exercise that has lasted at least 20 minutes?" | chronic widespread pain | structured questionnaire inquiring presence, site(s), and duration of pain | 2491 | 0.252 | PR+SE | N/A | exercise frequency: every day*, 4-6d/w, 2-3d/w, 1d/w, none CWP today: yes, no | no exercise (vs every day) -> CWP | 4-6d/w exercise (vs every day) -> CWP 2-3d/w exercise (vs every day) -> CWP 1d/w exercise (vs every day) -> CWP | 1.74+0.2523 | 4-6d/w exercise (vs every day) -> CWP 2-3d/w exercise (vs every day) -> CWP 1d/w exercise (vs every day) -> CWP | 0.78+0.3101 0.76+0.0502 1.41+0.1537 | EpiFunD study |  |  |  | T | T | F | N/A | T | F | F | T |
| Markkula 2016 ^81^ | CH | Finland | adults 27.7 ± 7.3 | general public | structured questionnaire inquiring year-round LTPA level as a choice from 1) "I do not exercise in my leisure time practically at all", 2) "a bit", 3) "fairly", 4) "fairly much" and 5) "much", and another questionnaire inquiring monthly exercising frequency | fibromyalgia | fibromyalgia symptom questionnaire | 8343 | N/A | OR+SE | age, gender | cohort: 1975, 1981 LTPA: passive, moderate*, active  pain: frequent FM symptoms resembling clinical FM patients*, some FM symptoms | 1981: passive LTPA (vs moderate) -> frequent FM symptoms resembling clinical FM patients | all possible combinations | 1.08+0.1383 | 1975: passive LTPA (vs moderate) -> frequent FM symptoms resembling clinical FM patients | 1.15+0.1098 | Finnish Twin Cohort | 4 | 2 | 3 |  |  |  |  |  |  |  |  |
| McLoughlin 2011 ^82^ | CC | USA | F adults 41.7 ± 10.7 | general public | IPAQ-LF | FM | letter from patient's own healthcare provider confirming FM diagnosis. McGill Pain Questionnaire short for, Fibromyalgia Impact Questionnaire | 79 | N/A | mean(N,SD) - mean(N,SD) | depression, substance abuse, usage of analgesic, cardiovascular, or high-dose antidepressant medications | LTPA: recreation (continuous MET) pain: FM, control | recreation LTPA: FM vs control | N/A | 443(39,743) - 1183(40,1300) | N/A | N/A | None | 2 | 0 | 2 |  |  |  |  |  |  |  |  |
| McLoughlin 2011b ^83^ | CC | USA | adults 41.8 ± 10.7 | general public | IPAQ-LF | FM | letter from patient's own healthcare provider confirming FM diagnosis. | 34 | N/A | mean(N,SD) - mean(N,SD) | usage of analgesic, cardiovascular, or high-dose antidepressant medications, recent structured exercise, recent alcohol consumption, recent caffeine consumption, recent smoking | LTPA: recreation (continuous MET) pain: FM, control | recreation LTPA: FM vs control | N/A | 581(16,1064) - 1181(18,1369) | N/A | N/A | None | 2 | 0 | 2 |  |  |  |  |  |  |  |  |
| Meucci 2013 ^84^ | CS | Brazil | adults 45.89 ± 16.18 | general public | IPAQ-SF | CLBP | NMQ with adapted body region diagram and "In the last three months, have you felt this pain for seven weeks or more(50 days) continuously?" for reported LBP | 2732 | None | aRR+SE | sex, age, skin color, civil status, education (years), smoking, BMI, repetitiveness , weight lifting/loading, forced/awkward position, static posture | insufficient PA: no*, yes CLBP: yes*, no | sufficient PA (vs insufficient PA) -> CLBP | None | 0.9771+0.1689 | None | None |  |  |  |  | T | T | T | N/A | T | T | F | T |
| Mikkelsson 1998 ^85^ | CH | Finland | children 9.8 ± 0.34 | students | structured questionnaire inquire weekly frequency of exercising to shortness of breath at least half an hour | MSP | previously validated questionnaire inquiring pain during the previous 3mo >=1/w in >=1 part of the body, excluding pain due to injuries | 452 | N/A | aOR+SE | sex, age, symptoms at least once a week: headache, abdominal pain, depressive feelings, difficulties in falling asleep, day tiredness, waking up during nights, minor criteria for fibromyalgia, disability index, Beighton score for hypermobility | amount exercise: 0*, 1-2, 3-4, 5-7 persistence of MSP >=1/w: yes*, no | 3-4/week exercise -> persistence of MSP | None | 1.1575+0.3525 | 1-2/week exercise -> persistence of MSP 5-7/week exercise -> persistence of MSP | 1.1424+0.3458 2.2450+0.4126 | None | 4 | 2 | 3 |  |  |  |  |  |  |  |  |
| Miranda 2001 ^86^ | CH | Finland | adults 45.3 ± 9.13 | employees of a large forestry company | Number of times each of 15 listed sport was practised every year inquired by structured questionnaire | shoulder pain | modified NMQ: "Estimate the total number of days you have had pain during the preceding 12 months?" | 3312 | None | aOR+SE | age, sex, cross country skiing, overload at work, working with a hand above shoulder level | Sports activity added score: 0-52 (<=1/w)*, 53-156 (1-3/w)*, >156 (>3/w) shoulder pain: incident, persistent severe (>30d/y)* | 1-3/w sports activity -> persistent severe shoulder pain | All | 0.4517+0.4055 | >3/w sports activity -> persistent severe shoulder pain | 0.7211+0.3007 | None | 4 | 2 | 2 |  |  |  |  |  |  |  |  |
| Mork 2010 ^87^ | CH | Norway | F adults 43.2 ± 13.9 | general public | structured questionnaire inquiring weekly frequency, duration, and intensity of leisure time physical exercise | FM | Structured questionnaire: "During the last year, have you had pain and/or stiffness in your muscles and limbs that lasted for at least 3 consecutive months?" and duration of symptom. | 15990 | 0.028 | aRR+SE | age, smoking status, psychological well-being, education, BMI | exercise (summary score): inactive*, low, medium, high exercise (h/w): inactive*, <1.0, 1.0-1.9, ≥2.0 FM: yes, no | medium exercise -> FM | all possible combinations | 0.90+0.1449 | low exercise score -> FM high exercise score -> FM <1.0 h/w exercise -> FM 1.0-1.9 h/w exercise -> FM ≥2.0 h/w exercise -> FM | 1.03+0.1281 0.77+0.1698 1.00+0.1211 0.87+0.1439 0.77+0.2025 | HUNT1-HUNT2 | 4 | 2 | 3 |  |  |  |  |  |  |  |  |
| Mork 2014 ^88^ | CH | Norway | adults | general public | structured questionnaire inquiring the frequency, duration and intensity of leisure time physical exercise per week | CMSP | structured questionnaire adapted from NMQ: "During the last year, have you had pain and/or stiffness in your muscles and limbs that lasted for at least three consecutive months?" Affected body area(s) were also inquired. | 26896 M: 13395 F: 13501 | 0.156 | aRR+SE | age, BMI, psychological well-being, smoking status, occupation, frequency of sleep problems | baseline leisure time physical exercise: active, inactive* frequency of sleep problems: never*, sometimes, always pain location: low back*, neck/shoulders | without sleep problems: active -> low back pain | All | 0.8818+0.0433 | Without sleep problems: active -> neck/shoulders pain | 0.8472+0.0331 | HUNT1-HUNT2 | 4 | 2 | 2 |  |  |  |  |  |  |  |  |
| Myrtveit 2014 ^89^ | CS | Norway | adolescents 17.85 ± 0.7934 | general public | PA inquired by structured questionnaire as number of days during the last week being physically active for more than 60 minutes | NSP | frequency of suffering from neck and shoulder pain during the last 6 months inquired by structured questionnaire | 8990 M: 4159 F: 4831 | None | aOR+SE | age, school situation, family economy, symptoms of depression | M, F PA: 0d/w*, 1-3d/w, 4-7d/w NSP: frequent ("more than once a week" or "more or less every day")*, rarely ("every week", "every month" or "seldom/never") | M, F: 1-3d/w PA -> frequent NSP | M, F: 4-7d/w PA -> frequent NSP | M: 0.5477+0.1604 F: 0.7063+0.1005 | M, F: 4-7d/w PA -> frequent NSP | 0.5444+0.1702 0.6712+0.1205 | ung@hordaland-survey |  |  |  | T | F | F | N/A | T | T | T | T |
| Nah 2022 ^90^ | CS | Korea | elderly | general public | hr/w of aerobic PA inquired by structured questionnaire | CLBP | structured questionnaire: "Did you experience low back pain for more than 30 days in the last 3 months?" | 5233 | 0.3195 | Raw counts aOR+SE | age, sex, body weight status, hours of sleep, smoking status, alcohol consumption, education, occupation status, household income, depressive symptoms, aerobic physical activity, co-morbidities | PA: aerobic*, walking, resistance training CLBP: yes*, no walking/resistance training frequency: none*, 1-2d/w, 3-4d/w, >=5d/w | aerobic PA -> CLBP | other PA at all frequencies -> CLBP | 274, 834, 1318, 2807 | 3-4d/w walking -> CLBP 3-4d/w resistance training -> CLBP | 0.6893+0.1246 1.0321+0.2205 | KNHANES 2012-2015 |  |  |  | T | T | F | N/A | T | T | F | T |
| Natvig 1998 ^91^ | CC | Norway | F adults 30-70 years | general public | structured questionnaire: `How much do you use your body (so hard that you sweat and/or breathe heavily) in your leisure time?' with choices of hours per week | FM | record linkage, self-reported diagnosis of FM, and structured questionnaire: ''During the past two weeks... How much bodily pain have you generally had?', and NMQ | 1043 | 0.234 | aOR+SE | age, civil status, pain characteristics (intensity, duration, widespread-ness), employment status, hours of salaried work a day, hours of total work a day, subjective work load, sleep quality, BMI | LTPA: >2hr/w, ≤2hr/w FM: yes, no | >2hr/w LTPA (vs ≤2hr/w) -> FM | N/A | 1.98+0.2276 | N/A | N/A | None | 2 | 2 | 3 |  |  |  |  |  |  |  |  |
| Nilsen 2011 ^92^ | CH | Norway | adults 44.05 ± 14.01 | general public | frequency, duration and intensity of leisure-time physical exercise (i.e., walking, skiing, swimming, or other sports) per week inquired by structured questionnaire | CMSP | modified NMQ inquiring "During the last year, have you had pain and/or stiffness in your muscles and limbs that lasted for at least 3 consecutive months?"" and affected body areas(s) | 32417 M: 15465 F: 16952 | LBP: 0.111 (M: 0.107, F: 0.116) NSP: 0.209 (M: 0.192, F: 0.226) | M: aOR+SE, F: aOR+SE | age, BMI, smoking, occupation | gender: All, M*, F* exercise: inactive (<1/w)*, <1hr/w, 1-1.9hr/w, >=2hr/w CP: LBP*, NSP | M, F: >=2hr/w exercise -> CLBP | All | M: 0.7504+0.0812, F: 0.9194+0.0774 | M, F: <1hr/w exercise -> CLBP M, F: 1-1.9hr/w exercise -> CLBP M, F: >=2hr/w exercise -> CNSP | M: 0.9078+0.0645, F: 0.9045+0.0563 M: 0.8774+0.0666, F: 0.8385+0.0637 M: 0.8094+0.0597, F: 0.9085+0.0560 | HUNT1-HUNT2 | 4 | 2 | 2 |  |  |  |  |  |  |  |  |
| Noormohammadpour 2016 ^93^ | CS | Iran | adolescents - elderly 44.37 ± 16.79 | general public | Iranian version of GPAQ | LBP | structured questionnaire: 1. "Did you have LBP during past 48hours?" 2. "Did you experience LBP during the last year?" 3. "Did you experience LBP for more than 3 months over the past 6 months?" 4. "Did you visit your physician because of your LBP during the last year?" 5. "How many days were you absent from work due to your LBP during the last year?" | 22952 | 0.131 | aOR+SE | age, sex, BMI, marital status, education, employment status, smoking, general health | PA: low, moderate*, high LBP: current, 1y prevalence, CP* | moderate PA (vs low) -> CLBP | All | 1.0712+0.0600 | high PA (vs moderate) -> CLBP | 0.9930+0.0793 | None |  |  |  | T | T | T | N/A | T | T | F | T |
| Noormohammadpour 2017 ^94^ | CS | Iran | adults 50.86 ± 13.08 | general public | Persian version of GPAQ | CLBP, CNP, CKP | structured questionnaire inquiring for any experience of low back, neck, or knee, pain which lasts for more than 3 months during the last year | 7889 | None | aOR+SE | age, sex, BMI, ethnicity, living area, education, employment status, smoking, osteoporosis history | PA: low, moderate*, high CP: CLBP, CNP, CKP | moderate PA (vs low) -> CLBP | All | 0.7767+0.0711 | high PA (vs moderate) -> CLBP moderate PA (vs low) -> CNP | 1.3463+0.0849 0.7867+0.0879 | None |  |  |  | T | T | T | N/A | T | T | F | T |
| O'Sullivan 2011 ^95^ | CC | Australia | children - adolescents 12.7 (7-18) | general public | Youth Activity Questionnaire | chronic non-specific MSP: pain present for >3d/w on average for >3mo usually associated with interference with or modification of normal function | medical diagnosis by a paediatric rheumatologist with clinical and radiological evidence. | 30 M: 12 F: 18 | None | mean(n,SD) - mean(n,SD) | age, sex | PA level: continuous pain: yes, no | pain group vs control group: PA level | N/A | 1.3(30,1.29) - 2.83(30,1.26) | N/A | N/A | None | 2 | 2 | 3 |  |  |  |  |  |  |  |  |
| Palmlöf 2016 ^96^ | CH | Sweden | adults 43.76 ± 10.91 | working population | LTPA inquired by structured questionnaire: "How much have you been physically active in your leisure time during the past twelve months?", with averaging across seasonal variations | long duration troublesome neck pain | structured questionnaire: "During the last five-year period, have you had neck pain for at least three consecutive months that bothered you considerably?" | 4681 | 0.034 | aOR+SE | age, smoking , alcohol consumption, immigrant status, work above shoulder level , work under knee level, computer work, psychological distress, individual disposable income | sex: both*, M, F LTPA: sedentary*, moderate (>2hr/w without perspiring), moderate regular or high regular long duration troublesome NP: yes*, no | both sexes: moderate regular or high regular LTPA -> NP | All | 0.6000+0.2069 | both sexes: moderate LTPA -> NP M: moderate regular or high regular LTPA -> NP F: moderate regular or high regular LTPA -> NP | 0.6633+0.2094 0.6000+0.3536 0.5745+0.3314 | Stockholm Public Health Cohort | 4 | 2 | 2 |  |  |  |  |  |  |  |  |
| Park 2018 ^97^ | CS | Korea | middle-aged - elderly 64.29 - 8.94 | general public | IPAQ | CLBP | structured questionnaire: "have you complained of LBP for more than 30 days during the past 3 months?" | 5364 | 0.256 | Raw counts | None | PA: yes, no CLBP: yes, no | PA -> CLBP | All | 454, 1911, 768, 2231 | None | None | KNHANES 2014-2015 |  |  |  | T | T | T | N/A | F | F | T | T |
| Payne 2000 ^98^ | CS | Canada | adolescents - middle-aged 34.58 ± 15.33 | general public | validated Healthy Physical Activity Participation Questionnaire inquiring frequency and intensity of activity and perceived fitness | history of back pain | structured questionnaire: "Do you suffer from chronic (recurring) back pain?" | 520 M: 233 F: 287 | None | mean(N,SD) - mean(N,SD) | None | gender: M, F history of back pain: yes, no PA participation score: continuous | M, F: PA participation score -> history of back pain | N/A | M: 7.30(25,2.482) - 8.41(188,2.468) F: 6.20(67,2.865) - 7.02(220,2.225) | N/A | N/A | None |  |  |  | F | F | F | N/A | T | T | F | T |
| Peltzer 2022 ^99^ | CH | South Africa | middle-aged and elderly ≥ 40 | general public | GPAQ | persistent intrusive pain | BPI: "Now I have some questions about pain. Throughout our lives, most of us have had pain from time to time (such as minor headaches, sprains and toothaches). Have you had pain‚ other than these everyday kinds of pain today?" | 5059 | 0.027 | aOR+SE | age, sedentary behaviour, general body weight | PA: low*, moderate, high persistent intrusive pain: yes, no | high PA -> persistent intrusive pain | moderate PA -> persistent intrusive pain | 0.53+0.3910 | moderate PA -> persistent intrusive pain | 0.95+0.3282 | HAALSI 2014-2015, 2018-2019 | 4 | 2 | 2 |  |  |  |  |  |  |  |  |
| Peng 2018 ^100^ | CS | USA | adults 47.0 ± 35.81 | general public | structured questionnaire inquiring the frequency and duration of prior activity within intensity categories: light, moderate, and vigorous of LTPA | LBP | structured questionnaire: "During the past 3 months, did you have low back pain?" | 32060 | 0.362 | aOR+SE | age, sex, race/ethnicity, BMI, highest level of education | LTPA: inactive (0 min/w), insufficiently active (<150 min/w), sufficiently active (>=150 min/w)* LBP: yes*, no | sufficiently active (vs inactive) -> LBP | insufficiently active (vs sufficiently active) -> LBP | 0.6946+0.0442 | insufficiently active (vs sufficiently active) -> LBP | 1.2351+0.0454 | NHIS 2015 |  |  |  | T | T | F | N/A | T | T | F | T |
| Picavet 2021 ^101^ | CH | Netherlands | adolescents 11-20 | general public | structured questionnaire inquiring physically active min/d and d/w. Moderately active is ≥60min/d ≥5d/w, while vigorously active is being physically active with heavy breathing and sweating for >=30min/d >=2d/w. | MSCs | structured questionnaire: "Please indicate whether you experienced one of the following health problems in the past 12 months". Relevant items include: "long-lasting back complaints", "long-lasting complaints of the upper extremities (neck, shoulder, elbow, wrist or hand)", and "long-lasting complaints of the lower extremities (hip, knee, ankle or foot)". "long-lasting" refers to longer than one month. | 1895 | None | aOR+SE | sex, level of education of the adolescent, level of education of the mother, early puberty, attained height at 20y, overweight, mental health status, worrying, being bullied, sleeping problems, daytime tiredness/sleepiness, hyperactivity, screen time, smoking, accidents | gender: both*, M, F PA: moderate, vigorous persistent pain: yes*, no | both genders: vigorous PA -> persistent pain | All | 0.7680+0.2251 | both genders: moderate PA -> persistent pain M: vigorous PA -> persistent pain F: vigorous PA -> persistent pain | 0.8683+0.2059 1.0894+0.4399 0.7010+0.2614 | PIAMA birth cohort study | 3 | 2 | 2 |  |  |  |  |  |  |  |  |
| Queiroz 2018 ^102^ | CS | Brazil | adolescents 10-19 | students | structured questionnaire inquiring participation in physical education classes at school and regular practice of any other sport | MSP and MSP syndromes | questionnaire: "Did you have any pain on muscles, bones, or joints in the past three months?", followed by physical examination of the musculoskeletal system by trained physicians | 331 | 0.702 | Raw counts | N/A | PA: yes, no MSP: yes, no MSP syndromes: any of the following, juvenile fibromyalgia, benign joint hypermobility syndrome, myofascial syndrome, tendinitis, bursitis, epicondylitis, complex regional pain syndrome | PA -> MSP | None | 150, 102, 33, 14 | N/A | N/A | No significant association was found between PA and MSP syndromes (p>0.05) |  |  |  | T | T | F | N/A | T | F | T | T |
| Rashiq 2009 ^103^ | CS | Canadia | young adults - elderly | general public | structured questionnaire inquiring any recreational PA in the past 3mo | chronic noncancer pain | structured questionnaire:  1. "Are you usually free of pain and discomfort?" 2. "How would you describe the usual intensity of your pain or discomfort?" ("mild", "moderate" or "severe") | 69365 | 24.3 | aOR+SE | age, sex, civil status, annual household income, highest education, white race, arthritis, back problems, other chronic medical condition, bowel disorder, migraines, epilepsy, COPD, heart disease, stroke, ulcers, diabetes, sinusitis, hypertension, thyroid disorder, depression, alcohol abstainer, cigarette smoking, 12mo activity-limiting injury, spirituality/faith important | PA: inactive, active* chronic noncancer pain: yes*, no | active (vs inactive) -> chronic noncancer pain | None | 0.5552+0.0340 | N/A | N/A | Canadian NPHS 1996/1997 |  |  |  | T | T | F | N/A | T | T | F | T |
| Ray 2022 ^104^ | CS | US | adults 52.3 ± 17.3 | general public | self-reported adherence to current PA guidelines assessed by structured questionnaire | CP | Structured questionnaire inquiring frequency and intensity of pain in the past 3 months | 31568 | N/A | OR+SE | N/A | PA (meeting guideline): meets strength only, meets aerobic only, meets both criteria, meets neither criteria CP: never, some days, most days, every day, reduced pain frequency | most days having pain (vs never) -> engaging any PA | PA meeting both criteria (vs meeting none) -> less frequent pain PA meeting aerobic criteria (vs meeting none) -> less frequent pain PA meeting strength criteria (vs meeting none) -> less frequent pain | 0.497+0.0415 | PA meeting both criteria (vs meeting none) -> less frequent pain PA meeting aerobic criteria (vs meeting none) -> less frequent pain PA meeting strength criteria (vs meeting none) -> less frequent pain | 2.274+0.0283 1.632+0.0278 1.381+0.0450 | NHIIS 2020 |  |  |  | T | T | T | N/A | T | T | F | T |
| Rodriguez-Nogueira 2021 ^105^ | CS | Spain | adults 46.42 ± 11.15 | university employees | frequency [never, occasionally (some days a month), frequently (seven days a week)] and type [nothing, aerobic, strength exercises, another type (e.g. stretching)] of PA done inquired by structured questionnaire: 1. "Did you do PA before the confinement?" 2. "Did you do any PA during the confinement?" 3. "What typeof PA did you mainly do before the confinement?" 4. "What type of PA did you mainly do during the confinement?" | MSP | NMQ | 472 M: 189 F: 283 | None | aOR+SE | sex, age, perception sadness or anxiety, self-perceived stress, amount of sitting down per day | frequency of PA during confinement: continuous MSP during confinement: yes*, no | Higher frequency of PA during confinement -> MSP during confinement | N/A | 0.914+0.173 | N/A | N/A | None |  |  |  | T | F | F | N/A | T | T | T | T |
| Romero 2018 ^106^ | CS | Brazil | adults - elderly | general public | PA assessment not documented | chronic back problems | structured questionnaire: "Do you have some chronic back problem, like chronic pain in your back or neck, lumbago, sciatica, or problems in the vertebrae or discs?" | 60202 | 0.16 | aPR+SE | sex, age, schooling, area of residence, race/color, self-rated health, hypertension, depression, arthritis, diabetes, asthma, chronic heart disease, BMI | age group: total*, 18-49y, 50-59y, >=60y PA: yes*, no chronic back problems: yes*, no | Total age group: PA (vs no) -> chronic back problems | All | 1.0437+0.0319 | 18-49y: PA (vs no) -> chronic back problems 50-59y: PA (vs no) -> chronic back problems >=60y: PA (vs no) -> chronic back problems | 1.0096+0.0489 1.1220+0.0713 1.1317+0.0636 | Brazilian National Health Survey 2013 |  |  |  | T | T | F | N/A | T | T | F | T |
| Ryan 2012 ^107^ | CS | UK | middle-aged 54.7 ± 5.8 elderly 73.8 ± 6.5 | general public | 1. individual interview interviewing weekly duration, frequency and intensity of PA 2. 1w consecutive accelerometry for randomly selected sample | CMSP | structured questionnaire inquiring any long-standing illness, any listed musculoskeletal system condition, and any pain (none, moderate, severe) on the day of completing the questionnaire | middle-aged: 3332 (715 with accelerometry) elderly: 2022 (492 with accelerometry) | middle-aged: 0.214 elderly: 0.308 | Raw counts mean(n,SD) - mean(n, SD) | N/A | PA: subjective PA guidelines achieved, objective MVPA min/d CMSP: yes, no | subjective PA guidelines achieved -> CMSP | CMSP - no CMSP: objective MVPA min/d | middle-aged: 137, 1036, 462, 1697 elderly: 51, 275, 522, 1174 | CMSP - no CMSP: objective MVPA min/d | middle-aged: 23.6(599,21.1) - 32.1(2733,25.2) elderly: 11.0(573,14.5) - 17.4(1449,22.1) | HSE 2008 |  |  |  | T | T | T | N/A | F | F | T | T |
| Salminen 1993 ^108^ | CC | Finland | adolescents 15 | students | structured interview inquiring type, frequency and duration of sport activities. | continuous or recurrent LBP | structured questionnaire inquiring experience of LBP, defined exactly in relation to time and localization as a pain, ache or uncomfortable feeling at a location demonstrated by a drawing | 38 | 0.636 | Raw counts | age, sex, school | M, F PA: active (>2d/w active), inactive (0-2d/w active) LBP: yes, no | M, F: PA -> LBP | N/A | 10, 22, 28, 16 M: 6, 11, 11, 6  F: 4, 11, 17, 10 | N/A | N/A | None | 4 | 2 | 3 |  |  |  |  |  |  |  |  |
| Santos 2018 ^109^ | CS | Brazil | adults 42.54 ± 11.52 | teachers | structured interview inquiring whether some type of leisure time PA is performed in a typical week. If so, the type of activity, the weekly frequency, and the duration in minutes are described. | CP | structured interview: 1. "Do you suffer from any type of CP that has affected you for 6 months or more?" 2. "Please point to the part of the body where you feel this pain." with a figure of human body provided | 943 | 0.343 | aOR+SE | sex, age, BMI, smoking, alcohol consumption, sleep quality, time spent watching television and depression, discipline, number of shifts, time spent sitting, time spent standing, work load, contract type, perception of work-life balance | LTPA: inactive*, insufficient, sufficient CP: yes*, no | sufficient LTPA -> CP | insufficient LTPA -> CP | 1.1764+0.2365 | insufficient LTPA -> CP | 0.9687+0.1805 | PRO-MESTRE project baseline |  |  |  | T | T | F | N/A | T | T | F | T |
| Santos 2020 ^110^ | CH | Brazil | adults 42 | teachers | LTPA assessed by structured questionnaire : 1. "In a normal week (typical), do you do some type of physical activity in the free time at least once a week?" 2. "How many times a week do you practice physical activity (days)?" 3. "How much time per day do you practice physical activity?" | CMSP | structured questionnaire: 1. "Do you suffer from any type of chronic pain that has affected you for 6 months or more?" 2. "Please point to the region of the body where you feel this pain." with a figure of human body provided | 527 | None | aOR+SE | change in TV-viewing time, sex, age, change in BMI, change in depression status | change in LTPA time: continuous (per 60 min/w)* CMSP: yes*, no | change in LTPA time -> CMSP | N/A | 0.9380+0.0288 | N/A | N/A | Pro-Mestre study | 4 | 2 | 2 |  |  |  |  |  |  |  |  |
| Shiri 2013 ^111^ | CH | Finland | adults 31.4 ± 5 | adults | LTPA inquiring intensity and average session duration of PA, frequency and duration of vigorous PA, and participation in organised PA. Total MET (kcal/kg/h) calculated and dichotomised by mean. Changes less than 0.5 gender-specific SD are considered insignificant. | LBP | structured questionnaire: 1. "Have you had low back trouble(pain, ache, or unpleasant sensations) during the preceding 12 months?" with manikin to denote the anatomic area 2. "What is the total length of time you have had low back trouble radiating below the knee during the preceding 12 months?" 3. "What is the total length of time you have had low back trouble other than radiating pain during the preceding 12 months?" | 1224 | None | aOR+SE | age, gender, educational status, occupational status, smoking | gender: both*, M, F average PA: low, moderate*, high LBP: non-specific, radiating | both: moderate average PA (vs low) -> non-specific LBP | All | 1.3484+0.2011 | both: moderate average PA (vs low) -> radiating LBP both: high average PA (vs moderate) -> non-specific LBP both: high average PA (vs moderate) -> radiating LBP M: moderate average PA (vs low) -> non-specific LBP M: moderate average PA (vs low) -> radiating LBP F: moderate average PA (vs low) -> non-specific LBP F: moderate average PA (vs low) -> radiating LBP | 0.6521+0.2285 1.1314+0.1768 1.1533+0.2546 1.1952+0.2627 0.8704+0.3314 1.7408+0.3314 0.5000+0.3536 | Young Finns Study 2001-2007 follow-up | 4 | 2 | 2 |  |  |  |  |  |  |  |  |
| Shiri 2019 ^112^ | CH | Finland | adults - elderly | general public | structured questionnaire inquiring the type, intensity, weekly duration and frequency of LTPA | LBP | structured questionnaire:  1. "Have you had back pain in the past 12 months?" 2. "Have you had back pain that radiates down the leg, beyond the knee in the past 12 months?" 3. Positive responses to previous question(s) followed by modified NMQ inquiring d/12mo with specified pains | 3505 | None | aOR+SE | age, sex | LTPA: low*, moderate, high pain (d/y): LBP >7d/y, LBP >=30d/y, lumbar radicular pain >7d/y, lumbar radicular pain >=30d/y | moderate LTPA -> LBP >=30d/y | All | 0.8552+0.1095 | high LTPA -> LBP >=30d/y | 0.6983+0.1407 | Finland Health 2000 Survey | 4 | 2 | 2 |  |  |  |  |  |  |  |  |
| Shiri 2020 ^113^ | CH | Finland | middle-aged 40-60 | public servants | structured questionnaire inquiring the average weekly duration (hr) of LTPA within the past 12mo at 4 intensity levels: walking, brisk walking, jogging, and running, equivalent. Total MET-hr/w is calculated | incident (cross-sectional)/persistent (longitudinal) CP | structured questionnaire:  1. "Do you have any pain or ache right now?" 2. "When did the pain start?" | 18562 | 0.357 | aOR+SE | age, gender, education, smoking, BMI | gender: both*, M, F LTPA: low (<14 MET-hr/w)*, moderate (>=14 MET-hr/w + no vigorous vigorous activities), vigorous (>= 14 MET-hr/w + some vigorous activity, e.g. jogging or running) CP: incident, persistent | both genders: moderate LTPA -> incident CP | All | 0.9637+0.0581 | both genders: vigorous LTPA -> incident CP M: moderate LTPA -> incident CP F: moderate LTPA -> incident CP | 0.8485+0.0630 0.9675+0.1507 0.9569+0.0665 | Finnish Helsinki Health Study |  |  |  | T | T | F | N/A | T | T | F | T |
| Silva 2017 ^114^ | CS | Portugal | adolescents 13-19 | students | validated structured questionnaire inquiring any participation in MVPA, and the type, d/w frequency and mean duration per session if any. | CP | adapted Portuguese version NMQ inquiring any pain at least once a week during the last 3 months for each of the listed body regions. | 969 | None | aOR+SE | gender, age, BMI, sleeping hours, time spent watching TV/DVDs, time spent using mobile phones, time spent using computers | PA: moderate (yes/no), moderate (hr), vigorous (yes/no), vigorous (hr) CP: neck, mid back, lower back, shoulder, elbow, wrist, hips, knees, ankle/feet | PA moderate (hours) -> CLBP | All | 1.0588+0.0241 | PA vigorous (hours) -> CLBP | 1.0388+0.0245 | None |  |  |  | T | T | F | N/A | T | T | F | T |
| Skarpsno 2019 ^115^ | CH | Norway | F adult 44.1 ± 12.0 | general public | structured questionnaire: 'How much of your leisure time have you been physically active during the last year? (Think of a weekly average for the year. Your commute to work counts as leisure time)', and inquiring the hr/w of both light and hard PA. | FM | structured questionnaire inquiring past FM diagnosis by physician | 14172 | 0.03 | aRR+SE | age, BMI, education, anxiety and depression, smoking, insomnia symptoms | insomnia symptoms: yes, no* PA: high*, moderate, low FM: yes, no | no insomnia symptoms: low PA (vs high) -> FM | all possible combinations | 0.95+0.1581 | no insomnia symptoms: moderate PA (vs high) -> FM | 1.05+0.1253 | HUNT2-HUNT3. Only included in analysis with pain location subgroups | 4 | 2 | 1 |  |  |  |  |  |  |  |  |
| Skogberg 2022 ^116^ | CC | Sweden | adults 37.5 ± 13.4 | general public | Godin Leisure-Time Exercise Questionnaire | CP | clinical diagnosis by senior specialists in rehabilitation medicine | 176 | N/A | mean(n,SD) - mean(n,SD) | age, sex | PA: continuous pain: control, patients | PA: patients vs control | N/A | 33.17(78,22.28) - 45.97(98,26.02) | N/A | N/A | None | 3 | 2 | 2 |  |  |  |  |  |  |  |  |
| Smith 2019 ^117^ | CS | UK | middle-aged - elderly 65.8 ± 10.8 | general public | validated, structured, interview inquiring the frequency of mild, moderate, and vigorous PA respectively. | MSP | structured interview inquiring whether participants were often troubled by bone/joint/muscle pain | 9299 | 0.583 | Raw counts | N/A | PA intensity: mild, moderate, vigorous PA frequency: >1/w, 1/w, 1-3/mo, hardly ever or never often troubled with pain: yes, no | >=1/w moderate PA (vs <1/w) -> often troubled with pain | All | 2136, 4802, 1377, 984 | 1/w moderate PA (vs <1/w) -> often troubled with pain >1/w moderate PA (vs <1/w) -> often troubled with pain | 506, 856, 1377, 984 1630, 3946, 1377, 984 | ELSA wave 2 |  |  |  | T | T | T | N/A | F | F | F | F |
| Sollerhed 2013 ^118^ | CS | Sweden | children 9.93 ± 1.35 | students | structured questionnaire: "How often do you exercise in your leisure time (even brisk walking is counted as exercise)?" Answer can be "often", "sometimes", "seldom" or "never", where only "often" is considered a high perceived level of PA | recurrent pain (headache, abdominal pain, or back pain) | structured questionnaire | 206 | None | aOR+SE | gender, self-perceived physical fitness, self-perceived competence in PE, self-perceived body functions, subjective health, feel comfortable in school, living with both parents, physical fitness, BMI | recurrent pain: any*, headache, abdominal pain, back pain PA: high*, low | high PA (vs low) -> recurrent back pain | All | 0.8246+0.3691 | None | N/A | None |  |  |  | T | T | F | N/A | T | T | F | T |
| Solovev 2020 ^119^ | CH | Japanese | middle-aged - elderly 60.1 ± 8.8 | general public | structured questionnaire inquiring the frequency and duration spent at different levels of intensity for walking slowly, walking quickly, light to moderate exercise e.g., golf, croquet, and gardening), and strenuous exercise (e.g., tennis, jogging, aerobics, and swimming) for LTPA. Total MET-hr/d is calculated in a validated manner | CKP, CLBP | structured questionnaire inquiring any current chronic pain (pain persisting for >=6mo), and, site and severity if any. | 7565 | 0.152 | aOR+SE | sex, age, marital status, education, occupation, BMI, smoking, drinking, non-leisure-time PA (MET-hr/d) | gender: both*, M, F LTPA tertiles (MET-hr/d): 0, low (<1.0), medium (1-3.0), high (>=3.1) CP: any CLBP, serious CLBP, any CKP, serious CKP | both genders: medium LTPA -> any CLBP | All | 0.7491+0.1048 | both genders: low LTPA -> any CLBP both genders: high LTPA -> any CLBP M: medium LTPA -> any CLBP F: medium LTPA -> any CLBP | 0.9369+0.1001 1.0329+0.1055 0.8512+0.1535 0.6954+0.1483 | Murakami Cohort Study | 3 | 2 | 3 |  |  |  |  |  |  |  |  |
| Soysal 2013 ^120^ | CC | Turkey | adults - middle-aged 53.14 ± 5.57 | general public | IPAQ | CLBNP (CLBP or CNP) | record linkage | 96 | None | mean(n,SD) - mean(n,SD) | None | participants: CLBNP outpatients, CLBNP preoperative patients, healthy controls | CLBP outpatients vs controls: PA level (MET) | All | 5483.14(15,2906.85) - 14481.44(11,1834.88) | CNP outpatients vs controls: PA level (MET) | 8483.49(17,2089.76) - 14481.44(21,1834.88) | None | 2 | 0 | 2 |  |  |  |  |  |  |  |  |
| Stommen 2012 ^121^ | CC | Netherlands | adolescents - young adults 16.85 ± 2.50 | general public | SQUASH | MSP | structured questionnaire inquiring pain duration and intensity (with 10-cm VAS) | 84 | None | mean(n,SD) - mean(n,SD) | N/A | PA measurement: SQUASH total (MET-min/w), school-related activities (MET-min/w), leisure-time activities (MET-min/w), sports activities (MET-min/w), light activities (min/w), moderate activities (min/w), heavy activities (min/w) MSP: yes, no | MSP (yes vs no): leisure-time activities (MET-min/w) | All | 1194(42,1232) - 2001(42,2078) | MSP (yes vs no): SQUASH total score (MET-min/w) MSP (yes vs no): sports activities (MET-min/w) MSP (yes vs no): light activities (MET-min/w) MSP (yes vs no): moderate activities (min/w) MSP (yes vs no): high activities (min/w) | 5416(42,4987) - 6600(42,3219) 450(42,760) - 883(42,1416) 1175(42,1366) - 1745(42,1091) 375(42,380) - 256(42,487) 25(42,58) - 231(42,472) | None | 4 | 2 | 3 |  |  |  |  |  |  |  |  |
| Stričević 2015 ^122^ | CS | Slovenia | adults 37.8 ± 8.8 | nursing personnel | structured questionnaire inquiring any regular exercises, creation and sports in the youth and at the current state | non-specific LBP | structured questionnaire inquiring the number of LBP episodes experienced during the work career. >3 episodes is considered frequent | 659 | 0.827 | aOR+SE | exercise to prevent LBP, duration of employment >=19y, frequent manual lifting, work with computer >=2hr/d | LBP: frequent*, rare recreation and sports at present: yes, no* | recreation and sports at present: frequent LBP | N/A | 0.4000+0.2785 | N/A | N/A | None |  |  |  | F | F | F | N/A | T | T | F | T |
| Surís 2022 ^123^ | CS | Spain | adolescents - middle-aged 15-69 | general public | IPAQ | chronic back pain | structured questionnaire: "Could you tell us if you suffer or have suffered from any of the chronic disorders that I will now read to you? (We understand by chronic disorders those that have lasted at least 6 months or are permanent disorders): chronic lumbar or dorsal pain" | 16229 | 0.276 M: 0.241 F: 0.306 | aOR+SE | age, health status, tobacco consumption, alcohol consumption, BMI, any mental health problems, study level, social class, work satisfaction, social support, any financial difficulties | gender: M, F PA: low*, moderate, high chronic back pain: yes*, no | M, F: moderate PA -> chronic back pain | All | M: 0.7532+0.1076 F: 0.7409+0.0992 | M, F: high PA -> chronic back pain | M: 0.8427+0.1170 F: 0.7899+0.1403 | None |  |  |  | T | T | T | N/A | T | T | F | T |
| Swain 2016 ^124^ | CS | Europe and North America | adolescents | general public | frequency of MVPA: "Over the past 7 days (week), on how many days were you physically active for a total of at least 60 min per day?", where 0-6d/w is considered under-active for 5-17y | headache, stomach-ache, and backache | structured questionnaire inquiring frequency of pain | 242103 | 0.203 | Raw counts aOR+SE | survey year, concurrent headache, concurrent stomach-ache | M, F age: 11y, 13y, 15y active: yes, no* pain: none, backache | M, F: active -> backache | All | M: 883, 4589, 5128, 21247 F: 2325, 10265, 7955, 30046 | M 11y: active -> backache M 13y: active -> backache M 15y: active -> backache F 11y: active -> backache F 13y: active -> backache F 15y: active -> backache | 0.8485+0.0630 0.8371+0.0699 0.9567+0.0849 0.8608+0.0503 0.9015+0.0452 0.9366+0.0435 | health behavior in school-aged children (HBSC) study 2001/02, 2005/06 |  |  |  | T | T | T | N/A | T | T | F | T |
| Teichtahl 2015 ^125^ | CS | Australia | adults 45.91 ± 1.40 | general public | structured questionnaire inquiring number days in the previous 14d with >=20min/d strenuous activity leading to sweating or shortness of breath, e.g., swimming, tennis, netball, athletics and running | CLBP | CPGS | 72 | 0.467 | Raw counts | N/A | PA: inactive, moderate active, active CLBP: pain free, high pain/disability | moderate active -> high pain/disability CLBP | All | 8, 30, 7, 8 | active -> high pain/disability CLBP | 0, 19, 7, 8 | None |  |  |  | T | F | T | N/A | F | F | T | T |
| Thomas 1999 ^126^ | CH | USA | young adults - elderly | general public | level of PA assessed by structured questionnaire | persistent disabling LBP: the presence of both low back pain and disability (Hanover score < 75%) at each follow up interview (1 week and 3 and 12 months). | validated, structured, interview inquiring the presence of LBP on that day, the severity of any pain with 0-10 VAS. Disability was measured with the Hanover back pain activity schedule | 180 | 0.747 | OR+SE | N/A | PA compared with peers: more/same*, less persistent LBP: yes, no | more/same PA compared with peers (vs less) -> persistent LBP | N/A | 0.3571+0.3536 | N/A | N/A | None | 4 | 0 | 2 |  |  |  |  |  |  |  |  |
| Umeda 2015 ^127^ | CC | USA | F adults 45.1 ± 11.6 | general public | BPAQ | FM | record linkage for established FM diagnosis by physician, and FIQ | 28 | N/A | mean(n,SD) - mean(n,SD) | age, gender | PA (continuous): leisure, sport pain: FM, control | leisure PA: FM vs control | sport PA: FM vs control | 2.48(14,0.76) - 3.04(14,0.49) | sport PA: FM vs control | 1.96(14,0.89) - 2.98(14,0.68) | None | 2 | 2 | 2 |  |  |  |  |  |  |  |  |
| Umeda 2019 ^128^ | CS | USA | adults | general public | self-reporting of PA behaviours over the past 30d, which was used to calculate the frequency of duration of each activity. | CP | structured interview asking if the participant had a problem with pain that lasted >24hr during the past 1mo. Positive responses were followed by inquiries regarding duration of symptom and body regions affected with a pictorial manikin as aid | 14449 | None | mean(n,SD) - mean(n,SD) | age group, race/ethnicity, education level, marital status, weight status, chronic health conditions | gender: both*, M, F LTPA: total, moderate, vigorous, total aerobic???moderate aerobic, vigorous aerobic CP: no, localised, widespread | Both genders: total LTPA (any CP vs no CP) | All | 2.73(2112,10.0103) - 3.23(12387,23.3723) | M, F: total LTPA (any CP vs no CP) | M: 3.47(904,9.2972) - 3.85(6184,19.6596) F: 2.09(1208,6.5783) - 2.61(6203,15.7518) | NHANES 1999-2004 |  |  |  | T | T | F | N/A | T | T | F | T |
| van Oostrom 2011 ^129^ | CH | Netherlands | adults 45.9 ± 10.0 | general public | validated questionnaire, from which total time spent on MVPA was calculated | long-lasting LBP | structured questionnaire: 1. "Have you had trouble, discomfort or pain in the lower part of the back during the last 12 months?" 2. At first follow-up, "In the past 12 months, how long in total did you have LBP?", where >12w is considered long-lasting 3. At second follow-up, "How long have you had LBP for at the moment?", >=1mo is considered long-lasting  4. At third follow-up, "Has your current LBP lasted for more than 3 months?", and positive responses indicating long-lasting LBP | 3830 | None | aOR+SE | gender, age (per 10y), educational level, work status, BMI categories, smoking | PA: active, inactive* long-standing LBP: yes*, no | physically active -> long-standing LBP | N/A | 0.8570+0.1180 | N/A | N/A | Doetinchem Cohort Study | 4 | 2 | 2 |  |  |  |  |  |  |  |  |
| van Oostrom 2012 ^130^ | CH | Netherlands | adults 47.4 ± 10.1 | general public | validated EPIC Physical Activity Questionnaire yielding hr/w of MVPA and heavy work | bodily pain | Dutch version of RAND36, similar to SF-36 | 2631 | None | mean(n,SD) - mean(n,SD) | gender, age, education, living alone, work status, smoking, baseline and follow-up chronic disease status, baseline HRQoL | PA: becoming active, persistently active, persistent inactive, becoming inactive, varying activity levels bodily pain: continuous | persistently inactive vs persistently active -> bodily pain | All | 77.1(727,17.88) - 79.2(1286,18.30) | None | N/A | Doetinchem Cohort Study | 3 | 2 | 3 |  |  |  |  |  |  |  |  |
| van Weering 2011 ^131^ | CC | Netherlands | adults 43.23 ± 13.45 | general public | BPAQ | CLBP | structured interview inquiring duration of any CLBP | 52 | None | mean(n,SD) - mean(n,SD) | gender, age, disability level, duration of complaints | Patients vs controls: BPAQ total | Patients vs controls: BPAQ total | N/A | 8.6(27,1.2) - 8.9(20,0.9) | N/A | N/A | None | 3 | 0 | 2 |  |  |  |  |  |  |  |  |
| Vierola 2016 ^132^ | CS | Finland | children 7.6 ± 0.4 | students | PANIC Physical Activity Questionnaire inquiring the frequency, duration and type of PA | pain | structured questionnaire for parents: 1. "Did your child have pain within the past three months (yes or no)?" 2. "How often your child had pain within the past three months (never, seldom, once a month, several times a month, more than once a week, daily, or continuously)?" | 439 | None | aOR+SE | age, sedentary behavior , cardiorespiratory fitness, body fat percentage | PA tertiles (min/d): low*, medium, high pain: any, frequent, multiple | medium PA -> frequent pain | All | 1.6510+0.2713 | high PA -> frequent pain | 1.4892+0.2921 | PANIC study baseline |  |  |  | T | F | T | N/A | T | T | T | T |
| Wadley 2020 ^133^ | CS | South Africa | M adults 36.67 ± 8.15 | long-distance truck drivers | GSLTPAQ inquiring the weekly frequency of 15min episodes of strenuous (s), moderate (m) and light (l) exercise. LTPA score = 9 * s + 5 * m + 3 * l | CP | structured questionnaire asking participants to indicate any locations of pain from a list of body parts, where they had had pain or discomfort for more than 3mo | 614 | None | aOR+SE | age, BMI, smoking, depressive symptoms , post-traumatic stress disorder, working >=2 nights/w | LTPA score: continuous CP: yes*, no | LTPA score -> CP | N/A | 0.9689+0.007899 | N/A | N/A | None |  |  |  | T | F | T | N/A | T | T | F | T |
| Whibley 2020 ^134^ | CH | USA | middle-aged - elderly 66.9 ± 29.74 | general public | structured interview: 1. "How often do you take part in sports or activities that are moderately energetic, such as gardening, cleaning the car, walking at a moderate pace, dancing, floor or stretching exercises?" 2. "How often do you take part in sports or activities that are vigorous, such as running or jogging, swimming, cycling, aerobics or gym workout, tennis, or digging with a spade or shovel?" A 0-18 PA index score is derived from the results, with <6 meaning physical inactivity, >9 meaning meeting WHO's PA recommendations | incident troublesome pain | validated structured interview: "Are you often troubled with pain?" | 9828 | 0.21 | aOR+SE | sleep disturbance frequency, physical activity - sleep disturbance interaction term, age, gender, BMI, race/ethnicity, years of school, history of depression, history of major disease | PA: MVPA >1/w, MVPA <=1/w, continuous (PA index score) troublesome pain: yes*, no | PA index score -> troublesome pain | PA index score -> troublesome pain | 0.965+0.013 | MVPA >1/w -> troublesome pain incidence | 680, 3276, 544, 2048 | HRS 2014-2016 | 4 | 2 | 2 |  |  |  |  |  |  |  |  |
| Yang 2018 ^135^ | CS | USA | adults | general public | structured interview inquiring intensity, duration and frequency of aerobic PA | CLBP | structured interview inquiring whether the participant experienced low back pain in the past three months prior to the interview | 122337 | None | aOR+SE | age, gender, race and ethnicity , education, yearly earning | gender: both, M, F LTPA: inactive*, insufficiently active, regularly active LBP: yes*, no | both genders: regularly active -> LBP | All | 0.8142+0.0219 | both genders: insufficiently active -> LBP M: regularly active -> LBP F: regularly active -> LBP | 0.9439+0.0243 0.8338+0.0275 0.8138+0.0282 | NHIS 2009-2012 |  |  |  | T | T | F | N/A | T | T | F | T |
| Yiengprugsawan 2017 ^136^ | CH | Thailand | adults | general public | combined number of MVPA sessions/w inquired structured questionnaire, only 4y follow-up data are reported | LBP | structured questionnaire asking standardised questions about LBP at both baseline and 4y follow-up | 42785 | 0.309 | Raw counts | N/A | PA/w: <3, 3-6, >=7 Pain: never, reverting, incident, chronic | 3-6 PA/w (vs <3/w) -> chronic pain (vs never) | All | 4572, 6113, 6637, 7668 | >=7 PA/w (vs <3/w) -> chronic pain (vs never) | 1618, 1958, 6337, 7668 | None | 3 | 0 | 2 |  |  |  |  |  |  |  |  |
| Zanuto 2020 ^137^ | CS | Brazil | middle-aged - elderly 62 ± 8.8 | general public | BPAQ. Results were dichotomised at the 75th percentile | CLBP | structured questionnaire inquiring: 1. the presence of musculoskeletal disorders over the previous 12 months 2. impairment of daily activities over the previous 12 months, due to these disorders 3. consulting a healthcare professional because of these disorders 4. feeling the presence of these disorders in the week immediately prior to the interview Positive response to all questions above is considered CLBP positive | 327 M: 98 F: 229 | 0.245 | aOR+SE | sex, age, ethnicity economic situation, BMI, cycling, work | physical exercise: yes, no* CLBP: yes*, no | physical exercise -> CLBP | N/A | 0.3560+0.4128 | N/A | N/A | None |  |  |  | T | F | T | N/A | T | T | F | T |

**Remarks**

^@^ Citation matches that in the manuscript main text.

^@@^ CC = Case-control, CH = Cohort, CS = Cross-sectional

^*^ Reference category

^**^ age is expressed as mean ± SD when mean and SD are available. The SD is omitted in this table if the data is not available in the manuscript.

^***^ LTPA = leisure-time physical activity, PA = physical activity, TPA = total physical activity, IPAQ-SF = International Physical Activity Questionnaire – Short Form, IPAQ-LF = International Physical Activity Questionnaire – Long Form, GPAQ = Global Physical Activity Questionnaire, BPAQ = Baecke Physical Activity Questionnarie, SQUASH = Short Questionnaire to Assess Health enhancing physical activity, MET = metabolic equivalent of task

^^^ CMSP = chronic musculoskeletal pain, MSP = musculoskeletal pain, CP = chronic pain, CLBP = chronic low back pain, CNSP = chronic neck/shoulder pain, CNP = chronic neck pain, CKP = chronic knee pain, CWP chronic widespread pain, NP = neck pain, NSP = neck/shoulder pain, BP = back pain, LBP = low back pain, FM = fibromyalgia

^^^^ NMQ = standardized Nordic Musculoskeletal Questionnaire, CPGS = Chronic Pain Grade Scale, FIQ = Fibromyalgia Impact Questionnaire, SF-36 Short Form 36, SF-12 Short Form 12, SF-8 Short Form 8, VAS = Visual Analog Scale, NRS = Numerical Rating Scale, EQ-5D-3L = EuroQoL quality of life questionnaire

^^^^^ ACR = assumed control risk (used for converting risk ratios to odds ratios)

^#^ Raw counts = “high LTPA with CMSP, high LTPA without CMSP, low LTPA with CMSP, low LTPA without CMSP”, OR = odds ratio, aOR = adjusted odds ratio, RR = risk ratio, aRR = adjusted risk ratio, PR = prevalence ratio, aPR = adjusted prevalence ratio, SE = standard error, SD = standard deviation

^##^ M = male, F = female, d = day, h = hour, w = week, mo = month, min = minutes

^###^ JBI = JBI checklist for cross-sectional studies, NOS = Newcastle-Ottawa Scale (Selection, Comparability, Exposure/Outcome)

**References**

1. Abumunaser LA, Alfaraj KA, Kamal LK, Alzahrani RA, Alzahrani MM, AlAhmed AB. Lower Back Pain Caused by the Impact of COVID-19 Quarantine on Physical Activity and Daily Sitting Among Adult Saudi Arabian Populations in Jeddah: A Cross-Sectional Study. *Orthop Res Rev*. 2022;14:477-485. doi:10.2147/ORR.S386995

2. Adnan R, Van Oosterwijck J, Danneels L, et al. Differences in psychological factors, disability and fatigue according to the grade of chronification in non-specific low back pain patients: A cross-sectional study. *J Back Musculoskelet Rehabil*. 2020;33(6):919-930. doi:10.3233/BMR-191548

3. Alzahrani H, Shirley D, Cheng SWM, Mackey M, Stamatakis E. Physical activity and chronic back conditions: A population-based pooled study of 60,134 adults. *J Sport Health Sci*. Jul 2019;8(4):386-393. doi:10.1016/j.jshs.2019.01.003

4. A BA, Simic M, Pappas E, et al. Is occupational or leisure physical activity associated with low back pain? Insights from a cross-sectional study of 1059 participants. *Braz J Phys Ther*. May-Jun 2019;23(3):257-265. doi:10.1016/j.bjpt.2018.06.004

5. Andias R, Silva AG. The Onset of Chronic Musculoskeletal Pain in High School Adolescents: Associated Factors and the Role of Symptoms of Central Sensitization. *Phys Ther*. Apr 1 2022;102(4)doi:10.1093/ptj/pzab286

6. Andorsen OF, Ahmed LA, Emaus N, Klouman E. A prospective cohort study on risk factors of musculoskeletal complaints (pain and/or stiffness) in a general population. The Tromso study. *PLoS One*. 2017;12(7):e0181417. doi:10.1371/journal.pone.0181417

7. Astfalck RG, O'Sullivan PB, Straker LM, Smith AJ. A detailed characterisation of pain, disability, physical and psychological features of a small group of adolescents with non-specific chronic low back pain. *Man Ther*. Jun 2010;15(3):240-7. doi:10.1016/j.math.2009.12.007

8. Auvinen J, Tammelin T, Taimela S, Zitting P, Karppinen J. Associations of physical activity and inactivity with low back pain in adolescents. *Scand J Med Sci Sports*. Apr 2008;18(2):188-94. doi:10.1111/j.1600-0838.2007.00672.x

9. Baker TA, Vasquez E, Minahan JA. Variability of Pain Outcomes and Physical Activity Among a Diverse Sample of Older Men: Is It More Than Just Race? *Gerontol Geriatr Med*. Jan-Dec 2019;5:2333721419878587. doi:10.1177/2333721419878587

10. Barbosa REC, Fonseca GC, Souza ESNS, Silva RRV, Assuncao AA, Haikal DS. Back pain occurred due to changes in routinary activities among Brazilian schoolteachers during the COVID-19 pandemic. *Int Arch Occup Environ Health*. Mar 2022;95(2):527-538. doi:10.1007/s00420-021-01793-w

11. Barro D, Olinto MT, Macagnan JB, et al. Job characteristics and musculoskeletal pain among shift workers of a poultry processing plant in Southern Brazil. *J Occup Health*. 2015;57(5):448-56. doi:10.1539/joh.14-0201-OA

12. Basler HD, Luckmann J, Wolf U, Quint S. Fear-avoidance beliefs, physical activity, and disability in elderly individuals with chronic low back pain and healthy controls. *Clin J Pain*. Sep 2008;24(7):604-10. doi:10.1097/AJP.0b013e31816b54f6

13. Batista GA, Locks F, Beltran Picon SP, et al. Association between the time of physical laboral activities and leisure over shoulder pain in fruitculture workers: A cross-sectional study. *Work*. 2023;74(2):565-574. doi:10.3233/WOR-210851

14. Bjorck-van Dijken C, Fjellman-Wiklund A, Hildingsson C. Low back pain, lifestyle factors and physical activity: a population based-study. *J Rehabil Med*. Nov 2008;40(10):864-9. doi:10.2340/16501977-0273

15. Bjorkegren K, Wallander MA, Johansson S, Svardsudd K. General symptom reporting in female fibromyalgia patients and referents: a population-based case-referent study. *BMC Public Health*. Oct 31 2009;9:402. doi:10.1186/1471-2458-9-402

16. Bjornsdottir SV, Jonsson SH, Valdimarsdottir UA. Functional limitations and physical symptoms of individuals with chronic pain. *Scand J Rheumatol*. 2013;42(1):59-70. doi:10.3109/03009742.2012.697916

17. Bollinger MJ, Hudson TJ, Hu B, Han X, Long CR, McElfish P. The relationship between sociodemographic, behavioral, and clinical variables and pain in the Native Hawaiian and Pacific Islander population. *Asian American Journal of Psychology*. 2020;11:49-58. doi:10.1037/aap0000173

18. Brady SRE, Hussain SM, Brown WJ, et al. Relationships Between Weight, Physical Activity, and Back Pain in Young Adult Women. *Medicine (Baltimore)*. May 2016;95(19):e3368. doi:10.1097/MD.0000000000003368

19. Brady SRE, Monira Hussain S, Brown WJ, et al. Predictors of Back Pain in Middle-Aged Women: Data From the Australian Longitudinal Study of Women's Health. *Arthritis Care Res (Hoboken)*. May 2017;69(5):709-716. doi:10.1002/acr.22982

20. Brindova D, Veselska ZD, Klein D, et al. Is the association between screen-based behaviour and health complaints among adolescents moderated by physical activity? *Int J Public Health*. Feb 2015;60(2):139-45. doi:10.1007/s00038-014-0627-x

21. Brown WJ, Mishra G, Lee C, Bauman A. Leisure time physical activity in Australian women: relationship with well being and symptoms. *Res Q Exerc Sport*. Sep 2000;71(3):206-16. doi:10.1080/02701367.2000.10608901

22. Burton AK, Tillotson KM. Does leisure sports activity influence lumbar mobility or the risk of low back trouble? *J Spinal Disord*. Sep 1991;4(3):329-36. doi:10.1097/00002517-199109000-00010

23. Carpintero-Rubio C, Torres-Chica B, Guadron-Romero MA, Visiers-Jimenez L, Pena-Otero D. Perception of musculoskeletal pain in the state of confinement: associated factors. *Rev Lat Am Enfermagem*. 2021;29:e3454. doi:10.1590/1518-8345.4894.3454

24. Chaiklieng S, Suggaravetsiri P, Andajani S. Risk Factors Associated with Work-Related Low Back Pain Among Home-Based Garment Workers. Springer International Publishing; 2020:753-759.

25. Chun MY, Cho BJ, Yoo SH, Oh B, Kang JS, Yeon C. Association between sleep duration and musculoskeletal pain: The Korea National Health and Nutrition Examination Survey 2010-2015. *Medicine (Baltimore)*. Dec 2018;97(50):e13656. doi:10.1097/MD.0000000000013656

26. Citko A, Gorski S, Marcinowicz L, Gorska A. Sedentary Lifestyle and Nonspecific Low Back Pain in Medical Personnel in North-East Poland. *Biomed Res Int*. 2018;2018:1965807. doi:10.1155/2018/1965807

27. Constantino Coledam DH, Junior RP, Ribeiro EAG, de Oliveira AR. Factors associated with musculoskeletal disorders and disability in elementary teachers: A cross-sectional study. *J Bodyw Mov Ther*. Jul 2019;23(3):658-665. doi:10.1016/j.jbmt.2018.05.009

28. Correa-Rodriguez M, El Mansouri-Yachou J, Tapia-Haro RM, Molina F, Rueda-Medina B, Aguilar-Ferrandiz ME. Associations Between Bone Mass in Women With Fibromyalgia and Widespread Pressure Pain Hypersensitivity, Tenderness, Perceived Pain Level, and Disability. *Biol Res Nurs*. May 2019;21(3):272-278. doi:10.1177/1099800419838625

29. D'Onise R, Shanahan EM, Gill T, Hill CL. Does leisure time physical activity protect against shoulder pain at work? *Occup Med (Lond)*. Aug 2010;60(5):383-8. doi:10.1093/occmed/kqq050

30. Decharat S, Phethuayluk P, Maneelok S. Prevalence of Musculoskeletal Symptoms among Dental Health Workers, Southern Thailand. *Adv Prev Med*. 2016;2016:5494821. doi:10.1155/2016/5494821

31. Drozda K, Lewandowski J, Gorski P. Back pain in lower and upper secondary school pupils living in urban areas of Poland. The case of Poznan. *Ortop Traumatol Rehabil*. Sep-Oct 2011;13(5):489-503. doi:10.5604/15093492.967218

32. Eggermont LH, Milberg WP, Lipsitz LA, Scherder EJ, Leveille SG. Physical activity and executive function in aging: the MOBILIZE Boston Study. *J Am Geriatr Soc*. Oct 2009;57(10):1750-6. doi:10.1111/j.1532-5415.2009.02441.x

33. El-Metwally A, Salminen JJ, Auvinen A, Macfarlane G, Mikkelsson M. Risk factors for development of non-specific musculoskeletal pain in preteens and early adolescents: a prospective 1-year follow-up study. *BMC Musculoskelet Disord*. May 23 2007;8:46. doi:10.1186/1471-2474-8-46

34. Fanavoll R, Nilsen TI, Holtermann A, Mork PJ. Psychosocial work stress, leisure time physical exercise and the risk of chronic pain in the neck/shoulders: Longitudinal data from the Norwegian HUNT Study. *Int J Occup Med Environ Health*. 2016;29(4):585-95. doi:10.13075/ijomeh.1896.00606

35. Feldman DE, Shrier I, Rossignol M, Abenhaim L. Risk factors for the development of neck and upper limb pain in adolescents. *Spine (Phila Pa 1976)*. 2002;27(5):523-8. doi:10.1097/00007632-200203010-00013

36. Feller H, Baker N. Prevalence of leisure participation in older adults with and without pain: A secondary data analysis of the 2014 Health and Retirement Study. *Chronic Illn*. 2020:1742395320949615. doi:10.1177/1742395320949615

37. Fernandez-de-las-Penas C, Hernandez-Barrera V, Alonso-Blanco C, et al. Prevalence of neck and low back pain in community-dwelling adults in Spain: a population-based national study. *Spine (Phila Pa 1976)*. Feb 1 2011;36(3):E213-9. doi:10.1097/BRS.0b013e3181d952c2

38. Fernandez-de-Las-Penas C, Alonso-Blanco C, Hernandez-Barrera V, Palacios-Cena D, Jimenez-Garcia R, Carrasco-Garrido P. Has the prevalence of neck pain and low back pain changed over the last 5 years? A population-based national study in Spain. *Spine J*. Sep 2013;13(9):1069-76. doi:10.1016/j.spinee.2013.02.064

39. Fjeld MK, Arnes AP, Engdahl B, et al. Consistent pattern between physical activity measures and chronic pain levels: the Tromso Study 2015 to 2016. *Pain*. Apr 1 2023;164(4):838-847. doi:10.1097/j.pain.0000000000002773

40. Garcia-Heras F, Gutierrez-Arroyo J, Leon-Guereno P, Carballo-Leyenda B, Rodriguez-Marroyo JA. Chronic Pain in Spanish Wildland Firefighters. *J Clin Med*. Feb 14 2022;11(4)doi:10.3390/jcm11040989

41. Ghim S, Ku B. The prevalence of health problems and their association with physical activity in caregivers of children with disabilities: 2018 National Health Interview Survey. *Child Care Health Dev*. Mar 2022;48(2):347-357. doi:10.1111/cch.12934

42. Grasdalsmoen M, Engdahl B, Fjeld MK, et al. Physical exercise and chronic pain in university students. *PLoS One*. 2020;15(6):e0235419. doi:10.1371/journal.pone.0235419

43. Graup S, de Araujo Bergmann ML, Bergmann GG. Prevalence of nonspecific lumbar pain and associated factors among adolescents in Uruguaiana, state of Rio Grande do Sul. *Rev Bras Ortop*. Nov-Dec 2014;49(6):661-7. doi:10.1016/j.rboe.2014.10.003

44. Halonen JI, Shiri R, Magnusson Hanson LL, Lallukka T. Risk and Prognostic Factors of Low Back Pain: Repeated Population-based Cohort Study in Sweden. *Spine (Phila Pa 1976)*. Sep 1 2019;44(17):1248-1255. doi:10.1097/BRS.0000000000003052

45. Harithasan D, Singh DKA, Abd Razak NAB, Baharom NB. Personal, Academic Stressors and Environmental Factors Contributing to Musculoskeletal Pain among Undergraduates Due to Online Learning: A Mixed Method Study with Data Integration. *Int J Environ Res Public Health*. Nov 4 2022;19(21)doi:10.3390/ijerph192114513

46. Hartvigsen J, Christensen K. Active lifestyle protects against incident low back pain in seniors: a population-based 2-year prospective study of 1387 Danish twins aged 70-100 years. *Spine (Phila Pa 1976)*. Jan 1 2007;32(1):76-81. doi:10.1097/01.brs.0000250292.18121.ce

47. Heuch I, Heuch I, Hagen K, Zwart JA. Is there a U-shaped relationship between physical activity in leisure time and risk of chronic low back pain? A follow-up in the HUNT Study. *BMC Public Health*. Apr 11 2016;16:306. doi:10.1186/s12889-016-2970-8

48. Hill J, Lewis M, Papageorgiou AC, Dziedzic K, Croft P. Predicting persistent neck pain: a 1-year follow-up of a population cohort. *Spine (Phila Pa 1976)*. Aug 1 2004;29(15):1648-54. doi:10.1097/01.brs.0000132307.06321.3c

49. Holmberg S, Thelin A, Stiernstrom E, Svardsudd K. The impact of physical work exposure on musculoskeletal symptoms among farmers and rural non-farmers. *Ann Agric Environ Med*. 2003;10(2):179-84.

50. Holth HS, Werpen HK, Zwart JA, Hagen K. Physical inactivity is associated with chronic musculoskeletal complaints 11 years later: results from the Nord-Trondelag Health Study. *BMC Musculoskelet Disord*. Dec 1 2008;9:159. doi:10.1186/1471-2474-9-159

51. Hulsegge G, van Oostrom SH, Picavet HS, et al. Musculoskeletal complaints among 11-year-old children and associated factors: the PIAMA birth cohort study. *Am J Epidemiol*. Oct 15 2011;174(8):877-84. doi:10.1093/aje/kwr205

52. Hussain SM, Urquhart DM, Wang Y, et al. Associations between television viewing and physical activity and low back pain in community-based adults: A cohort study. *Medicine (Baltimore)*. Jun 2016;95(25):e3963. doi:10.1097/MD.0000000000003963

53. Ilhan B, Bahat G, Erdogan T, Kilic C, Karan MA. Chronic pain: prevalent and independently associated with frailty and female gender in geriatric outpatients. *Eur Geriatr Med*. Dec 2019;10(6):931-937. doi:10.1007/s41999-019-00235-8

54. Jia N, Zhang M, Zhang H, et al. Prevalence and risk factors analysis for low back pain among occupational groups in key industries of China. *BMC Public Health*. Aug 5 2022;22(1):1493. doi:10.1186/s12889-022-13730-8

55. Junqueira DR, Ferreira ML, Refshauge K, et al. Heritability and lifestyle factors in chronic low back pain: results of the Australian twin low back pain study (The AUTBACK study). *Eur J Pain*. Nov 2014;18(10):1410-8. doi:10.1002/ejp.506

56. Kaartinen S, Aaltonen S, Korhonen T, et al. Is diversity of leisure-time sport activities associated with low back and neck-shoulder region pain? A Finnish twin cohort study. *Prev Med Rep*. Sep 2019;15:100933. doi:10.1016/j.pmedr.2019.100933

57. Kahere M, Ginindza T. The prevalence and risk factors of chronic low back pain among adults in KwaZulu-Natal, South Africa: an observational cross-sectional hospital-based study. *BMC Musculoskelet Disord*. 2021;22(1):955. doi:10.1186/s12891-021-04790-9

58. Kallings LV, Blom V, Ekblom B, et al. Workplace sitting is associated with self-reported general health and back/neck pain: a cross-sectional analysis in 44,978 employees. *BMC Public Health*. May 6 2021;21(1):875. doi:10.1186/s12889-021-10893-8

59. Kamada M, Kitayuguchi J, Lee IM, et al. Relationship between physical activity and chronic musculoskeletal pain among community-dwelling Japanese adults. *J Epidemiol*. 2014;24(6):474-83. doi:10.2188/jea.je20140025

60. Karjalainen U, Paananen M, Okuloff A, et al. Role of environmental factors and history of low back pain in sciatica symptoms among Finnish adolescents. *Spine (Phila Pa 1976)*. Jun 1 2013;38(13):1105-11. doi:10.1097/BRS.0b013e318287fb3a

61. Karunanayake AL, Pathmeswaran A, Kasturiratne A, Wijeyaratne LS. Risk factors for chronic low back pain in a sample of suburban Sri Lankan adult males. *Int J Rheum Dis*. Apr 2013;16(2):203-10. doi:10.1111/1756-185X.12060

62. Kedra A, Kolwicz-Ganko A, Kedra P, Bochenek A, Czaprowski D. Back pain in physically inactive students compared to physical education students with a high and average level of physical activity studying in Poland. *BMC Musculoskelet Disord*. Nov 28 2017;18(1):501. doi:10.1186/s12891-017-1858-9

63. Kemta Lekpa F, Enyama D, Noukeu Njinkui D, et al. Prevalence and factors associated with low back pain in schoolchildren in Cameroon, sub-Saharan Africa. *Int J Rheum Dis*. Sep 2021;24(9):1186-1191. doi:10.1111/1756-185X.14172

64. Kesiena O, Ajayi KV, Rene A, Benden M. Sociodemographic and work-related predictors of chronic lower back pain in the United States: the 2018 National Health Interview Survey data. *Public Health*. Sep 2021;198:30-34. doi:10.1016/j.puhe.2021.06.015

65. Kim Y, Umeda M. Chronic Pain, Physical Activity, and All-Cause Mortality in the US Adults: The NHANES 1999-2004 Follow-Up Study. *Am J Health Promot*. Nov 2019;33(8):1182-1186. doi:10.1177/0890117119854041

66. Kirsch Micheletti J, Blafoss R, Sundstrup E, Bay H, Pastre CM, Andersen LL. Association between lifestyle and musculoskeletal pain: cross-sectional study among 10,000 adults from the general working population. *BMC Musculoskelet Disord*. Dec 17 2019;20(1):609. doi:10.1186/s12891-019-3002-5

67. Koneru S, Tanikonda R. Role of yoga and physical activity in work-related musculoskeletal disorders among dentists. *J Int Soc Prev Community Dent*. May-Jun 2015;5(3):199-204. doi:10.4103/2231-0762.159957

68. Kopec JA, Cibere J, Li LC, et al. Relationship between physical activity and hip pain in persons with and without cam or pincer morphology: a population-based case-control study. *Osteoarthritis Cartilage*. Jul 2017;25(7):1055-1061. doi:10.1016/j.joca.2017.02.795

69. Kossi O, Yamadjako D, Timmermans A, Michiels S, Adoukonou T, Janssens L. Prevalence and biopsychosocial factors associated with chronic low back pain in urban and rural communities in Western Africa: a population-based door-to-door survey in Benin. *Eur Spine J*. Nov 2022;31(11):2897-2906. doi:10.1007/s00586-022-07345-1

70. Kovacs FM, Arana E, Royuela A, et al. Vertebral endplate changes are not associated with chronic low back pain among Southern European subjects: a case control study. *AJNR Am J Neuroradiol*. Sep 2012;33(8):1519-24. doi:10.3174/ajnr.A3087

71. Kumar G, Chhabra A, Dewan V, Yadav TP. Idiopathic musculoskeletal pain in Indian children-Prevalence and impact on daily routine. *Rev Bras Reumatol Engl Ed*. Jan-Feb 2017;57(1):8-14. doi:10.1016/j.rbre.2015.07.015

72. Landis CA, Lentz MJ, Tsuji J, Buchwald D, Shaver JL. Pain, psychological variables, sleep quality, and natural killer cell activity in midlife women with and without fibromyalgia. *Brain Behav Immun*. Jul 2004;18(4):304-13. doi:10.1016/j.bbi.2003.11.001

73. Landmark T, Romundstad P, Borchgrevink PC, Kaasa S, Dale O. Associations between recreational exercise and chronic pain in the general population: evidence from the HUNT 3 study. *Pain*. Oct 2011;152(10):2241-2247. doi:10.1016/j.pain.2011.04.029

74. Lee CY, Kratter R, Duvoisin N, Taskin A, Schilling J. Cross-sectional view of factors associated with back pain. *Int Arch Occup Environ Health*. May 2005;78(4):319-24. doi:10.1007/s00420-004-0589-z

75. Lewandowski J, Lukaszewska K. Characteristics of back pain in Polish youth depending on place of residence. *Ann Agric Environ Med*. 2014;21(3):644-8. doi:10.5604/12321966.1120618

76. Li FD, Kong QJ, Wang YX, Sun KQ, Zheng B, Shi JG. Predicting the risk of non-specific low back pain in the young population: development and assessment of a new predictive nomogram. *Eur Rev Med Pharmacol Sci*. Dec 2022;26(23):8795-8807. doi:10.26355/eurrev_202212_30551

77. Lilje SC, Skillgate E, Anderberg P, Berglund J. Negative psychosocial and heavy physical workloads associated with musculoskeletal pain interfering with normal life in older adults: cross-sectional analysis. *Scand J Public Health*. Jul 2015;43(5):453-9. doi:10.1177/1403494815580876

78. Lopez-Bueno R, Blafoss R, Calatayud J, et al. Association Between Physical Activity and Odds of Chronic Conditions Among Workers in Spain. *Prev Chronic Dis*. Oct 8 2020;17:E121. doi:10.5888/pcd17.200105

79. Macedo LB, Costa de Assis SJ, Pereira NKF, Cacho RO, de Souza CG. Consequences of social distancing during the COVID-19 pandemic on the increase in perceived pain of students and professors from higher education institutions: A cross-sectional study. *Work*. 2022;73(3):761-768. doi:10.3233/WOR-211053

80. Macfarlane TV, McBeth J, Jones GT, Nicholl B, Macfarlane GJ. Whether the weather influences pain? Results from the EpiFunD study in North West England. *Rheumatology (Oxford)*. Aug 2010;49(8):1513-20. doi:10.1093/rheumatology/keq099

81. Markkula RA, Kalso EA, Kaprio JA. Predictors of fibromyalgia: a population-based twin cohort study. *BMC Musculoskelet Disord*. Jan 15 2016;17:29. doi:10.1186/s12891-016-0873-6

82. McLoughlin MJ, Colbert LH, Stegner AJ, Cook DB. Are women with fibromyalgia less physically active than healthy women? *Med Sci Sports Exerc*. May 2011;43(5):905-12. doi:10.1249/MSS.0b013e3181fca1ea

83. McLoughlin MJ, Stegner AJ, Cook DB. The relationship between physical activity and brain responses to pain in fibromyalgia. *J Pain*. Jun 2011;12(6):640-51. doi:10.1016/j.jpain.2010.12.004

84. Meucci RD, Fassa AG, Paniz VM, Silva MC, Wegman DH. Increase of chronic low back pain prevalence in a medium-sized city of southern Brazil. *BMC Musculoskelet Disord*. May 1 2013;14:155. doi:10.1186/1471-2474-14-155

85. Mikkelsson M, Salminen JJ, Sourander A, Kautiainen H. Contributing factors to the persistence of musculoskeletal pain in preadolescents: a prospective 1-year follow-up study. *Pain*. Jul 1998;77(1):67-72. doi:10.1016/S0304-3959(98)00083-9

86. Miranda H, Viikari-Juntura E, Martikainen R, Takala EP, Riihimaki H. A prospective study of work related factors and physical exercise as predictors of shoulder pain. *Occup Environ Med*. Aug 2001;58(8):528-34. doi:10.1136/oem.58.8.528

87. Mork PJ, Vasseljen O, Nilsen TI. Association between physical exercise, body mass index, and risk of fibromyalgia: longitudinal data from the Norwegian Nord-Trondelag Health Study. *Arthritis Care Res (Hoboken)*. May 2010;62(5):611-7. doi:10.1002/acr.20118

88. Mork PJ, Vik KL, Moe B, Lier R, Bardal EM, Nilsen TI. Sleep problems, exercise and obesity and risk of chronic musculoskeletal pain: the Norwegian HUNT study. *Eur J Public Health*. Dec 2014;24(6):924-9. doi:10.1093/eurpub/ckt198

89. Myrtveit SM, Sivertsen B, Skogen JC, Frostholm L, Stormark KM, Hysing M. Adolescent neck and shoulder pain--the association with depression, physical activity, screen-based activities, and use of health care services. *J Adolesc Health*. Sep 2014;55(3):366-72. doi:10.1016/j.jadohealth.2014.02.016

90. Nah S, Park SS, Choi S, Jang HD, Moon JE, Han S. Associations of walking and resistance training with chronic low back pain in older adults: A cross-sectional analysis of Korean National Health and Nutrition Examination Survey data. *Medicine (Baltimore)*. Mar 18 2022;101(11)doi:10.1097/MD.0000000000029078

91. Natvig B, Bruusgaard D, Eriksen W. Physical leisure activity level and physical fitness among women with fibromyalgia. *Scand J Rheumatol*. 1998;27(5):337-41. doi:10.1080/03009749850154348

92. Nilsen TI, Holtermann A, Mork PJ. Physical exercise, body mass index, and risk of chronic pain in the low back and neck/shoulders: longitudinal data from the Nord-Trondelag Health Study. *Am J Epidemiol*. Aug 1 2011;174(3):267-73. doi:10.1093/aje/kwr087

93. Noormohammadpour P, Mansournia MA, Asadi-Lari M, Nourian R, Rostami M, Kordi R. A Subtle Threat to Urban Populations in Developing Countries: Low Back Pain and its Related Risk Factors. *Spine (Phila Pa 1976)*. Apr 2016;41(7):618-27. doi:10.1097/BRS.0000000000001269

94. Noormohammadpour P, Mansournia MA, Koohpayehzadeh J, et al. Prevalence of Chronic Neck Pain, Low Back Pain, and Knee Pain and Their Related Factors in Community-Dwelling Adults in Iran: A Population-based National Study. *Clin J Pain*. Feb 2017;33(2):181-187. doi:10.1097/AJP.0000000000000396

95. O'Sullivan P, Beales D, Jensen L, Murray K, Myers T. Characteristics of chronic non-specific musculoskeletal pain in children and adolescents attending a rheumatology outpatients clinic: a cross-sectional study. *Pediatr Rheumatol Online J*. Jan 19 2011;9(1):3. doi:10.1186/1546-0096-9-3

96. Palmlof L, Holm LW, Alfredsson L, Magnusson C, Vingard E, Skillgate E. The impact of work related physical activity and leisure physical activity on the risk and prognosis of neck pain - a population based cohort study on workers. *BMC Musculoskelet Disord*. May 20 2016;17:219. doi:10.1186/s12891-016-1080-1

97. Park SM, Kim HJ, Jeong H, et al. Longer sitting time and low physical activity are closely associated with chronic low back pain in population over 50 years of age: a cross-sectional study using the sixth Korea National Health and Nutrition Examination Survey. *Spine J*. Nov 2018;18(11):2051-2058. doi:10.1016/j.spinee.2018.04.003

98. Payne N, Gledhill N, Katzmarzyk PT, Jamnik V. Health-related fitness, physical activity, and history of back pain. *Can J Appl Physiol*. Aug 2000;25(4):236-49. doi:10.1139/h00-018

99. Peltzer K. Lifestyle factors, mental health, and incident and persistent intrusive pain among ageing adults in South Africa. *Scand J Pain*. Jan 27 2023;23(1):161-167. doi:10.1515/sjpain-2022-0013

100. Peng T, Perez A, Pettee Gabriel K. The Association Among Overweight, Obesity, and Low Back Pain in U.S. Adults: A Cross-Sectional Study of the 2015 National Health Interview Survey. *J Manipulative Physiol Ther*. May 2018;41(4):294-303. doi:10.1016/j.jmpt.2017.10.005

101. Picavet HSJ, Gehring U, van Haselen A, et al. A widening gap between boys and girls in musculoskeletal complaints, while growing up from age 11 to age 20 - the PIAMA birth Cohort study. *Eur J Pain*. Apr 2021;25(4):902-912. doi:10.1002/ejp.1719

102. Queiroz LB, Lourenco B, Silva LEV, Lourenco DMR, Silva CA. Musculoskeletal pain and musculoskeletal syndromes in adolescents are related to electronic devices. *J Pediatr (Rio J)*. Nov-Dec 2018;94(6):673-679. doi:10.1016/j.jped.2017.09.006

103. Rashiq S, Dick BD. Factors associated with chronic noncancer pain in the Canadian population. *Pain Res Manag*. Nov-Dec 2009;14(6):454-60. doi:10.1155/2009/919628

104. Ray BM, Kelleran KJ, Eubanks JE, Nan N, Ma C, Miles D. Relationship between Physical Activity and Pain in U.S. Adults. *Med Sci Sports Exerc*. Mar 1 2023;55(3):497-506. doi:10.1249/MSS.0000000000003078

105. Rodriguez-Nogueira O, Leiros-Rodriguez R, Benitez-Andrades JA, Alvarez-Alvarez MJ, Marques-Sanchez P, Pinto-Carral A. Musculoskeletal Pain and Teleworking in Times of the COVID-19: Analysis of the Impact on the Workers at Two Spanish Universities. *Int J Environ Res Public Health*. Dec 23 2020;18(1)doi:10.3390/ijerph18010031

106. Romero DE, Santana D, Borges P, et al. Prevalence, associated factors, and limitations related to chronic back problems in adults and elderly in Brazil. *Cad Saude Publica*. Mar 1 2018;34(2):e00012817. Prevalencia, fatores associados e limitacoes relacionados ao problema cronico de coluna entre adultos e idosos no Brasil. doi:10.1590/0102-311X00012817

107. Ryan C, Martin D, McDonough S, Leveille S, Kirwan J. Chronic musculoskeletal pain and physical activity in older adults: A secondary analysis of the health survey for England 2008. HUMAN KINETICS PUBL INC 1607 N MARKET ST, PO BOX 5076, CHAMPAIGN, IL 61820 …; 2012:S247-S248.

108. Salminen JJ, Oksanen A, Maki P, Pentti J, Kujala UM. Leisure time physical activity in the young. Correlation with low-back pain, spinal mobility and trunk muscle strength in 15-year-old school children. *Int J Sports Med*. Oct 1993;14(7):406-10. doi:10.1055/s-2007-1021200

109. Santos MCS, de Andrade SM, Gonzalez AD, Dias DF, Mesas AE. Association Between Chronic Pain and Leisure Time Physical Activity and Sedentary Behavior in Schoolteachers. *Behav Med*. Oct-Dec 2018;44(4):335-343. doi:10.1080/08964289.2017.1384358

110. Santos M, Gabani FL, Dias DF, et al. Longitudinal associations of changes in physical activity and TV viewing with chronic musculoskeletal pain in Brazilian schoolteachers. *PLoS One*. 2020;15(6):e0234609. doi:10.1371/journal.pone.0234609

111. Shiri R, Solovieva S, Husgafvel-Pursiainen K, et al. The role of obesity and physical activity in non-specific and radiating low back pain: the Young Finns study. *Semin Arthritis Rheum*. Jun 2013;42(6):640-50. doi:10.1016/j.semarthrit.2012.09.002

112. Shiri R, Falah-Hassani K, Heliovaara M, et al. Risk Factors for Low Back Pain: A Population-Based Longitudinal Study. *Arthritis Care Res (Hoboken)*. Feb 2019;71(2):290-299. doi:10.1002/acr.23710

113. Shiri R, Lallukka T, Rahkonen O, Leino-Arjas P. Excess Body Mass and Leisure Time Physical Activity in the Incidence and Persistence of Chronic Pain. *Pain Med*. Nov 1 2020;21(11):3094-3101. doi:10.1093/pm/pnaa102

114. Silva AG, Couto PS, Queiros A, Neto M, Rocha NP. Chronic pain in high school students is associated with physical activity and sleeping hours but not with screen time. *Int J Adolesc Med Health*. Jun 17 2017;31(3)doi:10.1515/ijamh-2017-0014

115. Skarpsno ES, Nilsen TIL, Sand T, Hagen K, Mork PJ. The joint effect of insomnia symptoms and lifestyle factors on risk of self-reported fibromyalgia in women: longitudinal data from the HUNT Study. *BMJ Open*. Aug 22 2019;9(8):e028684. doi:10.1136/bmjopen-2018-028684

116. Skogberg O, Karlsson L, Borsbo B, et al. Pain Tolerance in Chronic Pain Patients Seems to be More Associated with Physical Activity than with Depression and Anxiety. *J Rehabil Med*. Apr 29 2022;54:jrm00286. doi:10.2340/jrm.v54.241

117. Smith TO, Dainty JR, Williamson E, Martin KR. Association between musculoskeletal pain with social isolation and loneliness: analysis of the English Longitudinal Study of Ageing. *Br J Pain*. May 2019;13(2):82-90. doi:10.1177/2049463718802868

118. Sollerhed AC, Andersson I, Ejlertsson G. Recurrent pain and discomfort in relation to fitness and physical activity among young school children. *Eur J Sport Sci*. 2013;13(5):591-8. doi:10.1080/17461391.2013.767946

119. Solovev A, Watanabe Y, Kitamura K, et al. Total physical activity and risk of chronic low back and knee pain in middle-aged and elderly Japanese people: The Murakami cohort study. *Eur J Pain*. Apr 2020;24(4):863-872. doi:10.1002/ejp.1535

120. Soysal M, Kara B, Arda MN. Assessment of physical activity in patients with chronic low back or neck pain. *Turk Neurosurg*. 2013;23(1):75-80. doi:10.5137/1019-5149.JTN.6885-12.0

121. Stommen NC, Verbunt JA, Gorter SL, Goossens ME. Physical activity and disability among adolescents and young adults with non-specific musculoskeletal pain. *Disabil Rehabil*. 2012;34(17):1438-43. doi:10.3109/09638288.2011.645112

122. Stricevic J, Papez BJ. Non-specific low back pain: occupational or lifestyle consequences? *Wien Klin Wochenschr*. Dec 2015;127 Suppl 5:S277-81. doi:10.1007/s00508-015-0770-2

123. Suris X, Ortiz-Santamaria V, Pueyo-Sanchez MJ, Mompart-Penina A, Larrosa M, Ricart A. Decreasing prevalence of chronic back pain in Catalonia. Analysis of the Catalan Health Survey. *Public Health*. May 2022;206:38-45. doi:10.1016/j.puhe.2022.02.019

124. Swain MS, Henschke N, Kamper SJ, Gobina I, Ottova-Jordan V, Maher CG. Pain and Moderate to Vigorous Physical Activity in Adolescence: An International Population-Based Survey. *Pain Med*. May 1 2016;17(5):813-819. doi:10.1111/pme.12923

125. Teichtahl AJ, Urquhart DM, Wang Y, et al. Physical inactivity is associated with narrower lumbar intervertebral discs, high fat content of paraspinal muscles and low back pain and disability. *Arthritis Res Ther*. May 7 2015;17(1):114. doi:10.1186/s13075-015-0629-y

126. Thomas E, Silman AJ, Croft PR, Papageorgiou AC, Jayson MI, Macfarlane GJ. Predicting who develops chronic low back pain in primary care: a prospective study. *BMJ*. Jun 19 1999;318(7199):1662-7. doi:10.1136/bmj.318.7199.1662

127. Umeda M, Corbin LW, Maluf KS. Examination of contraction-induced muscle pain as a behavioral correlate of physical activity in women with and without fibromyalgia. *Disabil Rehabil*. 2015;37(20):1864-9. doi:10.3109/09638288.2014.984878

128. Umeda M, Kim Y. Gender Differences in the Prevalence of Chronic Pain and Leisure Time Physical Activity Among US Adults: A NHANES Study. *Int J Environ Res Public Health*. Mar 19 2019;16(6)doi:10.3390/ijerph16060988

129. van Oostrom SH, Monique Verschuren WM, de Vet HC, Picavet HS. Ten year course of low back pain in an adult population-based cohort--the Doetinchem cohort study. *Eur J Pain*. Oct 2011;15(9):993-8. doi:10.1016/j.ejpain.2011.02.007

130. van Oostrom SH, Smit HA, Wendel-Vos GC, Visser M, Verschuren WM, Picavet HS. Adopting an active lifestyle during adulthood and health-related quality of life: the Doetinchem Cohort Study. *Am J Public Health*. Nov 2012;102(11):e62-8. doi:10.2105/AJPH.2012.301008

131. van Weering MG, Vollenbroek-Hutten MM, Hermens HJ. The relationship between objectively and subjectively measured activity levels in people with chronic low back pain. *Clin Rehabil*. Mar 2011;25(3):256-63. doi:10.1177/0269215510380828

132. Vierola A, Suominen AL, Lindi V, et al. Associations of Sedentary Behavior, Physical Activity, Cardiorespiratory Fitness, and Body Fat Content With Pain Conditions in Children: The Physical Activity and Nutrition in Children Study. *J Pain*. Jul 2016;17(7):845-53. doi:10.1016/j.jpain.2016.03.011

133. Wadley AL, Iacovides S, Roche J, et al. Working nights and lower leisure-time physical activity associate with chronic pain in Southern African long-distance truck drivers: A cross-sectional study. *PLoS One*. 2020;15(12):e0243366. doi:10.1371/journal.pone.0243366

134. Whibley D, Guyer HM, Swanson LM, Braley TJ, Kratz AL, Dunietz GL. Sleep disturbance as a moderator of the association between physical activity and later pain onset among American adults aged 50 and over: evidence from the Health and Retirement Study. *BMJ Open*. Jun 7 2020;10(6):e036219. doi:10.1136/bmjopen-2019-036219

135. Yang H, Haldeman S. Behavior-Related Factors Associated With Low Back Pain in the US Adult Population. *Spine (Phila Pa 1976)*. Jan 1 2018;43(1):28-34. doi:10.1097/BRS.0000000000001665

136. Yiengprugsawan V, Hoy D, Buchbinder R, Bain C, Seubsman SA, Sleigh AC. Low back pain and limitations of daily living in Asia: longitudinal findings in the Thai cohort study. *BMC Musculoskelet Disord*. Jan 19 2017;18(1):19. doi:10.1186/s12891-016-1380-5

137. Zanuto EAC, Fernandes RA, Turi-Lynch BC, et al. Chronic low back pain and physical activity among patients within the Brazilian National Health System: a cross-sectional study. *Sao Paulo Med J*. Mar 2020;138(2):106-111. doi:10.1590/1516-3180.2019.0312.r1.19112019
